# Functional Neurons Generated from T Cell-Derived Induced Pluripotent Stem Cells for Neurological Disease Modeling

**DOI:** 10.1016/j.stemcr.2016.01.010

**Published:** 2016-02-18

**Authors:** Takuya Matsumoto, Koki Fujimori, Tomoko Andoh-Noda, Takayuki Ando, Naoko Kuzumaki, Manabu Toyoshima, Hirobumi Tada, Kent Imaizumi, Mitsuru Ishikawa, Ryo Yamaguchi, Miho Isoda, Zhi Zhou, Shigeto Sato, Tetsuro Kobayashi, Manami Ohtaka, Ken Nishimura, Hiroshi Kurosawa, Takeo Yoshikawa, Takuya Takahashi, Mahito Nakanishi, Manabu Ohyama, Nobutaka Hattori, Wado Akamatsu, Hideyuki Okano

**Affiliations:** 1 Department of Physiology, Keio University School of Medicine, Shinjuku-ku, Tokyo 160-8582, Japan; 2 Institute for Innovation, Ajinomoto Co., Inc., Kawasaki-ku, Kanagawa 210-8681, Japan; 3 Division of Medicine and Engineering Science, University of Yamanashi, Interdisciplinary Graduate School of Medicine and Engineering, Kofu, Yamanashi 400-8511, Japan; 4 Department of Pharmacology, Hoshi University, Pharmacy and Pharmaceutical Sciences, Shinagawa-ku, Tokyo 142-8501, Japan; 5 Laboratory for Molecular Psychiatry, RIKEN Brain Science Institute, Wako, Saitama 351-0198, Japan; 6 Department of Physiology, Yokohama City University Graduate School of Medicine, Kanazawa-ku, Kanagawa 236-0027, Japan; 7 Regenerative & Cellular Medicine Office, Sumitomo Dainippon Pharma Co., Ltd, Chuo-ku, Kobe 650-0047, Japan; 8 Department of Neurology, Juntendo University School of Medicine, Bunkyo-ku, Tokyo 113-8431, Japan; 9 Department of Dermatology, Keio University, School of Medicine, Shinjuku-ku, Tokyo 160-8582, Japan; 10 Biotechnology Research Institute for Drug Discovery, National Institute of Advanced Industrial Science and Technology (AIST), Tsukuba, Ibaraki 305-8565, Japan; 11 Laboratory of Gene Regulation, University of Tsukuba, Faculty of Medicine, Tsukuba, Ibaraki 305-8575, Japan; 12 Center for Genomic and Regenerative Medicine, Juntendo University, School of Medicine, Bunkyo-ku, Tokyo 113-8431, Japan

## Abstract

Modeling of neurological diseases using induced pluripotent stem cells (iPSCs) derived from the somatic cells of patients has provided a means of elucidating pathogenic mechanisms and performing drug screening. T cells are an ideal source of patient-specific iPSCs because they can be easily obtained from samples. Recent studies indicated that iPSCs retain an epigenetic memory relating to their cell of origin that restricts their differentiation potential. The classical method of differentiation via embryoid body formation was not suitable for T cell-derived iPSCs (TiPSCs). We developed a neurosphere-based robust differentiation protocol, which enabled TiPSCs to differentiate into functional neurons, despite differences in global gene expression between TiPSCs and adult human dermal fibroblast-derived iPSCs. Furthermore, neurons derived from TiPSCs generated from a juvenile patient with Parkinson's disease exhibited several Parkinson's disease phenotypes. Therefore, we conclude that TiPSCs are a useful tool for modeling neurological diseases.

## Highlights

• TiPSCs are a suitable source for neural disease modeling
• Developing a robust neurosphere-based induction protocol
• An optimized differentiation protocol can overcome clonal variations
• Modeling Parkinson's disease using TiPSC-derived neurons

## Teaser

In this article, Okano, Akamatsu, and colleagues show that an established neural induction protocol was able to induce neural cells from TiPSCs as well as fibroblast-derived iPSCs. We also observed that neurons derived from PARK2-TiPSCs can exhibit several Parkinson's disease-specific phenotypes observed in fibroblast-derived PARK2-iPSCs. Thus, T cells, which are easily accessible in patients, are suitable source for neural disease modeling.

## Introduction

Neurological diseases have mainly been studied using animal models and immortalized neural cell lines due to the difficulties associated with examining the CNS of patients. Recent advances in human induced pluripotent stem cell (hiPSC) technologies have enabled neurological diseases to be modeled by culturing patient-specific neural cells in dishes (Imaizumi and Okano, 2014, Marchetto and Gage, 2012). The first hiPSCs were generated from cultured dermal fibroblasts by inducing reprogramming factors (Takahashi et al., 2007). hiPSCs derived from fibroblasts have been recognized as the standard iPSCs for several years. Therefore, most previously reported patient-specific hiPSC lines were generated from skin fibroblasts (Brennand et al., 2011, Imaizumi et al., 2012). Skin biopsies of patients are required to generate dermal fibroblast lines, and this can cause bleeding, infection, and scarring. Therefore, patient-specific hiPSCs should ideally be generated using less invasive procedures, but the resulting cells must have a similar pluripotency as dermal fibroblast-derived hiPSCs.

Yamanaka and colleagues first reported that iPSCs can be generated from various types of somatic cells, including hepatocytes (Aoi et al., 2008). Since then, several groups have generated hiPSCs from peripheral blood nuclear cells (PBMC) (Loh et al., 2010, Mack et al., 2011, Seki et al., 2010), which can be easily obtained from patients using minimally invasive methods. Among these reports, Fukuda and colleagues showed that a small number of CD3-positive T cells can be efficiently reprogrammed into iPSCs using Sendai virus (SeV) vectors (Seki et al., 2010). CD3-positive T cells can be cultured in vitro using plates coated with an anti-CD3 monoclonal antibody (mAb) and in the presence of recombinant interleukin-2 (rIL-2). These cells can be stored in frozen vials and thawed several months later. Thus, CD3-positive T cells can be obtained non-invasively, are easily stored and efficiently reprogrammed, and might therefore be an ideal source of patient-specific iPSCs.

We sought to determine whether T cell-derived iPSCs (TiPSCs) could be used to analyze neurological diseases. Several issues regarding the utilization of TiPSCs in neurological studies remain unresolved. First, previous studies indicated that each iPSC clone retains an epigenetic memory relating to the cell type from which they are derived, even after their re-differentiation into somatic cells, and this restricts their differentiation potential (Kim et al., 2010, Kim et al., 2011, Panopoulos et al., 2012, Polo et al., 2010). Kim et al. reported that there are distinct differences in the genome-wide DNA methylation profiles of iPSCs derived from cord blood cells (CB-iPSCs) and iPSCs derived from neonate keratinocytes (K-iPSCs), and that these differences are closely related to their differentiation potentials. K-iPSCs had an enhanced potential to differentiate into keratinocytes in comparison with CB-iPSCs, even though both types of iPSCs were established from the same donor. Second, rearrangement of T cell receptor (TCR) chain genes in mature T cells indicates that they are not identical to naive lymphocytes at the genomic level. Although such rearrangements are reportedly retained in TiPSCs (Seki et al., 2010), it is unknown whether they affect the neural differentiation and function of TiPSCs.

In the present study, we showed that TiPSCs have a reduced tendency to differentiate into the neural lineage via embryoid body (EB) formation in comparison with adult human dermal fibroblast-derived iPSCs (aHDF-iPSCs). To overcome this, we established a neurosphere-based robust differentiation protocol that uses a low density of cells and hypoxic conditions. Using this method, TiPSCs efficiently and stably differentiated into mature functional neurons, similar to aHDF-iPSCs.

Furthermore, we demonstrated that TiPSC-derived neurons could be used as a Parkinson's disease model.

## Results

### Generation of Genetically Matched hiPSCs from T Cells and Skin Fibroblasts

To compare TiPSCs and aHDF-iPSCs in a similar genetic background (i.e., rearrangements of TCR chain genes), we generated these cells from T cells and dermal fibroblasts isolated from a healthy donor. TiPSCs (eTKA4, eTKA5, TKA7 [DNAVEC], TKA14 [DNAVEC], TKA4 [AIST], and TKA9 [AIST]) were generated from CD3-positive lymphocytes using episomal plasmid vectors (containing *OCT4*, *SOX2*, *KLF4, L-MYC*, *LIN28*, *EBNA1*, and sh*p53* or dominant-negative *p53*) (Okita et al., 2013) or SeV vectors, which were produced by DNAVEC Corp. (Cytotune) or the National Institute of Advanced Industrial Sciences and Technology (AIST). The DNAVEC SeV vector carried *OCT4*, *SOX2*, *KLF4*, or *c-MYC* on each of four vectors (Fusaki et al., 2009), whereas the AIST SeV vector carried all four reprogramming factors on a single vector (Nishimura et al., 2011). aHDF-iPSCs (KA11, KA23, eKA3, and eKA4) were also generated from the same healthy donor using retroviruses (*OCT4*, *SOX2*, *KLF4*, and *c-MYC*) or episomal plasmid vectors (Okita et al., 2013).

**Figure 1:**
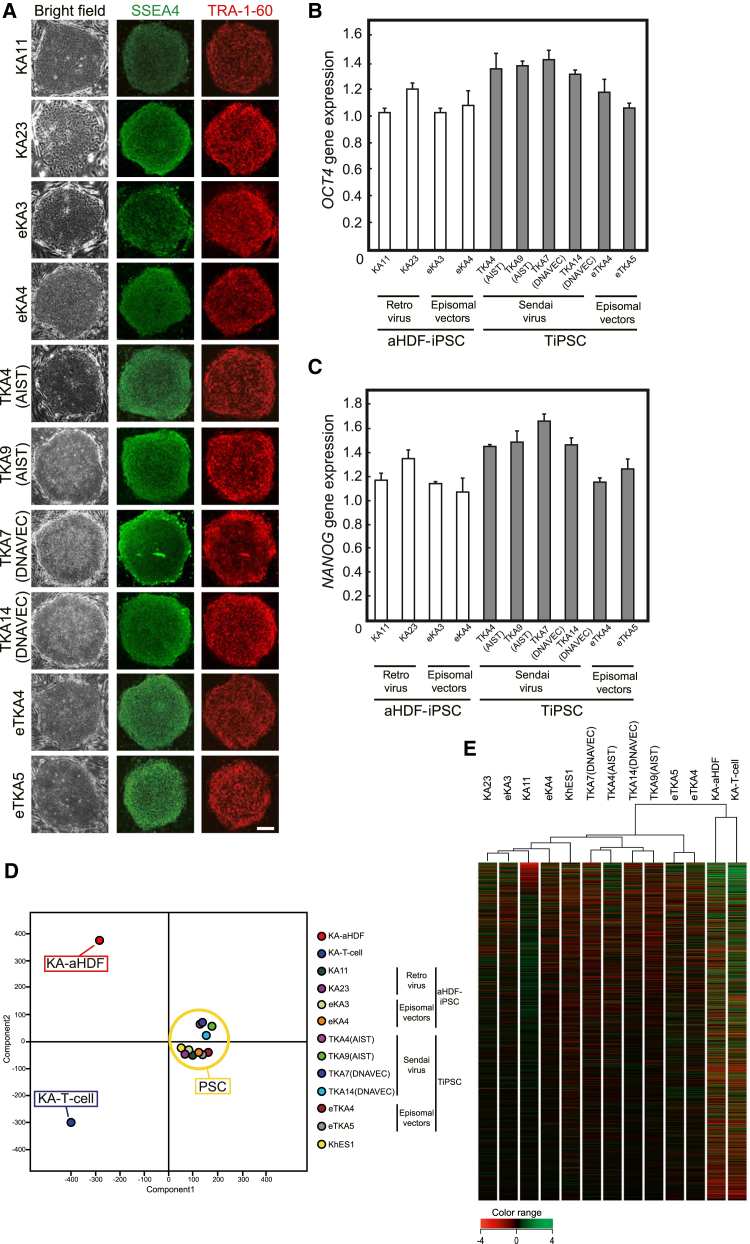
Characterization of iPSCs Derived from T Cells and Adult Human Dermal Fibroblasts (A) All of the iPSCs derived from KA-T cells and KA-aHDF were immunopositive for the pluripotency markers SSEA4 (green) and TRA-1-60 (red). (B and C) A comparison of the mRNA transcript levels in TiPSCs and aHDF-iPSCs by qPCR. The levels of endogenous *OCT4* (B) and endogenous *NANOG* (C) were comparable in the two types of iPSCs. The expression levels were normalized to the mean level in aHDF-iPSCs (set at 1.0) (n = 3 independent experiments; mean ± SD). (D) A comparison of the global gene expression profiles of aHDF-iPSCs, TiPSCs, and the original cells (KA-aHDF and KA-T cells) derived from a healthy man. A principal component analysis of the gene expression data. Yellow, PSC; red, KA-aHDF; blue, KA-T cells. (E) The results of a hierarchical clustering analysis of the global gene expression. See also Figure S1.

Immunocytochemical analysis revealed that all TiPSC clones expressed the pluripotency markers TRA-1-60 and stage-specific embryonic antigen 4 (SSEA4) (Figure 1A) at levels comparable with those in aHDF-iPSC clones. Expression of *OCT4* and *NANOG* was quantified by qPCR (Figures 1B and 1C). Transgenes used for reprogramming were not detected by RT-PCR (Figure S1A). The genomic structure did not significantly differ between TiPSCs and aHDF-iPSCs according to a comparative genomic hybridization array (Figure S1B). We next examined TCRβ rearrangements in genomic DNA of TiPSC clones to confirm that they were reprogrammed from CD3-positive T cells (Figure S1C). We amplified and analyzed TCRβ genomic regions by PCR and capillary electrophoresis. aHDF-iPSC clones (KA11 and PB2) did not show a specific positive peak indicative of TCRβ rearrangement in the Vβ/Jβ1,2, Vβ/Jβ2, or Dβ/Jβ region, whereas TiPSC clones (TKA4 [AIST], TKA9 [AIST], TPB4 [DNAVEC], and TPB8 [DNAVEC]) showed at least one specific peak in these regions. These results suggest that TiPSC clones were derived from CD3-positive T cells with TCRβ rearrangement.

### Comparison of the Biological Characteristics of TiPSCs and aHDF-iPSCs

To determine whether the origin of hiPSCs affects their global gene expression, we evaluated the global transcriptional profiles of TiPSCs and aHDF-iPSCs that were passaged fewer than 10–15 times using microarray analysis. Data, excluding genes whose expression values were low in all samples, were normalized and subjected to PCA and hierarchical clustering (Figures 1D and 1E). The samples could be crudely divided into three categories: T cells, aHDF, and pluripotent stem cells (PSC) (including TiPSCs, aHDF-iPSCs, and ESCs). Hierarchical clustering placed TiPSCs and aHDF-iPSCs into different groups (Figure 1E). On the other hand, TiPSCs generated using SeV vectors from DNAVEC Corp. (Fusaki et al., 2009) and those generated using SeV vector from AIST (Fusaki et al., 2009, Nishimura et al., 2011) did not cluster separately, and neither did aHDF-iPSCs generated using retroviruses and those generated using episomal plasmid vectors. The genome methylation profiles of TiPSC and aHDF-iPSC clones were compared by ChIP-seq analysis. Methylated genomic DNA was precipitated using a recombinant methyl-binding domain-containing protein and sequenced using a next-generation sequencer. The data were subjected to hierarchical clustering (Figure S1D). Consistent with the expression profiles, this analysis placed TiPSCs and aHDF-iPSCs into different groups according to the cell type from which they were derived. In addition, we identified 39 genes that were upregulated in the TiPSCs relative to aHDF-iPSCs (fold change >5.0). A gene ontology analysis identified a number of immune response-associated terms, suggesting that T cell-specific genes were still activated in the TiPSCs (Table S1). These data suggest that the cell type of origin influences the properties of the hiPSCs generated from the same donor.

### Compared with aHDF-iPSCs, the TiPSCs Were Poorly Differentiated into the Neural Lineage by an EB-Based Spontaneous Neural Differentiation Protocol

**Figure 2:**
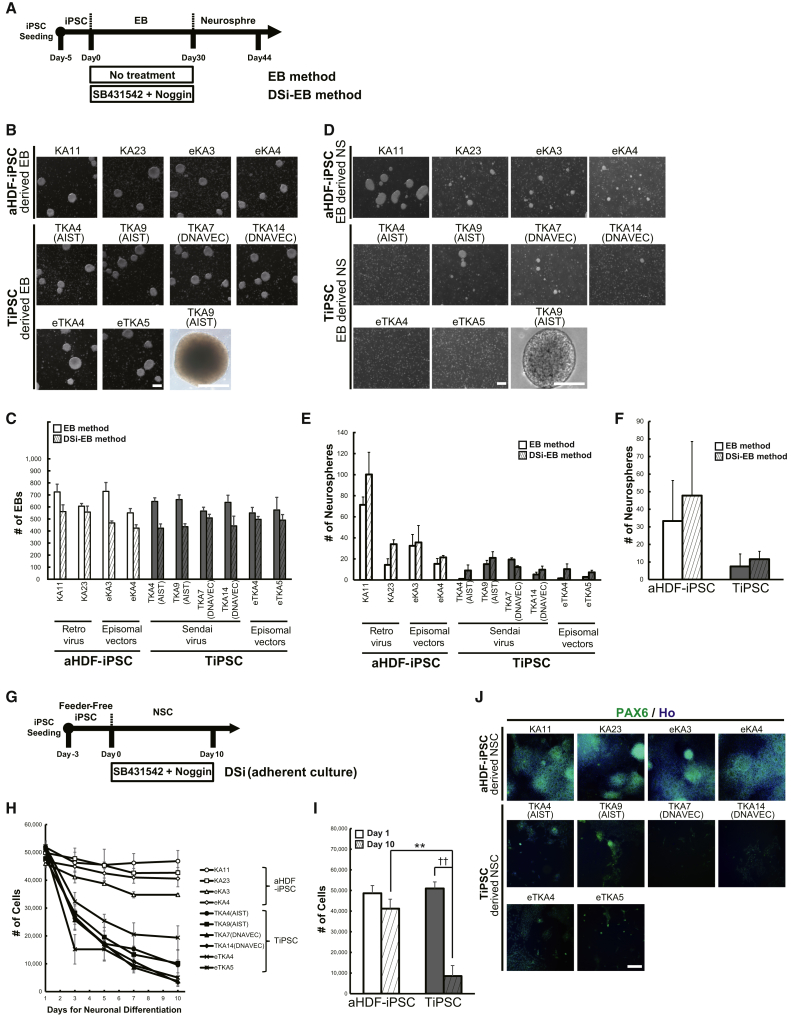
TiPSCs Could Differentiate into Only a Few Neural Lineage Cells when Induced by the Classical-EB, Modified-EB, and Dual SMAD Inhibition Protocols (A) An overview of the culture protocols for neurosphere induction via EB. EB formation with dual SMAD inhibition (DSi). (B) Images of floating EBs derived from aHDF-iPSCs and TiPSCs. Scale bars, 200 μm. (C) The number of EBs was determined by counting the spheres in which *D*_A_ > 50 μm (n = 4–6 independent experiments; mean ± SD). *D*_A_ denotes the diameter of the EB. (D) Images of floating neurospheres derived from aHDF-iPSC- and TiPSC-EBs. Scale bars, 200 μm. (E and F) The number of neurospheres was determined by counting the spheres in which *D*_A_ > 50 μm (n = 4–6 independent experiments; mean ± SD). *D*_A_ denotes the diameter of the neurospheres. (G) An overview of the culture protocol for neural stem cell (NSC) induction using DSi under feeder-free conditions. (H and I) The number of differentiating TiPSCs was significantly decreased compared with aHDF-iPSCs. ^∗∗^p < 0.01 when comparing aHDF-iPSCs and TiPSCs at day 10; ††p < 0.01 when comparing TiPSCs at day 1 and TiPSCs at day 10. The significance of the differences was assessed using Student's *t* test. At least three experiments were performed for each group. (J) Differentiated TiPSCs and aHDF-iPSCs were immunoreactive for the neural marker PAX6. See also Figures S2 and S3.

To confirm the impact of the original cell types on the iPSC differentiation, we evaluated whether TiPSCs could differentiate into neural stem/progenitor cells (NS/PCs) as efficiently as aHDF-iPSCs by using EB. In accordance with the previous studies using this method (Nori et al., 2011), we initially formed EBs from dissociated hiPSCs, and differentiated them into NS/PCs in a subsequent step (Figure 2A). When we cultured dissociated hiPSCs under the floating culture condition, all of the hiPSC lines converted EBs with a similar EB formation number (Figures 2B and 2C). We dissociated these EBs to expand NS/PCs in serum-free medium containing fibroblast growth factor 2 (FGF-2) and thereby generate neurospheres. All of the TiPSC lines we tested (TKA4 [AIST], TKA9 [AIST], TKA7 [DNAVEC], and TKA14 [DNAVEC]) formed few neurospheres, while all aHDF-iPSC lines were able to form neurospheres within 12 days (Figures 2D–2F).

We next tested a forced neural differentiation protocol using two inhibitors of SMAD signaling, SB431542 and Noggin, which were previously reported to induce dual SMAD inhibition (DSi) (Chambers et al., 2009) during EB formation. Although the EB method with DSi increased the average number of neurospheres formed compared with untreated control aHDF-iPSC (Figure 2F), the difference was not statistically significant because of the high level of variation among the iPSC lines. In contrast, the TiPSC-derived EBs formed very few neurospheres, even with DSi (Figures 2D–2F).

To assess the differences in the properties between aHDF-iPSC-EB and TiPSC-EB, we next quantified the expression levels of various genes in the neurospheres. qPCR analysis revealed that expression of the neural markers *PAX6*, *NESTIN*, *SOX1*, and *TUBB3* was much lower in TiPSC-derived EBs than in aHDF-iPSC-derived EBs (Figure S2B). However, the expression of mesendodermal (*BRACHYURY*) and endodermal (*SOX17*) markers was higher in TiPSC-derived EBs than in aHDF-iPSC-derived EBs (Figure S2C). DSi significantly upregulated the expression of *NESTIN* and *SOX1*, and slightly decreased the expression of *BRACHYURY* and *SOX17*, in aHDF-iPSC-derived EBs (Figures S2B and S2C). In contrast, the TiPSC-derived EBs were hardly affected by DSi, and the expression of neural markers remained much lower than that in the aHDF-iPSC-derived EBs (Figures S2B and S2C).

In an additional approach to neural differentiation, we cultured feeder-free TiPSCs under DSi conditions (Chambers et al., 2009). Dissociated iPSCs were plated on Matrigel-coated plates in complete conditioned media. After 3 days, further cultivation utilized knockout serum replacement media and/or N2 media containing 10 μM SB431542 and 200 ng/ml Noggin for 10 days (Figure 2G). We quantified the cell number during the differentiation period to assess the efficiency of neural differentiation. Although all aHDF-iPSC lines tested (KA11, KA23, eKA3, and eKA4) yielded a set number of cells during neural induction, all TiPSC lines (TKA4 [AIST], TKA9 [AIST], TKA7 [DNAVEC], TKA14 [DNAVEC], eTKA4 and eTKA5) showed a marked time-dependent decrease in cell number (Figures 2H and 2I). Immunocytochemical analysis revealed that both differentiated TiPSCs and aHDF-iPSCs expressed the neural marker PAX6, although the density of TiPSC-derived PAX6-positive cells was poor (Figure 2J).

We also tested whether the neural rosette method could induce NS/PCs from EBs (Koch et al., 2009). However, rosette-like cells derived from TiPSCs expressed few NS/PC markers (Figure S3). These data suggest that TiPSCs exhibit poor differentiation into neuro-ectoderm lineage compared with aHDF-iPSCs, even in the presence of dual SMAD inhibition.

### TiPSCs Can Be Differentiated into the Neural Lineage as Effectively as aHDF-iPSCs Using a Method with Direct Neurosphere Formation

**Figure 3:**
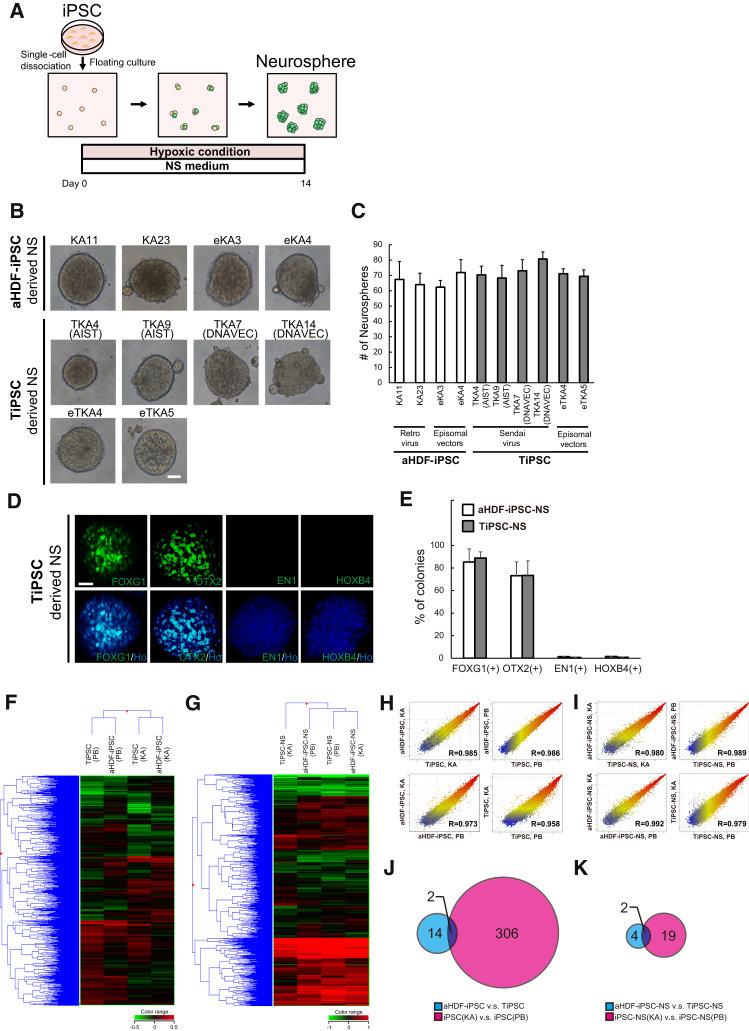
The Expression of Several Neuronal Markers Was Indistinguishable between Neurospheres Generated from TiPSCs and aHDF-iPSCs Using the Direct Neurosphere Converting Method (A) A schematic representation of the direct neurosphere converting method (dNS method). (B) Images of floating neurospheres derived from aHDF-iPSCs and TiPSCs obtained using the dNS method. Scale bars, 200 μm. (C) The number of neurospheres was determined by counting the spheres in which *D*_A_ > 50 μm (n = 4–6 independent experiments; mean ± SD). *D*_A_ denotes the diameter of the neurospheres. (D and E) The results of an immunocytochemical analysis of neurospheres for anteroposterior markers. (D) Representative images of anteroposterior marker-positive neurospheres. Scale bars, 200 μm. (E) The percentage of anteroposterior marker-positive neurosphere colonies. aHDF-iPSCs, KA11, KA23, and eKA3; TiPSCs, TKA4 (AIST), TKA7 (DNAVEC), and eTKA4 (n = 3 independent experiments; mean ± SD). (F–I) Comparison of the global gene expression profiles of TiPSCs (TKA4 [AIST], TKA9 [AIST], TPB4 [DNAVEC], and TPB8 [DNAVEC]) and aHDF-iPSCs (KA23, eKA3, PB2, and PB20) and tertiary neurospheres derived from them. (F and G) Hierarchical clustering analysis of global gene expression. (H and I) Comparison of the global gene expression patterns. (J and K) Venn diagram of human genes whose expression increased or decreased in the aHDF-iPSC/TiPSC, iPSC (KA)/iPSC (PB), and their NSs groups (moderated *t* test, p < 0.05, fold change >2.0). See also Figures S4 and S5.

TiPSCs preferentially differentiated into mesodermal and endodermal cells, not ectodermal cells, during EB formation (Figure S2). The absence of ectodermal cells in EBs reduced the formation of neurospheres from dissociated EBs. To overcome this differentiation preference of TiPSCs, we developed a method to induce neurons from iPSCs that does not involve EB formation (Figure 3A). It has been reported that low-density dissociation of mouse ES cells, in the absence of serum, resulted in most of the cells expressing neural markers (Tropepe et al., 2001). Therefore, we simply dissociated hiPSCs into single cells and cultured these cells in serum-free medium to induce ectodermal cells.

Dissociated hiPSCs are vulnerable to apoptosis. To avoid this, we added Y27632, which promotes the survival of single hiPSCs (Watanabe et al., 2007), and cultured cells in hypoxic conditions, which promotes the survival of NS/PCs by inhibiting apoptosis (Clarke and van der Kooy, 2009). As expected, these conditions enhanced the number of neurospheres formed (Figure S4). Neurospheres formed after 14 days of culture in medium containing FGF-2, Y27632, and human leukemia inhibitory factor (hLIF) under low-oxygen conditions (Figure 3A). We compared the efficiency of neural induction between TiPSCs and aHDF-iPSCs using this direct neurosphere (dNS) method. The numbers of neurospheres induced from TiPSCs (TKA4 [AIST], TKA9 [AIST], TKA7 [DNAVEC], TKA14 [DNAVEC], eTKA4, and eTKA5) and aHDF-iPSCs (KA11, KA23, eKA3, and eKA4) did not significantly differ (Figures 3B and 3C).

To clarify the regional identity of the neurospheres formed by this dNS method, we next examined the expression of anteroposterior markers in hiPSC-derived neurospheres by an immunocytochemical analysis. Both the TiPSC- and aHDF-iPSC-derived neurospheres mostly expressed the forebrain marker, FOXG1 (Oliver et al., 1995), and the forebrain/midbrain marker, OTX2 (Simeone et al., 1992) (Figures 3D and 3E). However, only a few of these neurospheres expressed EN1, which is expressed in the mesencephalon and the metencephalon (Hanks et al., 1995), or HOXB4, a marker of the myelencephalon and the spinal cord (Hunt et al., 1991) (Figures 3D and 3E). These results indicated that both the TiPSC- and aHDF-iPSC-derived neurospheres were similarly differentiated into the anterior region around the forebrain/midbrain by this dNS method.

To confirm the robustness of the dNS method in the neural differentiation of TiPSCs, we generated TiPSCs from two additional donors (FK and PB); FK cells derived from another healthy donor, and PB cells derived from a patient with a PARK2 mutation, which is known to cause juvenile Parkinson's disease (PARK2). We had previously established fibroblast-derived hiPSCs from this patient (Imaizumi et al., 2012). Using the dNS method, the numbers of neurospheres were not significantly decreased and differentiated similarly even if they were generated from the various TiPSC clones (TFK7 [DNAVEC], TFK12 [DNAVEC], TPB4 [DNAVEC], TPB8 [DNAVEC], TPB11 [DNAVEC], and TPB27 [DNAVEC]) (Figure S5).

**Figure 4:**
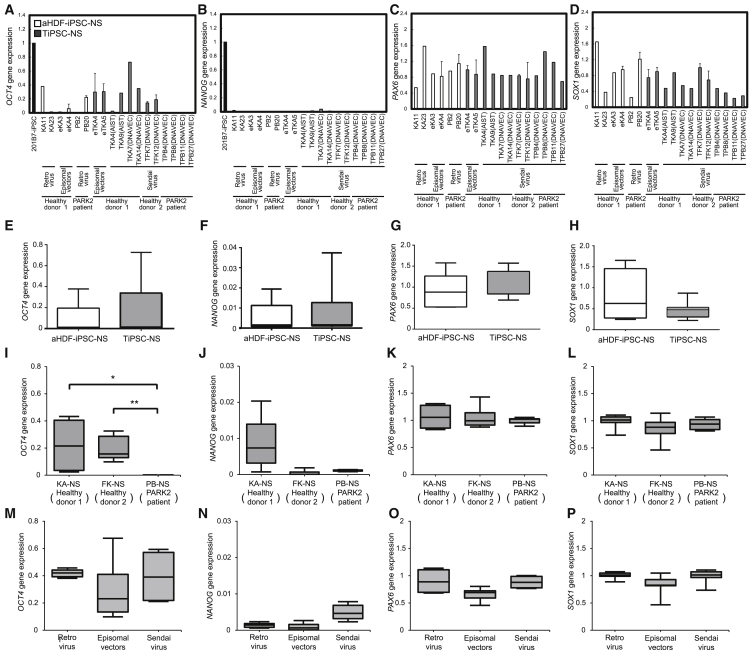
Comparison of mRNA Transcript Levels among Neurospheres Generated from the Various Types of Induced Pluripotent Stem Cells by Quantitative Reverse-Transcription PCR (A–D) Levels of pluripotent markers (*OCT4* and *NANOG*) and neural stem markers (*PAX6* and *SOX1*) in tertiary neurospheres generated from each iPSC clone. Expression levels were normalized to the mean level of each gene in 201B7-iPSCs, which was a previously established human iPSC clone (Takahashi et al., 2007) (*OCT4* and *NANOG*), or neural stem/progenitor cells derived from aHDF-iPSCs (*PAX6* and *SOX1*) (set at 1.0) (n = 3 independent experiments; mean ± SD). (E–H) Statistical analysis of mRNA transcript levels in tertiary neurospheres derived from aHDF-iPSCs and TiPSCs. Expression of pluripotency and neural stem markers was similar in these two types of cells. (I–L) The results of a statistical analysis of the mRNA transcript levels in tertiary neurospheres derived from KA, FK, and PB clones. The *OCT4* expression was lower in neurospheres derived from the PB clones than in neurospheres derived from the other donors' clones. ^∗∗^p < 0.01 and ^∗^p < 0.05 compared with the control. The significance of differences was assessed using Tukey's test. (M–P) The results of a statistical analysis of the mRNA transcript levels in tertiary neurospheres derived from hiPSCs generated using different methods to transduce the Yamanaka factors: Retroviruses, Episomal vectors, and Sendai virus. The expression levels of markers of pluripotency and neural stems were similar among the three types of cells tested.

iPSCs favor differentiation along the lineage of the cell type from which they are derived (Kim et al., 2010, Kim et al., 2011, Polo et al., 2010). However, Yamanaka and colleagues reported that hepatic differentiation of hiPSCs is largely influenced by differences in the donor, rather than by the cell type from which they are derived (Kajiwara et al., 2012). We sought to determine how donor differences and the original cell type influence the neural differentiation of hiPSCs. The global transcriptional profiles of iPSCs and iPSC-derived neurospheres generated from T cells or fibroblasts of the healthy donor and PARK2 patient (KA23, eKA3, TKA4 [AIST], TKA9 [AIST], PB2, PB20, TPB4 [DNAVEC], and TPB8 [DNAVEC]) were examined. The gene expression analysis of iPSCs showed that there were differences in their properties associated with the original cell types and donors, while the analysis of NSs obtained using the dNS method demonstrated that these differences among iPSCs, especially those caused by different genetic backgrounds, were smaller (Figures 3F–3I). In addition, as shown in the Venn diagrams in Figures 3J and 3K, which classified the cells based on the up- or downregulated genes in comparison with aHDF-iPSC/TiPSC, iPSC (KA)/iPSC (PB), and their NSs, the number of altered genes was also much lower in the NSs than that in the iPSCs (aHDF-iPSC versus TiPSC, 16 genes; iPSC(KA) versus iPSC(PB), 308 genes; aHDF-iPSC-NS versus TiPSC-NS, six genes; iPSC-NS(KA) versus iPSC-NS(PB), 21 genes; moderated t test, p < 0.05, fold change >2.0). These analyses indicate that NS induction by the dNS method decreases the variations in the iPSC properties caused by the differences in the original cell types and donors. Furthermore, we included six additional clones and quantified the expression of pluripotency (*OCT4* and *NANOG*) and NS/PC (*PAX6* and *SOX1*) markers by qPCR (Figures 4A–4D). The expression of *OCT4* was lower in neurospheres derived from PB clones than in neurospheres derived from the other donors' clones (Figures 4A and 4I). However, there was no difference in the expression of *OCT4* between the cells based on the cell origins or the methods used for iPSC derivation (Figures 4E and 4M). Furthermore, the expression of *NANOG* and NS/PC markers did not significantly differ among the various neurospheres, regardless of the original cell types, donors, or methods used to generate iPSCs (Figures 4B–4D, 4F–4H, 4J–4L, and 4N–4P). These results indicate that the dNS method could lead to the formation of neurospheres from hiPSCs, independent of the cell types of origin, donors, and iPSC generation methods, and could minimize the differences in the differentiation propensities of each hiPSC line.

### TiPSCs Can Be Differentiated into Functional Neurons and Cells of Various Neuronal Subtypes to a Similar Extent as aHDF-iPSCs Using the dNS Method

**Figure 5:**
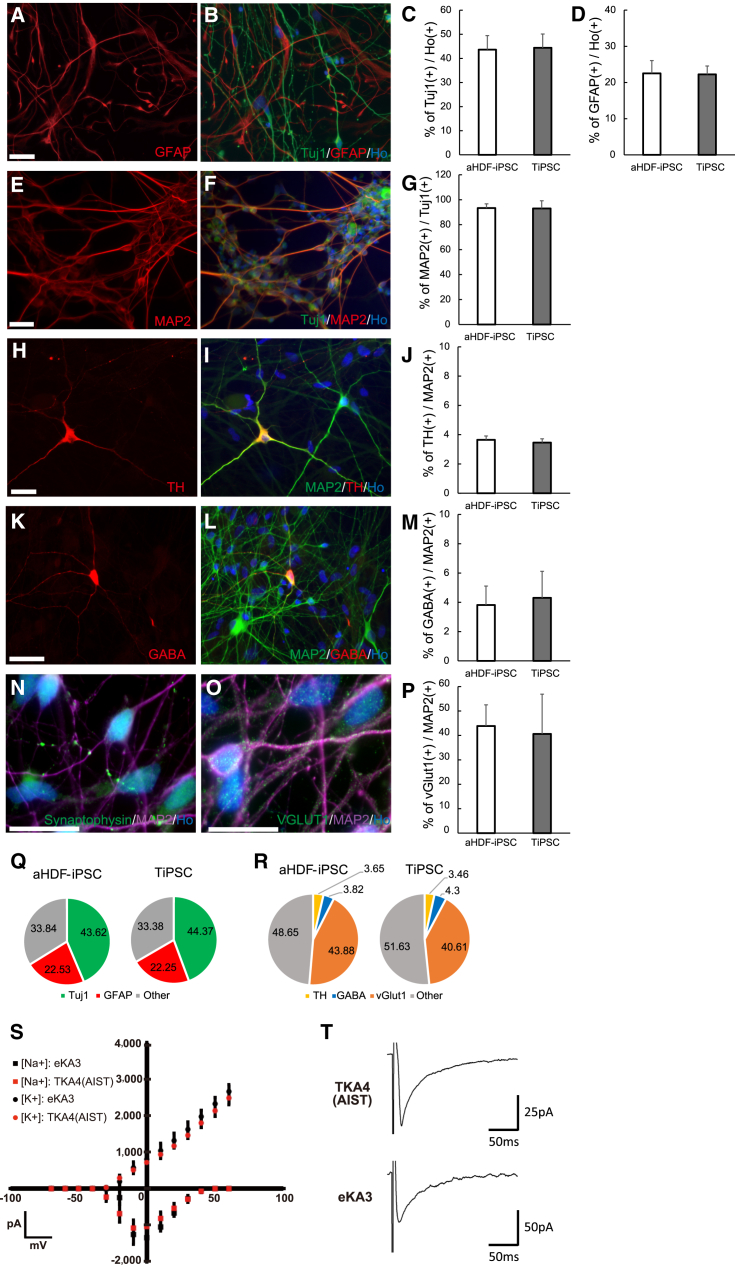
Neurospheres Generated from TiPSCs Have the Potential to Differentiate into Specific Neuronal Subtypes and Could Be Differentiated into Electrophysiologically Functional Neurons Using the dNS Method (A–P) Differentiation potential was assessed in media hormone mix containing B27 for 30–60 days. Neural cells derived from TiPSCs (TKA4 [AIST] and TKA7 [DNAVEC]) were immunoreactive for MAP2, βIII-tubulin (Tuj1), GFAP, TH, GABA, VGLUT1, and synaptophysin. Scale bars, 50 μm (n = 5 independent experiments; mean ± SD). (Q and R) A summary of the ratio of cell types and neuronal subtypes differentiated from aHDF-iPSCs and TiPSCs. (S) Current-voltage plot of sodium and potassium currents of neurons derived from TKA4 [AIST] and eKA3 clones. (T) Voltage-clamp recordings of neurons derived from TKA4 [AIST] and eKA3 clones showing evoked synaptic currents. See also Figure S6.

We next examined whether NS/PCs derived from TiPSCs could differentiate into functional neuronal subtypes as efficiently as NS/PCs derived from aHDF-iPSCs. Neurospheres were plated on fibronectin- and poly-L-ornithine (PO)-coated plates and differentiated in media hormone mix (MHM) containing B27, 10 ng/ml brain-derived neurotrophic factor (BDNF), 10 ng/ml glial cell-derived neurotrophic factor (GDNF), 200 μM ascorbic acid, and 1 mM dibutyryl-cAMP for 30–70 days. After 60 days, the differentiated cells expressed the neural marker, microtubule-associated protein 2 (MAP2), another neural marker, βIII-tubulin (Tuj1), and an astrocyte marker, glial fibrillary acidic protein (GFAP), with similar differentiation ratios observed between the TiPSCs and aHDF-iPSCs, indicating that they had equally differentiated into neurons and astrocytes (Figures 5A–5G). An immunocytochemical analysis revealed that these MAP2-positive neurons included tyrosine hydroxylase (TH)-positive dopaminergic neurons (Figures 5H–5J), γ-aminobutyric acid (GABA)-positive GABAergic neurons (Figures 5K–5M), and vesicular glutamate transporter 1-positive glutamatergic neurons that expressed synaptophysin (Figures 5N–5P). Synaptophysin had a punctate distribution, indicative of synaptic formation in vitro.

Figures 5Q and 5R show summaries of the data for these differentiated cell types and neuronal subtypes, which suggested that the TiPSCs could differentiate into various kinds of cells as efficiently as aHDF-iPSCs. The electrophysiological properties of the TiPSC-derived neurons were examined to confirm that they were functional. We recorded voltage-sensitive currents in 30- to 60-day-old TiPSC- and aHDF-iPSC-derived neurons. iPSC-derived neurospheres were infected with a lentivirus expressing human synapsin promoter-driven GFP (CSIV–hSynI-GFP-IRES2-NeoR) (Zhou et al., 2014) and differentiated into neurons. TTX-sensitive voltage-gated membrane currents were indistinguishable between neurons derived from TiPSCs and those derived from aHDF-iPSCs (Figure S6A). Na^+^ and K^+^ currents also had similar current-voltage relationships in TiPSC-derived neurons and aHDF-iPSC-derived neurons (Figure 5S). Current-clamp measurements showed that these two types of neurons both generated multiple action potentials (Figure S6B). We next investigated whether TiPSC-derived neurons could form synapses. Evoked postsynaptic currents were observed in whole-cell voltage-clamp recordings of TiPSC-derived neurons (Figure 5T). These results show that the dNS method can differentiate TiPSCs into functional neurons, and that the electrophysiological function of these neurons is indistinguishable from that of aHDF-iPSC-derived neurons.

### Neurons Derived from TiPSCs Established from a PARK2 Patient Exhibited Several Different Parkinson's Disease Phenotypes

**Figure 6:**
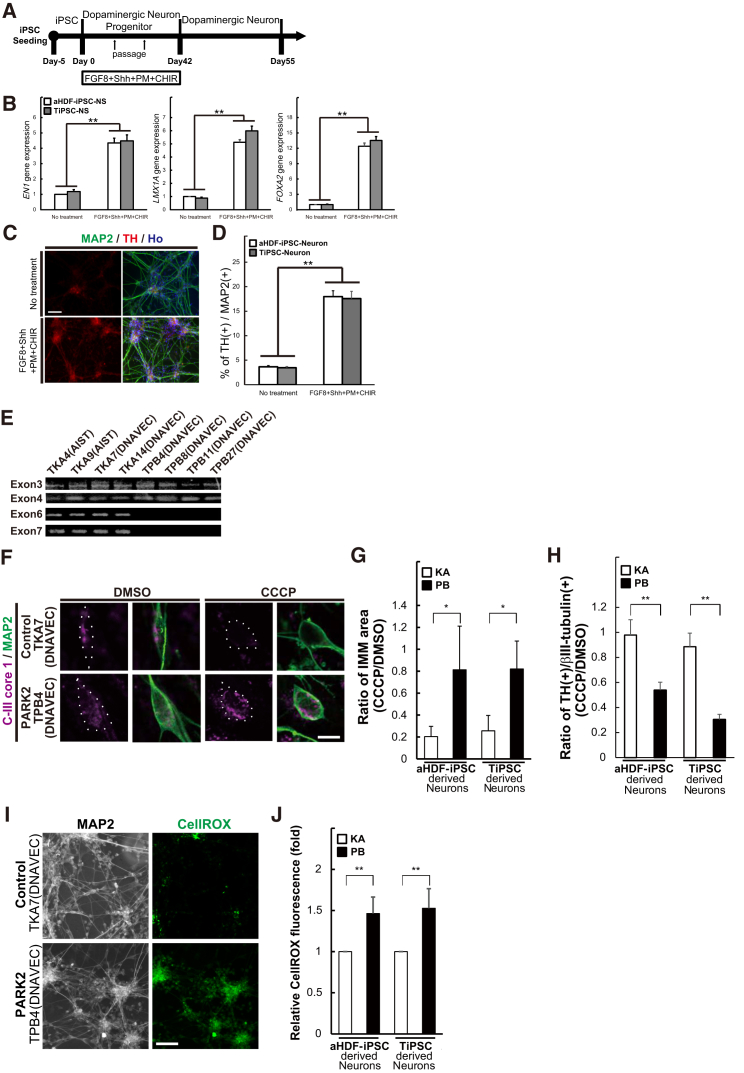
Neurons Derived from TiPSCs Established from a PARK2 Patient Exhibited Several Parkinson's Disease Phenotypes: Impairment of Mitochondrial Functions, Dopaminergic Neuron-Specific Cell Death, and Increased ROS Production (A) An overview of the culture protocol used for the induction of midbrain dopaminergic neurons (mDANs) from hiPSCs. (B) The results of a qPCR analysis of tertiary neurospheres treated with or without the four small molecules for markers of mDAN, including *EN1*, *LMX1A*, and *FOXA2*. (C and D) The results of an immunocytochemical analysis of the tertiary neurospheres treated with or without the four small molecules for a dopaminergic neuronal marker, TH. Three experiments were performed for each group. Scale bars, 50 μm. (E) Deletion of exons 6 and 7 was confirmed in clones TPB4 (DNAVEC), TPB8 (DNAVEC), TPB11 (DNAVEC), and TPB27 (DNAVEC). (F and G) Carbonyl cyanide *m*-chlorophenyl hydrazone (CCCP) treatment reduced the inner mitochondrial membrane area (IMM, magenta) in control neurons, but not in PARK2 neurons. The area of the IMM in KA clone-derived MAP2-positive neurons (eKA3, TKA4 [AIST], TKA7 [DNAVEC]) was reduced following CCCP treatment. White dotted lines indicate cell bodies of neural cells. Scale bars, 25 μm. This reduction was not observed in PARK2 patient-derived MAP2-positive neurons (PB2, TPB4 [DNAVEC], and TPB8 [DNAVEC]). ^∗^p < 0.05 compared with the control. Significant differences were assessed using Student's t test. At least three experiments were performed per group, with 10–12 cells quantified per experiment. (H) CCCP treatment significantly decreased the ratio of TH-positive neurons in PARK2 neurons compared with control neurons. At least three experiments were performed for each group, with 200–300 cells quantified per experiment. (I and J) The results of an oxidative stress analysis using CellROX Green Reagent. PARK2 neurons showed increased fluorescence of CellROX compared with control neurons. At least three experiments were performed for each group, with 200–300 cells quantified per experiment. Scale bars, 50 μm. (B), (D), (H), (J) ^∗∗^p < 0.01 compared with the control. The significance of differences was assessed using Student's *t* test. Three experiments were performed for each group. aHDF-iPSCs, KA11, KA23, PB2, and PB20; TiPSCs, TKA7 [DNAVEC], TKA14 [DNAVEC], TPB4 [DNAVEC], and TPB8 (DNAVEC).

To determine whether hiPSCs established from the peripheral blood of patients can be used as a model of neurological diseases, we differentiated TiPSCs derived from a patient with PARK2, a familial form of Parkinson's disease, into neurons. A consistent neurochemical abnormality found in Parkinson's disease is the degeneration of dopaminergic neurons in the substantia nigra. Therefore, we first modified the dNS method to generate midbrain dopaminergic neuron (mDAN)-enriched culture by treating the cells with several small molecules, FGF-8, sonic hedgehog (Shh), purmorphamine (PMA), and CHIR99021 (CHIR) during the NS formation, for a total of 42 days (Figure 6A). qPCR showed that there was upregulation of markers of mDAN differentiation, including *EN1*, *LMX1A*, and *FOXA2* (Figure 6B). After they were allowed to mature for 13 days, immunostaining of the neural epithelial cells demonstrated that nearly 20% of the MAP2-positive cells expressed the dopaminergic neuron marker, TH (Figures 6C and 6D). These data related to the expression pattern marked the acquired TH-positive neurons as mDANs (Figures 6B–6D).

The PARK2 patient-derived TiPSCs (TPB4 [DNAVEC], TPB8 [DNAVEC], TPB11 [DNAVEC], and TPB27 [DNAVEC]) had a homozygous deletion in exons 6 and 7 of the *PARK2* gene (Figure 6E), expressed the human pluripotent markers TRA-1-60 and SSEA4 (Figure S5), and could differentiate into dopaminergic neurons (Figure S5). Parkin is a component of an E3 ubiquitin ligase involved in mitochondrial homeostasis (Shimura et al., 2000). We previously reported that the hiPSCs established from fibroblasts of a PARK2 patient (the same patient used as the source of TiPSCs in the current study) exhibited an abnormal turnover of damaged mitochondria (Imaizumi et al., 2012) and increased reactive oxygen species (ROS) production in the neurons. Therefore, we first treated TiPSC-derived neurons with carbonyl cyanide *m*-chlorophenyl hydrazone (CCCP), which triggers the loss of mitochondrial membrane potential and results in the removal of damaged mitochondria. To determine the extent to which damaged mitochondria were eliminated after CCCP treatment, we visualized the area of the inner mitochondrial membrane (IMM) using an antibody against the IMM marker Complex-III core I. Compared with untreated samples, CCCP treatment elicited a dramatic decrease in the IMM area in control neurons (TKA clones) but not in PARK2 neurons (TPB clones) (Figures 6F and 6G).

Using the same neuronal samples treated with CCCP, we next stained the cells for TH and quantified the ratio of TH-positive neurons to evaluate the vulnerability of dopaminergic neurons to mitochondrial stress. We observed that there was a decrease in the number of TH-positive neurons in PARK2 neuronal cultures (TPB clones) due to the treatment with CCCP (Figure 6H), indicating that the dopaminergic neurons derived from PARK2-iPSCs were more vulnerable to mitochondrial stress compared with control-iPSCs.

Finally, we evaluated the ROS production in the neurons derived from control- and PARK2-TiPSCs using the CellROX Green Reagent, which is weakly fluorescent while in a reduced state and exhibits bright green photostable fluorescence upon oxidation by ROS, with absorption/emission maxima of ∼485/520 nm. The reactive CellROX fluorescence was significantly increased in the PARK2 neurons, indicating that there was increased ROS production (Figures 6I and 6J). These phenotypes were also observed in the neurons derived from aHDF-iPSCs (Figures 6F–6J). These results strongly suggest that the hiPSCs derived from patient T cells can be used as a model of neurological disease.

## Discussion

hiPSCs can be used to generate neuronal cells in vitro, which has widened the application of these cells. However, the requirement for skin biopsies to be performed must be overcome before iPSC technologies are widely adopted. Seki et al. (2010) developed TiPSCs, which enabled iPSCs to be obtained more easily and with less invasive methods. The current study shows that TiPSCs can differentiate into functional neurons, including dopaminergic, GABAergic, and glutamatergic subtypes, using an optimized differentiation protocol, dNS method. Moreover, these cells could be used to study the mechanisms underlying neuronal diseases, although there are some differences between TiPSCs and aHDF-iPSCs. Furthermore, the neurons differentiated from TiPSCs derived from a PARK2 patient exhibited several Parkinson's disease phenotypes, including an impairment of mitochondrial functions, dopaminergic neuron-specific cell death, and increased ROS production.

Most patients with neurological disorders, such as Parkinson's disease, do not have a genetic background associated with the onset of the disease. Recent whole-exome sequencing studies revealed that such sporadic diseases are related to many rare variants. To elucidate the etiologies of these diseases, it is important to establish hiPSCs from a sufficient number of patients and to characterize multiple clones such that statistical analyses can be performed. T cells are a suitable source of hiPSCs to model sporadic neurological diseases owing to the ease with which they can be obtained. In this article, we have shown that neurons generated from TiPSCs exhibit phenotypes similar to those of aHDF-iPSCs modeling Parkinson's disease. We will therefore address in future work the question of whether TiPSCs can be used in the study of other neurological diseases.

Some studies indicate that iPSCs retain a transient transcriptional and epigenetic memory of their cell types of origin, which can substantially affect their potential to differentiate into various cell types, even among iPSCs that are genetically identical (Kim et al., 2010, Kim et al., 2011, Polo et al., 2010). Two independent studies of genetically matched hiPSC clones established from skin or blood cells examined how their differentiation potential is influenced by their cell types of origin. Daley and colleagues reported that blood cell-derived iPSCs differentiate into blood cells more efficiently than keratinocyte-derived iPSCs, and the latter iPSCs efficiently differentiate into keratinocytes (Kim et al., 2011). They concluded that the differentiation potential of hiPSCs is influenced by a residual epigenetic memory of the tissue from which they are derived. They also showed that extended passage of most iPSC lines reduced the effect of cell origin by erasing the cells' epigenetic memory, although some clones failed to erase the epigenetic memory, even after recurrent passaging. It is possible that our protocol could minimize the differences stemming from differences in epigenetic status after any passage of iPSCs, which we will address in a future study. However, a study by Yamanaka and colleagues reported that blood cell-derived iPSCs and skin cell-derived iPSCs can both differentiate into the hepatic lineage if they are established from the same donor (Kajiwara et al., 2012). They concluded that variations in hepatic differentiation are largely owing to differences in the donors, rather than to the cell types from which iPSCs are derived. Although these two conclusions seem contradictory, it is possible that the influences of epigenetic memory and donor differences vary according to the lineage along which cells differentiate. Here, we showed that TiPSCs failed to differentiate along the neural lineage using the EB formation protocol. The increased expression of mesendodermal and endodermal markers in TiPSC-derived EBs suggests that TiPSCs preferentially differentiated into the mesendodermal lineage, rather than the ectodermal lineage. Our single-cell dissociation protocol enabled TiPSCs to differentiate into functional neuronal cells with a similar efficiency as aHDF-iPSCs. Furthermore, TiPSC-derived neuronal cells generated from a Parkinson's disease patient exhibited abnormal mitochondrial degradation similar to aHDF-iPSC-derived neuronal cells. Our results suggest that this robust directed differentiation protocol can overcome the biased differentiation of iPSCs owing to epigenetic memory of the original cells. Our results also suggest that TCR rearrangement does not affect the differentiation efficiency when a directed neural differentiation method is used. EBs contain cells that have differentiated from PSCs into the three germ cell lineages in a spontaneous and unbiased manner (Itskovitz-Eldor et al., 2000). However, TiPSC-derived EBs contained few neural cells due to their reduced potential to differentiate into the neural lineage. By contrast, our differentiation protocol facilitated directed neural differentiation by removing extracellular signals. In relation to genetic background, Yamanaka and colleagues suggested that the propensity for hepatic differentiation of iPSCs is affected by differences in the donor, rather than in the original cell types (Kajiwara et al., 2012). Our results suggest that differences in the donors, original cell types, and iPSC generation methods can be minimalized by using this differentiation protocol.

Recently, several studies have suggested that peripheral blood mononuclear cells (PBMCs) are a promising resource for generating iPSCs because most of these cells do not undergo TCR rearrangement (Loh et al., 2010, Mack et al., 2011, Seki et al., 2010). However, PBMCs are composed of several cell types (e.g., T cells, B cells, and CD34 + progenitor cells), and each PBMC has an epigenetic memory of the cell from which it is derived. It is possible that PBMC-derived iPSCs restrict neural differentiation in a manner similar to TiPSCs. Since our present neural differentiation protocol was able to minimize the effect of cell origin, our protocol could be presumably applicable to iPSCs derived from other blood cell types.

Here, we suggest that the method of neural induction is the most important factor in the study of neural cells derived from human TiPSCs. The optimized induction protocol allows various iPSC clones to differentiate into functional neuronal cells, regardless of the cell types from which they are derived. Therefore, TiPSCs can be used to study neurological diseases and to recapitulate disease-specific phenotypes, comparable with iPSCs derived from fibroblasts or other cells, when generated using the optimized differentiation protocol. We propose that T cells are an ideal source of patient-specific iPSCs and will be widely used for neurological disease modeling. We believe that our present neural differentiation protocol will minimize the effect of cell origin and is applicable to iPSCs derived from other tissues. For example, urine epithelial cells could be an excellent alternate source for the generation of iPSCs because monocytes without TCR rearrangement can also be obtained in a less invasive fashion (Song et al., 2011, Zhang et al., 2015, Zhou et al., 2011). These possibilities for modeling neurological diseases should be investigated in the future.

## Experimental Procedures

### Neural Differentiation In Vitro

A previously reported protocol was used for neural differentiation of hiPSCs (Nori et al., 2011). Briefly, hiPSCs were dissociated and cultured in suspension as EBs, which were subsequently dissociated and cultured in MHM supplemented with B27 (Life Technologies), 20 ng/ml fibroblast growth factor 2 (FGF-2, Peprotech), and 10 ng/ml hLIF (Millipore).

Neural rosette differentiation was performed as previously described (Koch et al., 2009). Four-day-old EBs were transferred to poly-L-ornithine (PO)-coated tissue culture dishes and propagated in ITSfn medium (DMEM/F-12 [Wako] containing 25 μg/ml insulin [Wako], 100 μg/ml transferrin [Nakalai-tesque], 5 ng/ml selenite [Sigma-Aldrich], and 2.5 μg/ml fibronectin [Sigma-Aldrich]). Within 10 days, neural tube-like structures developed in the EB outgrowth.

For neural induction from single hiPSCs, hiPSCs were incubated with TrypLE Select (Life Technologies) for 5 min and dissociated into single cells by pipetting. Cells were plated into a T75 flask (Nunclon), then 10^3^ cells were plated for the neurosphere formation assay, and cultured in MHM supplemented with B27, 20 ng/ml FGF-2, 10 μM Y-27632 (Wako), and 10 ng/ml hLIF in 4% oxygen for 14 days. Neurospheres were repeatedly passaged by dissociation into single cells, and then cultured in the same manner. Neurospheres at passages 3–7 were typically used for analysis. For terminal differentiation, dissociated neurospheres were allowed to adhere to PO- (Sigma-Aldrich) and fibronectin-coated coverslips and cultured in MHM containing B27, 10 ng/ml brain-derived neurotrophic factor (BDNF; R&D systems), 10 ng/ml glial cell-derived neurotrophic factor (GDNF; R&D systems), 200 μM ascorbic acid (Sigma-Aldrich), and 1 mM dibutyryl-cAMP (Sigma-Aldrich) for 10–70 days.

### Ethics

For the use of human samples, human ethics approval was obtained by the Ethics Committee of Keio University School of Medicine.

## Author Contributions

T.M., K.F., W.A., and H.O. conceived and designed the experiments. T.M., K.F., T.A.-N., T.A, N.K. R.Y., M.T., H.T., K.I., M. Ishi, and M. Iso. performed the experiments and analyzed data. T.M., K.F., W.A., and H.O. wrote and edited the manuscript. T.K., M. Ohtaka, M. Ohyama, K.N., M.N., S.S., N.H., T.Y., Z.Z., H.K., and T.T. contributed reagents, materials, and analysis tools. All authors read and approved the final manuscript.

## Accession Numbers

The accession number for both the microarray data and genomic methylation data reported in this paper is GEO: GSE76832.

## Supplemental Information

Document S1. Supplemental Experimental Procedures, Figures S1–S6, and Tables S1–S3

Document S2. Article plus Supplemental Information
