# Supplementary material for: Functional Neurons Generated from T Cell-Derived Induced Pluripotent Stem Cells for Neurological Disease Modeling
Source: Stem Cell Reports. 2016 Feb 18;6(3):422–35. doi: 10.1016/j.stemcr.2016.01.010 (PMC4788773; doi:10.1016/j.stemcr.2016.01.010)
Supplement: Document S1. Supplemental Experimental Procedures, Figures S1–S6, and Tables S1–S3 [file mmc1.pdf]

Stem Cell Reports, Volume *6*

Supplemental Information

Functional Neurons Generated from T Cell-Derived Induced Pluripotent Stem Cells for Neurological Disease Modeling

Takuya Matsumoto, Koki Fujimori, Tomoko Andoh-Noda, Takayuki Ando, Naoko Kuzumaki, Manabu Toyoshima, Hirobumi Tada, Kent Imaizumi, Mitsuru Ishikawa, Ryo Yamaguchi, Miho Isoda, Zhi Zhou, Shigeto Sato, Tetsuro Kobayashi, Manami Ohtaka, Ken Nishimura, Hiroshi Kurosawa, Takeo Yoshikawa, Takuya Takahashi, Mahito Nakanishi, Manabu Ohyama, Nobutaka Hattori, Wado Akamatsu, and Hideyuki Okano

Supplemental Figure Legends

Fig. S1 Characterization of the genomic profile of iPSCs derived from T-cells and adult human dermal fibroblasts

(A) A reverse-transcription PCR analysis showed the loss of the Sendai-virus (SeV) sequence in TiPSCs.

(B)The copy number profiles of whole chromosomes in TiPSCs and aHDF-iPSCs were assessed by a comparative genomic hybridization microarray analysis. There was no evidence that genomic aberrations were introduced during the process of establishing these iPSCs.

(C) Characterization of the T-cell receptor-β rearrangement by capillary electrophoresis. The green line was derived from the band of the Jβ1 gene, and the blue line was derived from the band of the Jβ2 gene. TKA4(AIST) showed rearrangements in Vβ/Jβ2 and Dβ/Jβ. TKA9(AIST) showed rearrangements in Vβ/Jβ1, 2. TPB4(DNAVEC) showed rearrangements in Vβ/Jβ2 and Dβ/Jβ. TPB8(DNAVEC) showed rearrangements in Vβ/Jβ1, 2 and Dβ/Jβ.

(D) Comparison of the global genomic methylation profiles of aHDF-iPSCs (KA11 and KA23) and TiPSCs (TKA4(AIST) and TKA9(AIST)). Hierarchical clustering analysis demonstrated the clear separation of aHDF-iPSCs and TiPSCs into two clusters.

Fig. S2. Comparison of the mRNA transcript levels in the EBs or DSi-EBs derived from TiPSCs and those derived from aHDF-iPSCs determined by qPCR.

(A) Overview of the culture protocols used for this experiment. (B) The expression of neural stem markers (*TUBB3*, *NESTIN*, *SOX1,* and *PAX6*) was higher, and (C) the expression of the mesendodermal marker *BRACHYURY* and the endodermal marker *SOX17* was lower in EBs derived from aHDF-iPSCs than in those derived from TiPSCs, even in the presence of dual SMAD inhibition (DSi) (n=3 independent experiments; mean ± SD; **P* < 0.05, ***P* < 0.01; Student's *t*-test).

Fig. S3. Comparison of mRNA transcript levels in rosettes generated from TiPSCs and aHDF-iPSCs by quantitative reverse-transcription PCR. The expression of neural stem markers (*PAX6*, *NESTIN*, *SOX1*, *DACH1*, and *ZNF312*) was higher in aHDF-iPSCs than in TiPSCs, (n=5 independent experiments; mean ± SD.).

Fig. S4. The neurosphere formation using the dNS method was dependent on Y27632, and was enhanced by culturing the cells under reduced oxygen conditions (n=5 independent experiments; mean ± SD.).

Fig. S5. Characterization of TiPSCs derived from a PARK2 patient (PB) and a healthy donor (FK). All of the TPB clones and TFK clones were immunopositive for the pluripotent markers, SSEA4 (Green) and TRA-1-60 (Red). They also were differentiated into βIII-tubulin-positive neurons, including TH-positive dopaminergic neurons, via neurosphere formation using a slightly modified dNS method. Scale bars: 200 μm

(SSEA4 immunostaining, TRA-1-60 immunostaining, and neurospheres) and 50 μm (neurons).

Fig. S6. Electrophysiological analysis of TiPSC-derived neurons in comparison to aHDF-iPSC-derived them. (A) Voltage-dependent sodium and potassium currents in neurons derived from TKA4(AIST) and eKA3 clones. (B) Representative traces of TiPSC- and aHDF-iPSC-derived neurons membrane potential responding to step depolarization (-10 pA to +70 pA) by current injection (TiPSC: TKA4(AIST), aHDF-iPSC: eKA3)

Table S1. The gene ontology analysis of transcripts upregulated in TiPSC (eTKA4) vs aHDF-iPSC (eKA3). Only significant and non-redundant categories are shown (*p* value < 0.01) , related to Figure 1.

Table S2. Antibodies used for immunocytochemistry, related to Figures 1, 2, 3, 5, 6, S5

Table S3. Sequences of primers used for genomic PCR, quantitative PCR, and detection of the Sendai virus vector, related to Figures 6 and S1

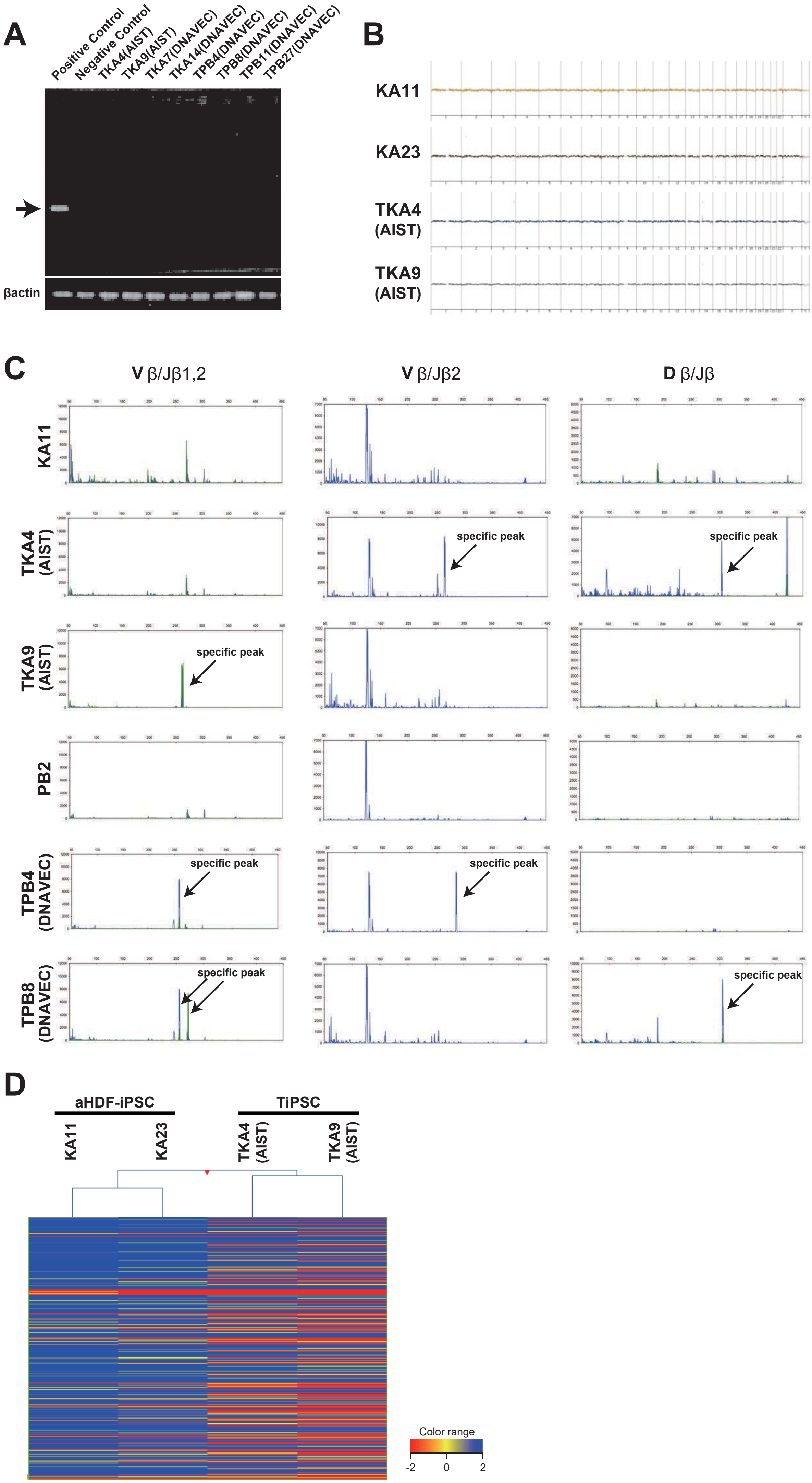
A
Positive Control
Negative Control
TKA4(AIST)
TKA9(AIST)
TKA7(DNAVEC)
TKA14(DNAVEC)
TPB4(DNAVEC)
TPB8(DNAVEC)
TPB11(DNAVEC)
TPB27(DNAVEC)
βactin
B
KA11
KA23
TKA4 (AIST)
TKA9 (AIST)
C
V β/Jβ1,2
V β/Jβ2
D β/Jβ
KA11
TKA4 (AIST)
TKA9 (AIST)
PB2
TPB4 (DNAVEC)
TPB8 (DNAVEC)
specific peak
D
aHDF-iPSC
TiPSC
KA11
KA23
TKA4 (AIST)
TKA9 (AIST)
Color range
-2
0
2

Fig.S1

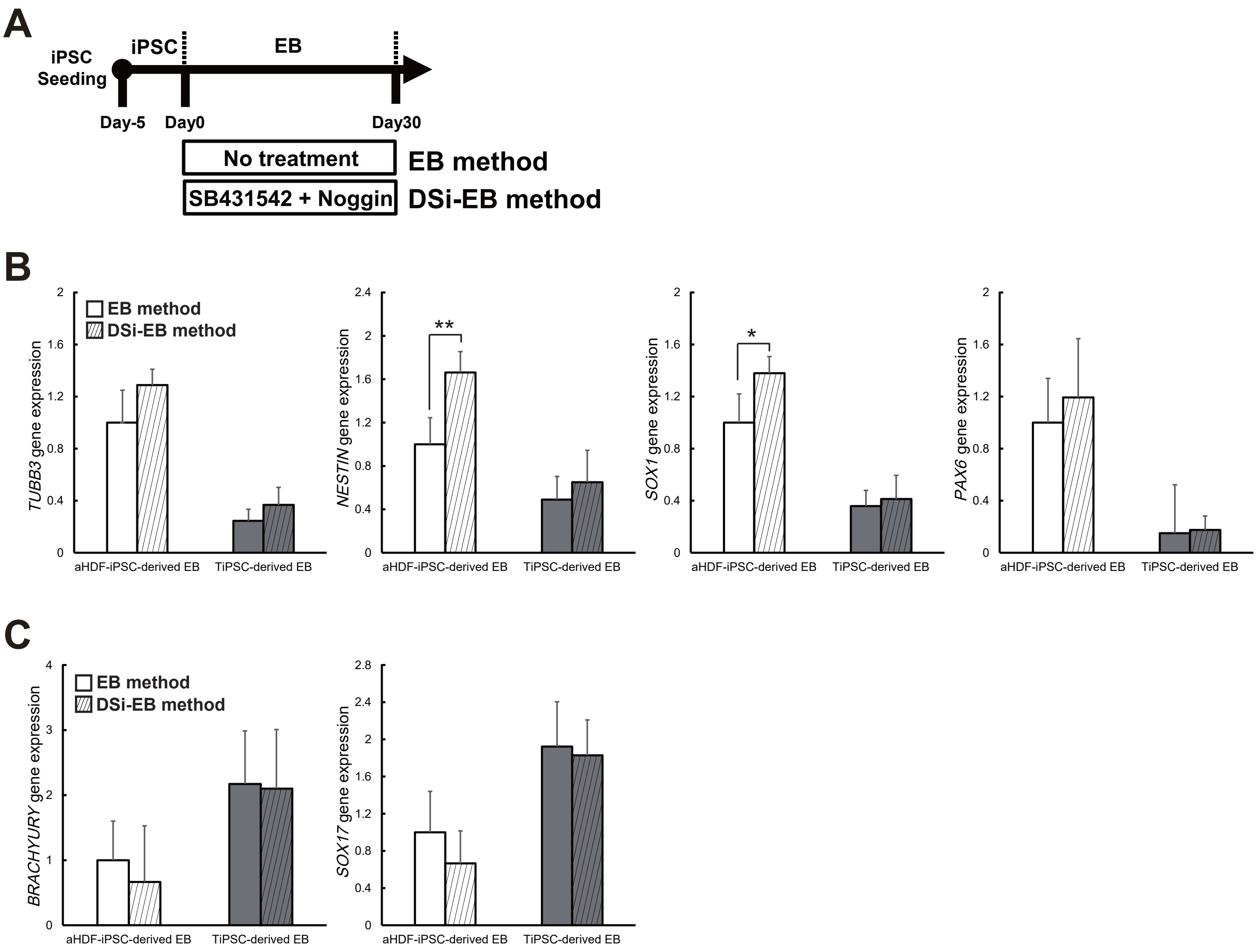
A
iPSC Seeding
iPSC
EB
Day-5
Day0
Day30
No treatment
EB method
SB431542 + Noggin
DSi-EB method
B
EB method
DSi-EB method
TUBB3 gene expression
NESTIN gene expression
SOX1 gene expression
PAX6 gene expression
aHDF-iPSC-derived EB
TiPSC-derived EB
C
BRACHYURY gene expression
SOX17 gene expression

Fig.S2

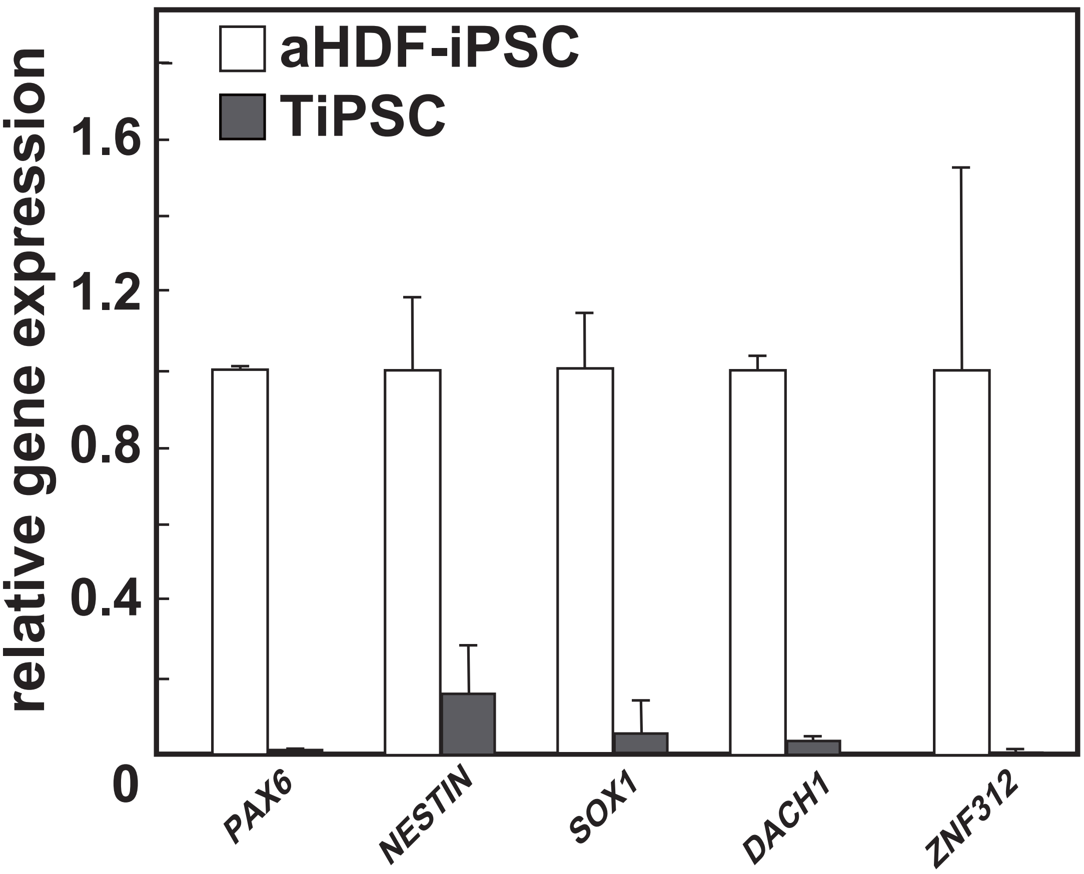
aHDF-iPSC
TiPSC
relative gene expression
1.6
1.2
0.8
0.4
0
PAX6
NESTIN
SOX1
DACH1
ZNF312

Fig.S3

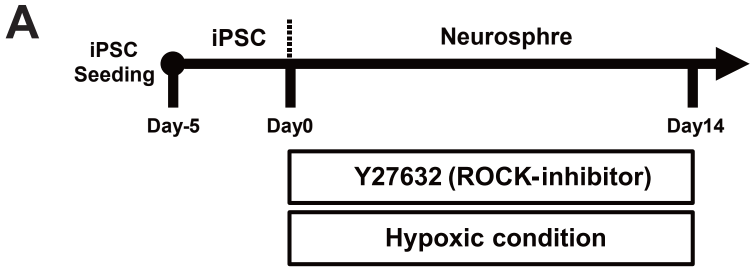
A
iPSC
Seeding
iPSC
Neurosphre
Day-5
Day0
Day14
Y27632 (ROCK-inhibitor)
Hypoxic condition

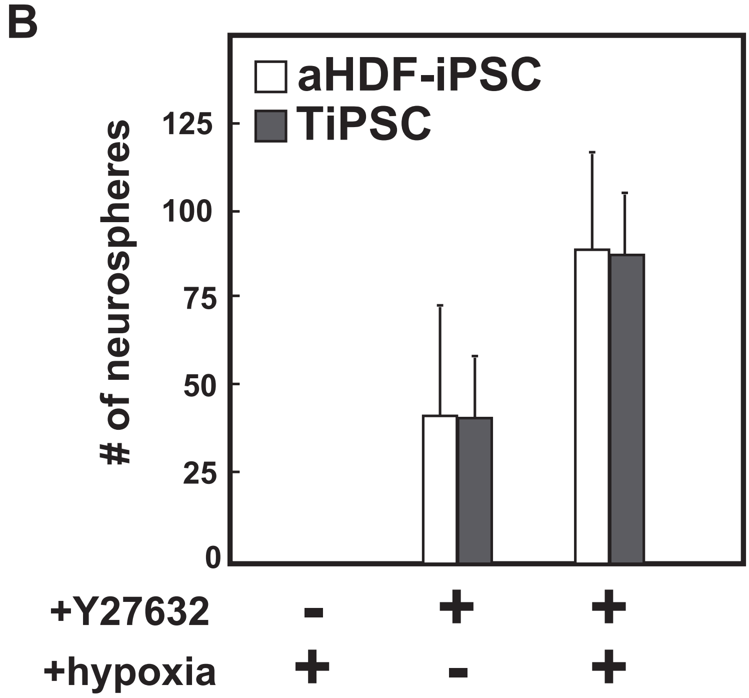
B
aHDF-iPSC
TiPSC
# of neurospheres
125
100
75
50
25
0
+Y27632
+hypoxia
-
+
+
+
-
+

Fig.S4

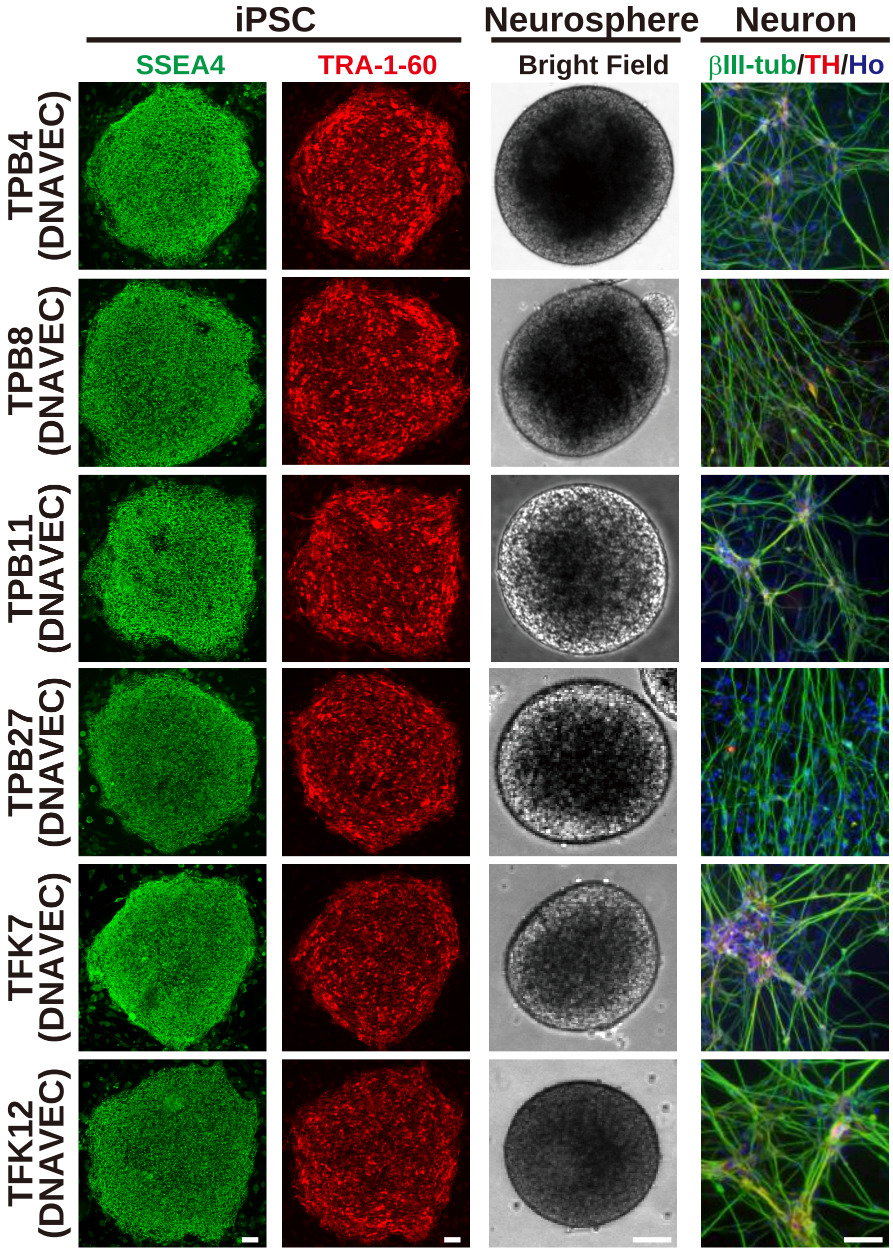
iPSC
Neurosphere
Neuron
SSEA4
TRA-1-60
Bright Field
βIII-tub/TH/Ho
TPB4 (DNAVEC)
TPB8 (DNAVEC)
TPB11 (DNAVEC)
TPB27 (DNAVEC)
TFK7 (DNAVEC)
TFK12 (DNAVEC)

Fig.S5

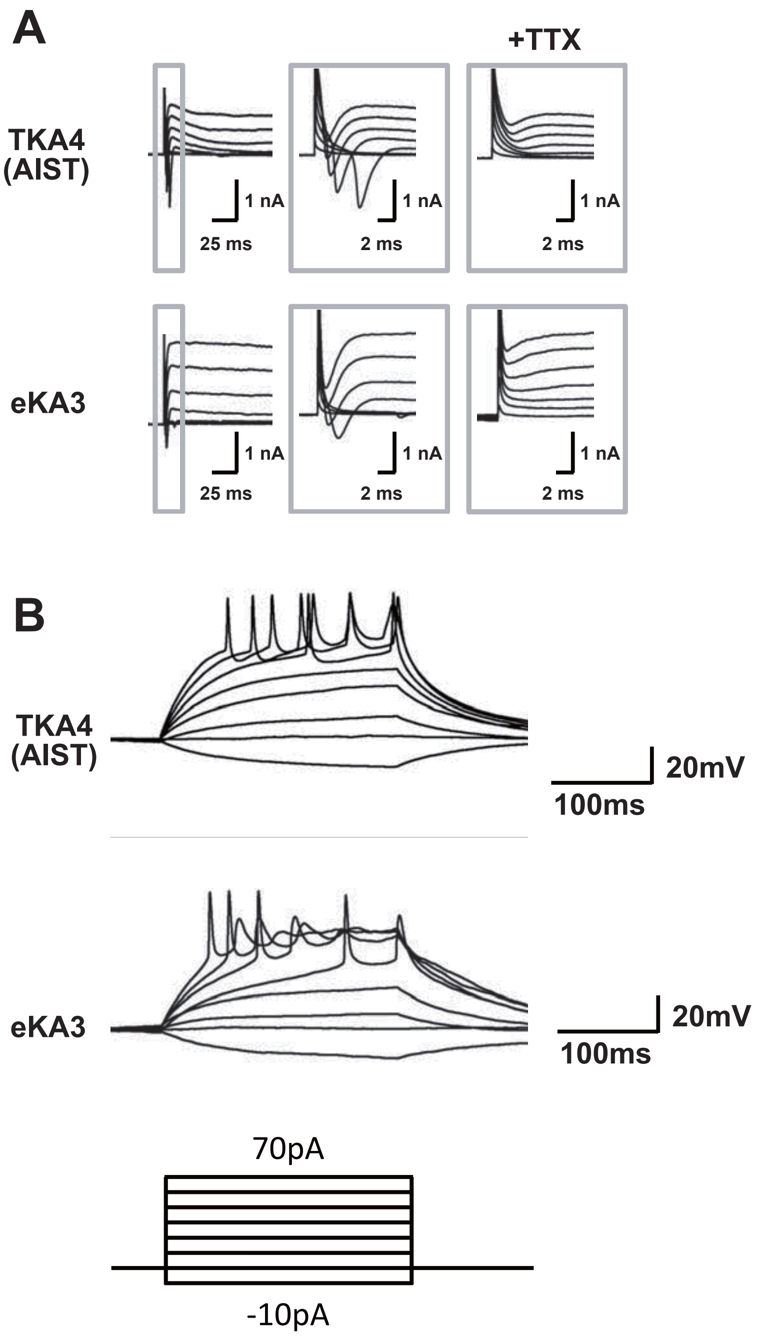
A
+TTX
TKA4
(AIST)
1 nA
25 ms
1 nA
2 ms
1 nA
2 ms
eKA3
1 nA
25 ms
1 nA
2 ms
1 nA
2 ms
B
TKA4
(AIST)
20mV
100ms
eKA3
20mV
100ms
70pA
-10pA

Fig.S6

GO ACCESSION	GO Term	corrected p-value
GO:0050896|GO:0051869	response to stimulus	2.35E-09
GO:0051716	cellular response to stimulus	2.70E-05
GO:0048583	regulation of response to stimulus	2.90E-05
GO:0010033	response to organic substance	4.18E-05
GO:0007165|GO:0023033	signal transduction	4.35E-05
GO:0042605|GO:0042606|GO:0042607	peptide antigen binding	7.22E-05
GO:0044700	single organism signaling	9.22E-05
GO:0023052|GO:0023046	signaling	9.22E-05
GO:0005685	U1 snRNP	9.22E-05
GO:0048518|GO:0043119	positive regulation of biological process	1.13E-04
GO:0006952|GO:0002217|GO:0042829	defense response	1.56E-04
GO:0006955	immune response	1.77E-04
GO:0007154	cell communication	2.04E-04
GO:0005515|GO:0045308	protein binding	2.56E-04
GO:0005686	U2 snRNP	2.56E-04
GO:0006950	response to stress	3.02E-04
GO:0044763	single-organism cellular process	3.21E-04
GO:0060968	regulation of gene silencing	3.28E-04
GO:0050778	positive regulation of immune response	3.53E-04
GO:0002429	immune response-activating cell surface receptor signaling pathway	3.71E-04
GO:0050776	regulation of immune response	4.67E-04
GO:0009719	response to endogenous stimulus	5.88E-04
GO:0003823	antigen binding	7.78E-04
GO:0007166	cell surface receptor signaling pathway	0.001009211
GO:0071310	cellular response to organic substance	0.001226054
GO:0055037	recycling endosome	0.001245703
GO:0034314	Arp2/3 complex-mediated actin nucleation	0.00135526
GO:0050789|GO:0050791	regulation of biological process	0.00135526
GO:0070887	cellular response to chemical stimulus	0.001424034
GO:0044699	single-organism process	0.001428615
GO:0065007	biological regulation	0.001428615
GO:0002768	immune response-regulating cell surface receptor signaling pathway	0.00162319
GO:0055038	recycling endosome membrane	0.00162319
GO:0002376	immune system process	0.001824635
GO:0002682	regulation of immune system process	0.0020203
GO:0004872|GO:0019041	receptor activity	0.0020203
GO:0042221	response to chemical	0.002038446
GO:0043014	alpha-tubulin binding	0.002862832
GO:0031295	T cell costimulation	0.002919185
GO:0009725	response to hormone	0.002942639
GO:0071944	cell periphery	0.003043802
GO:0048584	positive regulation of response to stimulus	0.003043802
GO:0031294	lymphocyte costimulation	0.00306639
GO:0045010	actin nucleation	0.003145426
GO:0032879	regulation of localization	0.003830532
GO:0045834	positive regulation of lipid metabolic process	0.004213654
GO:0005886|GO:0005904	plasma membrane	0.004333442
GO:0042060	wound healing	0.005252767
GO:0044459	plasma membrane part	0.005423789
GO:0002757	immune response-activating signal transduction	0.005748065
GO:0046983	protein dimerization activity	0.005748065
GO:0002684	positive regulation of immune system process	0.006020954
GO:0050794|GO:0051244	regulation of cellular process	0.006485721
GO:0050851	antigen receptor-mediated signaling pathway	0.006765673
GO:0051239	regulation of multicellular organismal process	0.006765784
GO:0042611	MHC protein complex	0.006801865
GO:0071407	cellular response to organic cyclic compound	0.006972198
GO:0051249	regulation of lymphocyte activation	0.007141397
GO:0050852	T cell receptor signaling pathway	0.007170936
GO:0005887	integral component of plasma membrane	0.008697007
GO:0048522|GO:0051242	positive regulation of cellular process	0.008868844
GO:0002253	activation of immune response	0.009484018

Table S1

Antibody	Dilution	Source	Catalogue number	Location
TRA-1-60	1:1000	Millipore	MAB4360	Billerica, MA
SSEA4	1:1000	Abcam	ab16287	Cambridge, MA
PAX6	1:500	BioLegend	901301	San Diego, CA
βIII-tubulin	1:2000	Sigma-Aldrich	T8660	St Louis, MI
MAP2	1:1000	Sigma-Aldrich	M4403	St Louis, MI
VGLUT1	1:4000	Synaptic Systems	135 303	Germany
GFAP	1:4000	DAKO	Z033401	Glostrup, DK
Synaptophysin	1:1000	Sigma-Aldrich	S5768	St Louis, MI
GABA	1:2000	Sigma-Aldrich	A2052	St Louis, MI
TH	1:500	Millipore	AB152	Billerica, MA
FOXG1	1:250	Abcam	ab18259	Cambridge, MA
OTX2	1:100	R&D Systems	AF1979	Minneapolis, MN
EN1	1:100	Abcam	ab117549	Cambridge, MA
HOXB4	1:100	DSHB	I12	Iowa City, IA
Complex III-core I	1:200	Thermo Fisher Scientific	459140	Waltham, MA

SSEA4, stage-specific embryonic antigen 4; MAP2, microtubule-associated protein 2; VGLUT1, vesicular glutamate transporter 1; GFAP, glial fibrillary acidic protein; GABA, gamma-aminobutyric acid; TH, tyrosine hydroxylase

Table S2

Name	Sequence
	**For genomic PCR**
Ex3 forward	ACATGTCACTTTTGCTTCCCT
Ex3 reverse	AGGCCATGCTCCATGCAGACTGC
Ex4 forward	AGGTAGATCAATCTACAACAGCT
Ex4 reverse	CTGGGTCAAGGTGAGCGTTGCCTGC
Ex6 forward	AGAGATTGTTTACTGTGGAAACA
Ex6 reverse	GAGTGATGCTATTTTTAGATCCT
Ex7 forward	GAGCCCCGTCCTGGTTTTCC
Ex7 reverse	CCACACAAGGCAGGGAGTAGCCAA
	**For qPCR**
OCT4 forward	TTGGGCTCGAGAAGGATGTGGT
OCT4 reverse	TGCATAGTCGCTGCTTGATCGC
NANOG forward	TGAACCTCAGCTACAAACAG
NANOG reverse	TGGTGGTAGGAAGAGTAAAG
PAX6 forward	ACCACACCGGTTTCCTCCTTCACA
PAX6 reverse	TTGCCATGGTGAAGCTGGGCAT
NESTIN forward	TTCCCTCAGCTTTCAGGACCCCAA
NESTIN reverse	AAGGCTGGCACAGGTGTCTCAA
SOX1 forward	GATCAGCAAGCGCCTGGGGG
SOX1 reverse	AGCAGCGTCTTGGTCTTGCGG
BRACHYURY forward	TGCTTCCCTGAGACCCAGTT
BRACHYURY reverse	GATCACTTCTTTCCTTTGCATCAAG
SOX17 forward	TGTCCCAAAACAGCTTCCTC
SOX17 reverse	TCACCCTTTTCGAGGATGAG
ACTB forward	TGAAGTGTGACGTGGACATC
ACTB reverse	GGAGGAGCAATGATCTTGAT
TUBB3 forward	ATTTCATCTTTGGTCAGAGTGGGGC
TUBB3 reverse	TGCAGGCAGTCGCAGTTTTCAC
DACH1 forward	CCGGTGGTGTGCAATGTGGAACAA
DACH1 reverse	GGTCTAGAACTTGCGTTGGTGCAGT
ZNF312 forward	GTGCGGCAAGGTGTTTAACGCT
ZNF312 reverse	ATTTTGTGCCTGCAGAGCGTGC
EN1 forward	AACCGCTACATCACGGAGCA
EN1 reverse	GATCTTGGCGCGCTTGTTCT
LMX1A forward	CAACTCAACAGAGGCGAGCATT
LMX1A reverse	GTTTTGGAACCACACCTGGAC
FOXA2 forward	CCATGCACTCGGCTTCCAG
FOXA2 reverse	TGTTGCTCACGGAGGAGTAG
	**For Sendai virus detection**
Sendai virus forward	AGACCCTAAGAGGACGAAGA
Sendai virus reverse	ACTCCCATGGCGTAACTCCATAGTG

Table S3

Supplemental Experimental Procedures

Isolation of human dermal fibroblasts (HDFs) and generation of aHDF-iPSCs

A skin-punch biopsy from a healthy 40-year-old Japanese male was used to generate aHDF-iPSCs (KA11, KA23, eKA3, and eKA4; passages 14-25). KA11 and KA23 were generated using retroviral vectors, and the other cell lines (eKA3 and eKA4) were generated using episomal plasmid vectors. aHDF-iPSCs from a PARK2 patient, ”PB” (PB2 and PB20; passages 8–17) were generated by retrovirus vectors, previously (Imaizumi et al., 2012). Maintenance of HDFs, lentiviral production, retroviral production, infection, episomal vector preparation, electroporation, stem cell culturing, and characterization were performed as described previously (Okita et al., 2011; Takahashi et al., 2007). All human primary cells were generated *in vitro* from tissue samples of human donors, with appropriate written informed consent given to the commercial providers. Skin biopsy and iPSC production procedures were approved by the Ethics Committee of Keio University School of Medicine (No. 20080016).

Isolation of human T-cells and generation of TiPSCs

Peripheral blood mononuclear cells (PBMCs) were obtained from two healthy donors, “KA” and “FK,” and one PARK2 patient, “PB,” by centrifuging heparinized blood over a Ficoll-Paque PREMIUM (GE Healthcare) gradient, according to the manufacturer’s instructions. CD3-positive cells were selected using a fluorescently conjugated anti-CD3 mAb (BD Pharmingen). PBMCs and cells subjected to fluorescence-activated cell sorting were cultured at 37°C in 5% CO_2 on a plate coated

with an anti-CD3 mAb in GT-T502 medium (KOHJIN BIO) containing 175 JRU/mL rIL-2. After 5 days of culture, activated PBMCs and activated T-cells were transferred to a 6-well plate coated with an anti-CD3 mAb at a density of 1.5 x 10^6 cells/well and incubated for an additional 24 hours. Thereafter, a solution containing SeV vectors was added to the wells. SeV vectors were obtained from DNAVEC Corp. or the National Institute of Advanced Industrial Science and Technology (AIST). The former vectors carried *OCT4*, *SOX2*, *KLF4*, or *c-MYC* (CytoTune^{TM}-iPS Reprogramming Kit) (Fusaki et al., 2009), whereas the latter vector carried all four reprogramming factor (Nishimura et al., 2011). At 24 hours post-infection, the medium was replaced with fresh GT-T502 medium. At 48 hours post-infection, cells were collected and transferred to a 10-cm dish containing mitomycin C-inactivated SNL feeder cells at a density of 5 x 10^4–5 x 10^5 cells/dish. After an additional 24 hours, the medium was replaced with hiPSC medium, which was changed every other day until colonies were picked. The generated hiPSCs were maintained on mitomycin C-inactivate SNL feeder cells in hiPSC medium. The healthy donor TiPSC lines, eTKA4 and eTKA5 (passages 12-19), were generated from KA-T-cells by episomal plasmid vectors. TKA4(AIST), TKA9(AIST), TKA7(DNAVEC), and TKA14(DNAVEC) (passages 14-20) were generated from KA-T-cells using SeV vectors made at each institution. TFK7(DNAVEC) and TFK12(DNAVEC) (passages 10-16) were generated from KF-T-cells using DNAVEC-SeV vectors. The PARK2 patient TiPSC lines, TPB4(DNAVEC), TPB8(DNAVEC), TPB11(DNAVEC), and TPB27(DNAVEC) (passages 14-20) were generated using DNAVEC-SeV vectors.

Immunocytochemical analysis of hiPSCs and hiPSC-derived neurons

Cells were fixed in phosphate-buffered saline (PBS) containing 4% paraformaldehyde for 30 minutes at room temperature. Thereafter, cells were incubated with the primary antibodies described in Table S2, washed with PBS, and incubated with an Alexa Fluor 488-, Alexa Fluor 555-, or Alexa Fluor 647-conjugated secondary antibody (1:500, Invitrogen). Images were obtained using an a universal fluorescence microscope (Axioplan2; Carl Zeiss) or confocal laser scanning microscope (LSM700; Carl Zeiss).

TCR clonotype mapping

Capillary electrophoresis was performed of genomic PCR products spanning TCRβ regions (Sandberg et al., 2005; van Dongen et al., 2003). TCRβ rearrangements were analyzed using BIOMED-2 protocols and a commercial kit (In VivoScribe Technologies) at Mitsubishi Chemical Medicine Corporation, Japan. The test comprises three multiplex master mixes that target conserved regions within the variable (V), diversity (D), and joining (J) regions.

Microarray analysis

RNA quality was assessed using a 2100 Bioanalyzer (Agilent Technologies). Total RNA (100 ng) was reverse-transcribed, labeled with biotin, and hybridized to a GeneChip® Human Genome U133 plus 2.0 Array (Affymetrix), which was subsequently washed and stained in a Fluidics Station 450 according to the manufacturer’s instructions.

The microarrays were scanned using a GeneChip Scanner 3000 7G, and the raw image files were converted into normalized signal intensity values using the MAS 5.0 algorithm.

Targets were selected that (a) were called “present” in at least one of the eight arrays analyzed, and (b) had a probe intensity of ≥50. In total, 19,383 targets were identified from this initial screen. The normalized logs were hierarchically clustered based on uncentered correlation with complete linkage using Cluster 3.0 (Eisen et al., 1998) and visualized using Java TreeView (Saldanha, 2004). Principal component analysis (PCA) was performed by Spotfire DecisionSite 9.1.2 using normalized data. Genespring GX software (Agilent Technologies) was used for pairwise scatter plot analysis. A Venn diagram was constructed to visualize the 322 genes that were up- or downregulated in the aHDF-iPSC/TiPSC and iPSC (KA)/iPSC (PB) groups, and the 25 genes that were up- or downregulated in each of the NS groups (moderated t-test $P < 0.05$, fold change > 2.0). For the GO analysis, *p* values were calculated using Fisher’s exact test.

Methylated DNA sequencing

Genomic DNA (1–2 μg) was fragmented by sonication using a Shearing System M220 (Covaris) and enriched using a CpG MethylQuest DNA Isolation Kit (Millipore) following the manufacturer's protocol. The chromatin immunoprecipitation sequencing (ChIP-seq) sample obtained after DNA enrichment was subjected to library preparation using the Illumina TruSeq™ ChIP Sample Prep Kit according to the manufacturer's protocol and quantified using a Hi-seq system. Obtained images were

analyzed and base-called using the BWA and SAM tools, and software was applied to align reads to the human genomic reference (hg19) at DNA CHIP RESERCH INC., Japan. Data were analyzed by Avadis NGS (Strand Scientific Intelligence).

Reverse-transcription-PCR

RNA isolation and reverse-transcription (RT)-PCR were performed as previously described (Okada et al., 2008). The amount of cDNA was normalized to the amount of β-actin mRNA. Quantitative RT-PCR (qPCR) was performed on an ABI PRISM Sequence detection System 7900HT (Applied BioSystems) using SYBR premix ExTaq Tli RNaseH Plus (Takara). The primers are described in Table S3.

PCR amplification of genomic DNA

Genomic DNA was purified from HDFs, T-cells, and hiPSCs using a DNeasy Kit (Qiagen). The PCR conditions were previously described (Table S3) (Kitada et al., 1998).

Lentiviral vector production

Lentiviral vector was produced as described previously (Zhou et al., 2014). 293T cells were transfected with the lentivirus plasmid, pCAG-HIVgp, and pCMV-VSV-G-RSV-Rev (Miyoshi et al., 1998) (kindly provided by Dr. Hiroyuki Miyoshi, RIKEN BRC, Japan). After 16–20 hours, the media was replaced and cells were incubated for a further 48–72 hours. The virus-containing media were collected and 0.45 μm filtered

followed by ultracentrifugation. The concentrated virus was suspended in PBS and stored at −80°C until use.

Electrophysiological analysis

For electrophysiological experiments, the culture medium was replaced with a physiological solution (118 mM NaCl, 2.5 mM KCl, 26 mM $NaHCO_3$, 1 mM NaH_2PO_4, 10 mM glucose, 4 mM $MgCl_2$, and 4 mM $CaCl_2$). Tetrodotoxin (TTX, 1 μM) was bath-applied. The electrodes (5-8 MΩ) were filled with whole-cell pipette solution (120 mM potassium acetate, 20 mM KCl, 0.1 mM $CaCl_2$, 5 mM $MgCl_2$, 0.2 mM EGTA, 5 mM ATP, and 10 mM HEPES, pH 7.3). The whole-cell recording of GFP-expressing neurons was configured using an EPC-7 amplifier (HEKA) and a Digidata 1200 acquisition board (Axon Instruments). The membrane potential was clamped at -60 mV. Membrane resistance (*R*m), series resistance (*R*s), and membrane capacitance (*C*m) were monitored. Only recordings with *R*m > 100 MΩ and *R*s < 20 MΩ were included in the analysis.

To obtain evoked synaptic currents from hiPSC-derived neurons, concentric tungsten stimulating electrodes were placed ~100 μm from the neuron being recorded. Synaptic AMPA receptor-mediated responses at -60 mV were averaged over 30 trials(Tada et al., 2013; Tada et al., 2010).

Carbonyl cyanide m-chlorophenyl hydrazone treatment

Neurons were cultured with 30 μM Carbonyl cyanide m-chlorophenyl hydrazone (CCCP) (Sigma-Aldrich) or DMSO for 48 hours. Thereafter, cells were fixed,

stained for βIII-tubulin and Complex-III Core I, and counterstained with Hoechst. To quantify the inner mitochondrial membrane (IMM) area of neurons, the cytoplasmic area was extracted as shown in Figure 6F. Complex-III Core I-positive signals within the extracted area were converted to grayscale and digitized. The IMM area was quantified from the digitized values using ImageJ software.

Oxidative stress analysis

The ROS levels were determined by measuring the CellROX fluorescence using the CellROX® Green Reagent for oxidative stress detection (Life Technologies). Briefly, neurons were incubated with the CellROX® Reagent for 30 min at 37°C, after which they were washed with PBS and then fixed by 4% PFA for 30 minutes at room temperature. Thereafter, the cells were incubated with the primary antibody, MAP2 (1:1000, Sigma), overnight at 4°C, washed with PBS, and incubated with an Alexa Fluor 555-conjugated secondary antibody (1:500, Invitrogen) for 1 hr at room temperature. The fluorescence in the MAP2-positive neurons was measured by an IN Cell Analyzer 6000 (GE Healthcare Biosciences).

Supplimental Reference

Eisen, M.B., Spellman, P.T., Brown, P.O., and Botstein, D. (1998). Cluster analysis and display of genome-wide expression patterns. Proc Natl Acad Sci U S A *95*, 14863-14868.

Kitada, T., Asakawa, S., Hattori, N., Matsumine, H., Yamamura, Y., Minoshima, S., Yokochi, M., Mizuno, Y., and Shimizu, N. (1998). Mutations in the parkin gene cause autosomal recessive juvenile parkinsonism. Nature *392*, 605-608.

Miyoshi, H., Blomer, U., Takahashi, M., Gage, F.H., and Verma, I.M. (1998). Development of a self-inactivating lentivirus vector. J Virol *72*, 8150-8157.

Development of defective and persistent Sendai virus vector: a unique gene delivery/expression system ideal for cell reprogramming. J Biol Chem *286*, 4760-4771.

Okita, K., Matsumura, Y., Sato, Y., Okada, A., Morizane, A., Okamoto, S., Hong, H., Nakagawa, M., Tanabe, K., Tezuka, K., et al. (2011). A more efficient method to generate integration-free human iPS cells. Nat Methods *8*, 409-412.

Saldanha, A.J. (2004). Java Treeview--extensible visualization of microarray data. Bioinformatics *20*, 3246-3248.

Sandberg, Y., van Gastel-Mol, E.J., Verhaaf, B., Lam, K.H., van Dongen, J.J., and Langerak, A.W. (2005). BIOMED-2 multiplex immunoglobulin/T-cell receptor polymerase chain reaction protocols can reliably replace Southern blot analysis in routine clonality diagnostics. J Mol Diagn *7*, 495-503.

Tada, H., Kuroki, Y., Funabashi, T., Kamiya, Y., Goto, T., Suyama, K., Sano, A., Mitsushima, D., Etgen, A.M., and Takahashi, T. (2013). Phasic synaptic incorporation of GluR2-lacking AMPA receptors at gonadotropin-releasing hormone neurons is involved in the generation of the luteinizing hormone surge in female rats. Neuroscience *248*, 664-669.

Tada, H., Okano, H.J., Takagi, H., Shibata, S., Yao, I., Matsumoto, M., Saiga, T., Nakayama, K.I., Kashima, H., Takahashi, T., et al. (2010). Fbxo45, a novel ubiquitin ligase, regulates synaptic activity. The Journal of biological chemistry *285*, 3840-3849.

van Dongen, J.J., Langerak, A.W., Bruggemann, M., Evans, P.A., Hummel, M., Lavender, F.L., Delabesse, E., Davi, F., Schuuring, E., Garcia-Sanz, R., et al. (2003). Design and standardization of PCR primers and protocols for detection of clonal immunoglobulin and T-cell receptor gene recombinations in suspect lymphoproliferations: report of the BIOMED-2 Concerted Action BMH4-CT98-3936. Leukemia *17*, 2257-2317.
