# Supplementary material for: Functional Neurons Generated from T Cell-Derived Induced Pluripotent Stem Cells for Neurological Disease Modeling
Source: Stem Cell Reports. 2016 Feb 18;6(3):422–35. doi: 10.1016/j.stemcr.2016.01.010 (PMC4788773; doi:10.1016/j.stemcr.2016.01.010)
Supplement: Document S2. Article plus Supplemental Information [file mmc2.pdf]

Stem Cell Reports
Resource
OPEN ACCESS

Functional Neurons Generated from T Cell-Derived Induced Pluripotent Stem Cells for Neurological Disease Modeling

Takuya Matsumoto,[1,2,13] Koki Fujimori,[1,13] Tomoko Andoh-Noda,[1,3] Takayuki Ando,[1] Naoko Kuzumaki,[1,4] Manabu Toyoshima,[5] Hirobumi Tada,[6] Kent Imaizumi,[1] Mitsuru Ishikawa,[1] Ryo Yamaguchi,[1,7] Miho Isoda,[1,7] Zhi Zhou,[1] Shigeto Sato,[8] Tetsuro Kobayashi,[9] Manami Ohtaka,[10] Ken Nishimura,[10,11] Hiroshi Kurosawa,[3] Takeo Yoshikawa,[5] Takuya Takahashi,[6] Mahito Nakanishi,[10] Manabu Ohyama,[9,14] Nobutaka Hattori,[8] Wado Akamatsu,[1,12,*] and Hideyuki Okano[1,*]

[1]Department of Physiology, Keio University School of Medicine, Shinjuku-ku, Tokyo 160-8582, Japan
[2]Institute for Innovation, Ajinomoto Co., Inc., Kawasaki-ku, Kanagawa 210-8681, Japan
[3]Division of Medicine and Engineering Science, University of Yamanashi, Interdisciplinary Graduate School of Medicine and Engineering, Kofu, Yamanashi 400-8511, Japan
[4]Department of Pharmacology, Hoshi University, Pharmacy and Pharmaceutical Sciences, Shinagawa-ku, Tokyo 142-8501, Japan
[5]Laboratory for Molecular Psychiatry, RIKEN Brain Science Institute, Wako, Saitama 351-0198, Japan
[6]Department of Physiology, Yokohama City University Graduate School of Medicine, Kanazawa-ku, Kanagawa 236-0027, Japan
[7]Regenerative & Cellular Medicine Office, Sumitomo Dainippon Pharma Co., Ltd, Chuo-ku, Kobe 650-0047, Japan
[8]Department of Neurology, Juntendo University School of Medicine, Bunkyo-ku, Tokyo 113-8431, Japan
[9]Department of Dermatology, Keio University, School of Medicine, Shinjuku-ku, Tokyo 160-8582, Japan
[10]Biotechnology Research Institute for Drug Discovery, National Institute of Advanced Industrial Science and Technology (AIST), Tsukuba, Ibaraki 305-8565, Japan
[11]Laboratory of Gene Regulation, University of Tsukuba, Faculty of Medicine, Tsukuba, Ibaraki 305-8575, Japan
[12]Center for Genomic and Regenerative Medicine, Juntendo University, School of Medicine, Bunkyo-ku, Tokyo 113-8431, Japan
[13]Co-first author
[14]Present address: Department of Dermatology, Kyorin University School of Medicine, Mitaka, Tokyo 181-8681, Japan
*Correspondence: awado@juntendo.ac.jp (W.A.), hidokano@a2.keio.jp (H.O.)
http://dx.doi.org/10.1016/j.stemcr.2016.01.010
This is an open access article under the CC BY license (http://creativecommons.org/licenses/by/4.0/).

SUMMARY

Modeling of neurological diseases using induced pluripotent stem cells (iPSCs) derived from the somatic cells of patients has provided a means of elucidating pathogenic mechanisms and performing drug screening. T cells are an ideal source of patient-specific iPSCs because they can be easily obtained from samples. Recent studies indicated that iPSCs retain an epigenetic memory relating to their cell of origin that restricts their differentiation potential. The classical method of differentiation via embryoid body formation was not suitable for T cell-derived iPSCs (TiPSCs). We developed a neurosphere-based robust differentiation protocol, which enabled TiPSCs to differentiate into functional neurons, despite differences in global gene expression between TiPSCs and adult human dermal fibroblast-derived iPSCs. Furthermore, neurons derived from TiPSCs generated from a juvenile patient with Parkinson's disease exhibited several Parkinson's disease phenotypes. Therefore, we conclude that TiPSCs are a useful tool for modeling neurological diseases.

INTRODUCTION

Neurological diseases have mainly been studied using animal models and immortalized neural cell lines due to the difficulties associated with examining the CNS of patients. Recent advances in human induced pluripotent stem cell (hiPSC) technologies have enabled neurological diseases to be modeled by culturing patient-specific neural cells in dishes (Imaizumi and Okano, 2014; Marchetto and Gage, 2012). The first hiPSCs were generated from cultured dermal fibroblasts by inducing reprogramming factors (Takahashi et al., 2007). hiPSCs derived from fibroblasts have been recognized as the standard iPSCs for several years. Therefore, most previously reported patient-specific hiPSC lines were generated from skin fibroblasts (Brennand et al., 2011; Imaizumi et al., 2012). Skin biopsies of patients are required to generate dermal fibroblast lines, and this can cause bleeding, infection, and scarring. Therefore, patient-specific hiPSCs should ideally be generated using less invasive procedures, but the resulting cells must have a similar pluripotency as dermal fibroblast-derived hiPSCs.

Yamanaka and colleagues first reported that iPSCs can be generated from various types of somatic cells, including hepatocytes (Aoi et al., 2008). Since then, several groups have generated hiPSCs from peripheral blood nuclear cells (PBMC) (Loh et al., 2010; Mack et al., 2011; Seki et al., 2010), which can be easily obtained from patients using minimally invasive methods. Among these reports, Fukuda and colleagues showed that a small number of CD3-positive T cells can be efficiently reprogrammed into iPSCs using Sendai virus (SeV) vectors (Seki et al., 2010). CD3-positive T cells can be cultured in vitro using plates coated with an anti-CD3 monoclonal antibody (mAb) and in the presence of recombinant interleukin-2 (rIL-2). These cells

can be stored in frozen vials and thawed several months later. Thus, CD3-positive T cells can be obtained non-invasively, are easily stored and efficiently reprogrammed, and might therefore be an ideal source of patient-specific iPSCs.

We sought to determine whether T cell-derived iPSCs (TiPSCs) could be used to analyze neurological diseases. Several issues regarding the utilization of TiPSCs in neurological studies remain unresolved. First, previous studies indicated that each iPSC clone retains an epigenetic memory relating to the cell type from which they are derived, even after their re-differentiation into somatic cells, and this restricts their differentiation potential (Kim et al., 2010, 2011; Panopoulos et al., 2012; Polo et al., 2010). Kim et al. reported that there are distinct differences in the genome-wide DNA methylation profiles of iPSCs derived from cord blood cells (CB-iPSCs) and iPSCs derived from neonate keratinocytes (K-iPSCs), and that these differences are closely related to their differentiation potentials. K-iPSCs had an enhanced potential to differentiate into keratinocytes in comparison with CB-iPSCs, even though both types of iPSCs were established from the same donor. Second, rearrangement of T cell receptor (TCR) chain genes in mature T cells indicates that they are not identical to naive lymphocytes at the genomic level. Although such rearrangements are reportedly retained in TiPSCs (Seki et al., 2010), it is unknown whether they affect the neural differentiation and function of TiPSCs.

In the present study, we showed that TiPSCs have a reduced tendency to differentiate into the neural lineage via embryoid body (EB) formation in comparison with adult human dermal fibroblast-derived iPSCs (aHDF-iPSCs). To overcome this, we established a neurosphere-based robust differentiation protocol that uses a low density of cells and hypoxic conditions. Using this method, TiPSCs efficiently and stably differentiated into mature functional neurons, similar to aHDF-iPSCs.

Furthermore, we demonstrated that TiPSC-derived neurons could be used as a Parkinson's disease model.

RESULTS

Generation of Genetically Matched hiPSCs from T Cells and Skin Fibroblasts

To compare TiPSCs and aHDF-iPSCs in a similar genetic background (i.e., rearrangements of TCR chain genes), we generated these cells from T cells and dermal fibroblasts isolated from a healthy donor. TiPSCs (eTKA4, eTKA5, TKA7 [DNAVEC], TKA14 [DNAVEC], TKA4 [AIST], and TKA9 [AIST]) were generated from CD3-positive lymphocytes using episomal plasmid vectors (containing *OCT4*, *SOX2*, *KLF4*, *L-MYC*, *LIN28*, *EBNA1*, and sh*p53* or dominant-negative *p53*) (Okita et al., 2013) or SeV vectors, which were produced by DNAVEC Corp. (Cytotune) or the National Institute of Advanced Industrial Sciences and Technology (AIST). The DNAVEC SeV vector carried *OCT4*, *SOX2*, *KLF4*, or *c-MYC* on each of four vectors (Fusaki et al., 2009), whereas the AIST SeV vector carried all four reprogramming factors on a single vector (Nishimura et al., 2011). aHDF-iPSCs (KA11, KA23, eKA3, and eKA4) were also generated from the same healthy donor using retroviruses (*OCT4*, *SOX2*, *KLF4*, and *c-MYC*) or episomal plasmid vectors (Okita et al., 2013).

Immunocytochemical analysis revealed that all TiPSC clones expressed the pluripotency markers TRA-1-60 and stage-specific embryonic antigen 4 (SSEA4) (Figure 1A) at levels comparable with those in aHDF-iPSC clones. Expression of *OCT4* and *NANOG* was quantified by qPCR (Figures 1B and 1C). Transgenes used for reprogramming were not detected by RT-PCR (Figure S1A). The genomic structure did not significantly differ between TiPSCs and aHDF-iPSCs according to a comparative genomic hybridization array (Figure S1B). We next examined TCRβ rearrangements in genomic DNA of TiPSC clones to confirm that they were reprogrammed from CD3-positive T cells (Figure S1C). We amplified and analyzed TCRβ genomic regions by PCR and capillary electrophoresis. aHDF-iPSC clones (KA11 and PB2) did not show a specific positive peak indicative of TCRβ rearrangement in the Vβ/Jβ1,2, Vβ/Jβ2, or Dβ/Jβ region, whereas TiPSC clones (TKA4 [AIST], TKA9 [AIST], TPB4 [DNAVEC], and TPB8 [DNAVEC]) showed at least one specific peak in these regions. These results suggest that TiPSC clones were derived from CD3-positive T cells with TCRβ rearrangement.

Comparison of the Biological Characteristics of TiPSCs and aHDF-iPSCs

To determine whether the origin of hiPSCs affects their global gene expression, we evaluated the global transcriptional profiles of TiPSCs and aHDF-iPSCs that were passaged fewer than 10–15 times using microarray analysis. Data, excluding genes whose expression values were low in all samples, were normalized and subjected to PCA and hierarchical clustering (Figures 1D and 1E). The samples could be crudely divided into three categories: T cells, aHDF, and pluripotent stem cells (PSC) (including TiPSCs, aHDF-iPSCs, and ESCs). Hierarchical clustering placed TiPSCs and aHDF-iPSCs into different groups (Figure 1E). On the other hand, TiPSCs generated using SeV vectors from DNAVEC Corp. (Fusaki et al., 2009) and those generated using SeV vector from AIST (Fusaki et al., 2009; Nishimura et al., 2011) did not cluster separately, and neither did aHDF-iPSCs generated using retroviruses and those generated using episomal plasmid vectors. The genome methylation profiles of TiPSC and aHDF-iPSC clones were compared by ChIP-seq analysis. Methylated genomic

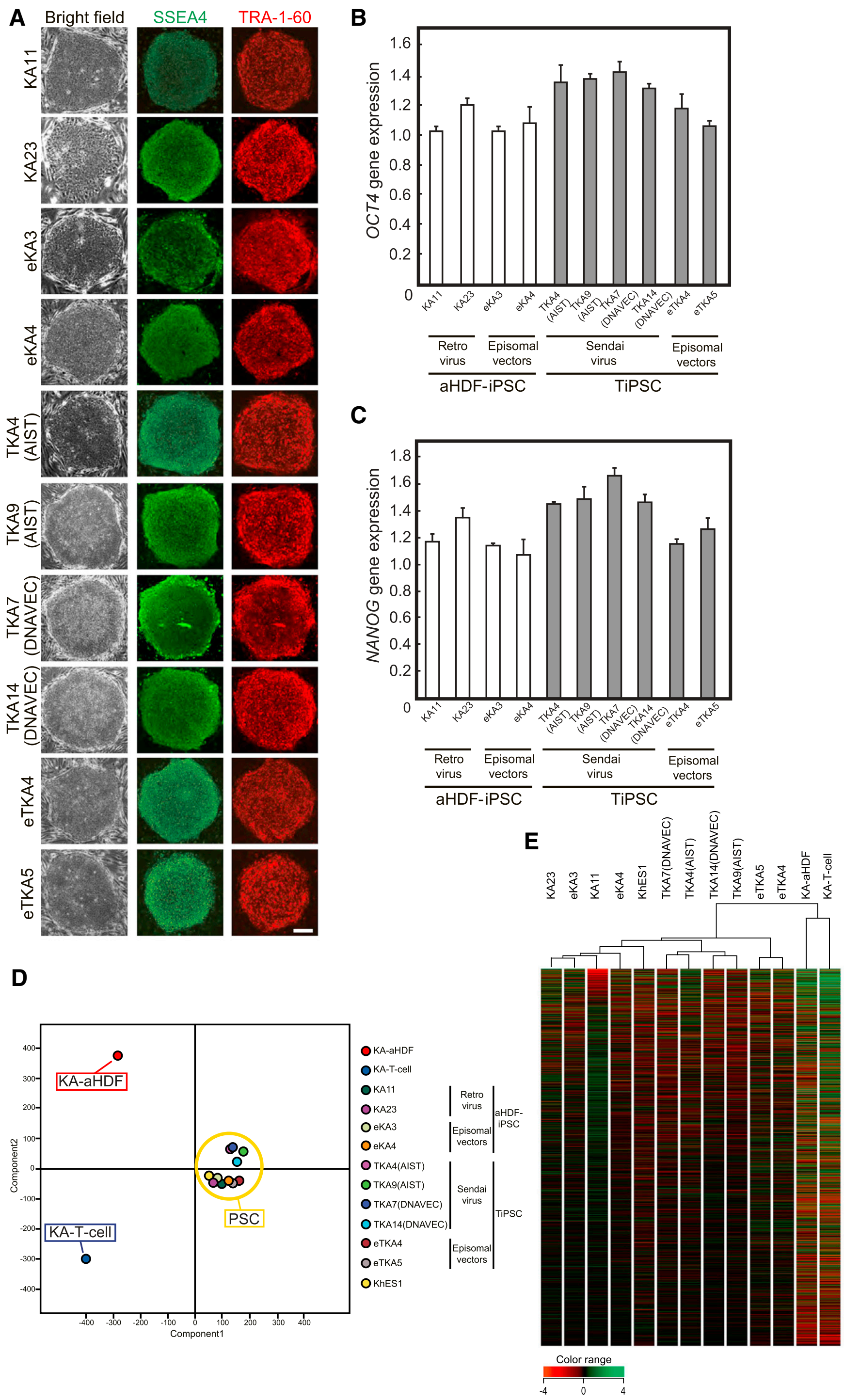

Figure 1. Characterization of iPSCs Derived from T Cells and Adult Human Dermal Fibroblasts

(A) All of the iPSCs derived from KA-T cells and KA-aHDF were immunopositive for the pluripotency markers SSEA4 (green) and TRA-1-60 (red).

(B and C) A comparison of the mRNA transcript levels in TiPSCs and aHDF-iPSCs by qPCR. The levels of endogenous *OCT4* (B) and endogenous *NANOG* (C) were comparable in the two types of iPSCs. The expression levels were normalized to the mean level in aHDF-iPSCs (set at 1.0) (n = 3 independent experiments; mean ± SD).

(D) A comparison of the global gene expression profiles of aHDF-iPSCs, TiPSCs, and the original cells (KA-aHDF and KA-T cells) derived from a healthy man. A principal component analysis of the gene expression data. Yellow, PSC; red, KA-aHDF; blue, KA-T cells.

(E) The results of a hierarchical clustering analysis of the global gene expression.

See also Figure S1.

DNA was precipitated using a recombinant methyl-binding domain-containing protein and sequenced using a next-generation sequencer. The data were subjected to hierarchical clustering (Figure S1D). Consistent with the expression profiles, this analysis placed TiPSCs and aHDF-iPSCs into different groups according to the cell type from which they were derived. In addition, we identified 39 genes that were upregulated in the TiPSCs relative to aHDF-iPSCs (fold change >5.0). A gene ontology analysis identified a number of immune response-associated terms, suggesting that T cell-specific genes were still activated in the TiPSCs (Table S1). These data suggest that the cell type of origin

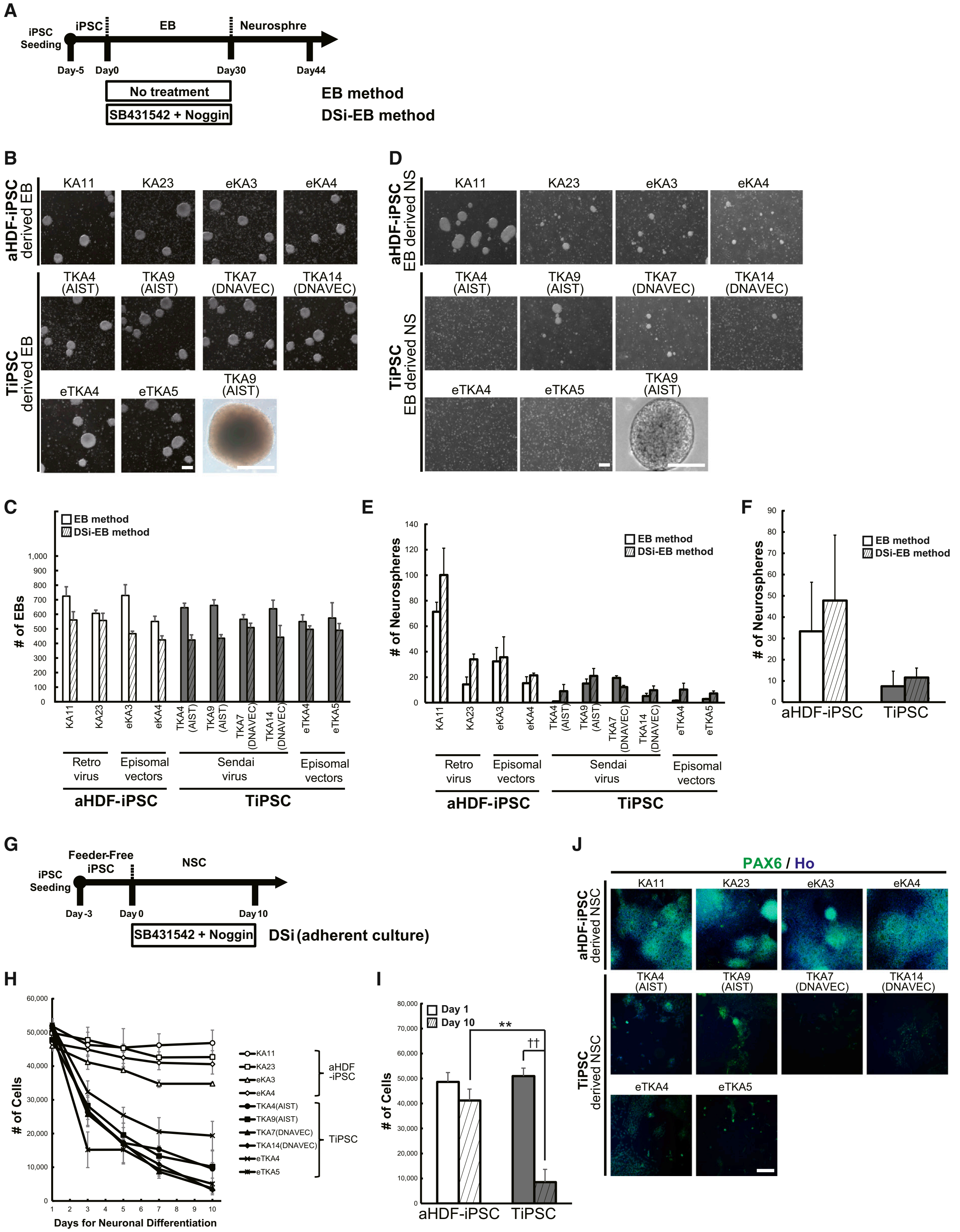

(legend on next page)

influences the properties of the hiPSCs generated from the same donor.

Compared with aHDF-iPSCs, the TiPSCs Were Poorly Differentiated into the Neural Lineage by an EB-Based Spontaneous Neural Differentiation Protocol

To confirm the impact of the original cell types on the iPSC differentiation, we evaluated whether TiPSCs could differentiate into neural stem/progenitor cells (NS/PCs) as efficiently as aHDF-iPSCs by using EB. In accordance with the previous studies using this method (Nori et al., 2011), we initially formed EBs from dissociated hiPSCs, and differentiated them into NS/PCs in a subsequent step (Figure 2A). When we cultured dissociated hiPSCs under the floating culture condition, all of the hiPSC lines converted EBs with a similar EB formation number (Figures 2B and 2C). We dissociated these EBs to expand NS/PCs in serum-free medium containing fibroblast growth factor 2 (FGF-2) and thereby generate neurospheres. All of the TiPSC lines we tested (TKA4 [AIST], TKA9 [AIST], TKA7 [DNAVEC], and TKA14 [DNAVEC]) formed few neurospheres, while all aHDF-iPSC lines were able to form neurospheres within 12 days (Figures 2D–2F).

We next tested a forced neural differentiation protocol using two inhibitors of SMAD signaling, SB431542 and Noggin, which were previously reported to induce dual SMAD inhibition (DSi) (Chambers et al., 2009) during EB formation. Although the EB method with DSi increased the average number of neurospheres formed compared with untreated control aHDF-iPSC (Figure 2F), the difference was not statistically significant because of the high level of variation among the iPSC lines. In contrast, the TiPSC-derived EBs formed very few neurospheres, even with DSi (Figures 2D–2F).

To assess the differences in the properties between aHDF-iPSC-EB and TiPSC-EB, we next quantified the expression levels of various genes in the neurospheres. qPCR analysis revealed that expression of the neural markers *PAX6*, *NESTIN*, *SOX1*, and *TUBB3* was much lower in TiPSC-derived EBs than in aHDF-iPSC-derived EBs (Figure S2B). However, the expression of mesendodermal (*BRACHYURY*) and endodermal (*SOX17*) markers was higher in TiPSC-derived EBs than in aHDF-iPSC-derived EBs (Figure S2C). DSi significantly upregulated the expression of *NESTIN* and *SOX1*, and slightly decreased the expression of *BRACHYURY* and *SOX17*, in aHDF-iPSC-derived EBs (Figures S2B and S2C). In contrast, the TiPSC-derived EBs were hardly affected by DSi, and the expression of neural markers remained much lower than that in the aHDF-iPSC-derived EBs (Figures S2B and S2C).

In an additional approach to neural differentiation, we cultured feeder-free TiPSCs under DSi conditions (Chambers et al., 2009). Dissociated iPSCs were plated on Matrigel-coated plates in complete conditioned media. After 3 days, further cultivation utilized knockout serum replacement media and/or N2 media containing 10 μM SB431542 and 200 ng/ml Noggin for 10 days (Figure 2G). We quantified the cell number during the differentiation period to assess the efficiency of neural differentiation. Although all aHDF-iPSC lines tested (KA11, KA23, eKA3, and eKA4) yielded a set number of cells during neural induction, all TiPSC lines (TKA4 [AIST], TKA9 [AIST], TKA7 [DNAVEC], TKA14 [DNAVEC], eTKA4 and eTKA5) showed a marked time-dependent decrease in cell number (Figures 2H and 2I). Immunocytochemical analysis revealed that both differentiated TiPSCs and aHDF-iPSCs expressed the neural marker PAX6, although the density of TiPSC-derived PAX6-positive cells was poor (Figure 2J).

We also tested whether the neural rosette method could induce NS/PCs from EBs (Koch et al., 2009). However, rosette-like cells derived from TiPSCs expressed few NS/PC markers (Figure S3). These data suggest that TiPSCs exhibit poor differentiation into neuro-ectoderm lineage compared with aHDF-iPSCs, even in the presence of dual SMAD inhibition.

Figure 2. TiPSCs Could Differentiate into Only a Few Neural Lineage Cells when Induced by the Classical-EB, Modified-EB, and Dual SMAD Inhibition Protocols

(A) An overview of the culture protocols for neurosphere induction via EB. EB formation with dual SMAD inhibition (DSi).
(B) Images of floating EBs derived from aHDF-iPSCs and TiPSCs. Scale bars, 200 μm.
(C) The number of EBs was determined by counting the spheres in which D_A > 50 μm (n = 4–6 independent experiments; mean ± SD). D_A denotes the diameter of the EB.
(D) Images of floating neurospheres derived from aHDF-iPSC- and TiPSC-EBs. Scale bars, 200 μm.
(E and F) The number of neurospheres was determined by counting the spheres in which D_A > 50 μm (n = 4–6 independent experiments; mean ± SD). D_A denotes the diameter of the neurospheres.
(G) An overview of the culture protocol for neural stem cell (NSC) induction using DSi under feeder-free conditions.
(H and I) The number of differentiating TiPSCs was significantly decreased compared with aHDF-iPSCs. **p < 0.01 when comparing aHDF-iPSCs and TiPSCs at day 10; ††p < 0.01 when comparing TiPSCs at day 1 and TiPSCs at day 10. The significance of the differences was assessed using Student's *t* test. At least three experiments were performed for each group.
(J) Differentiated TiPSCs and aHDF-iPSCs were immunoreactive for the neural marker PAX6.
See also Figures S2 and S3.

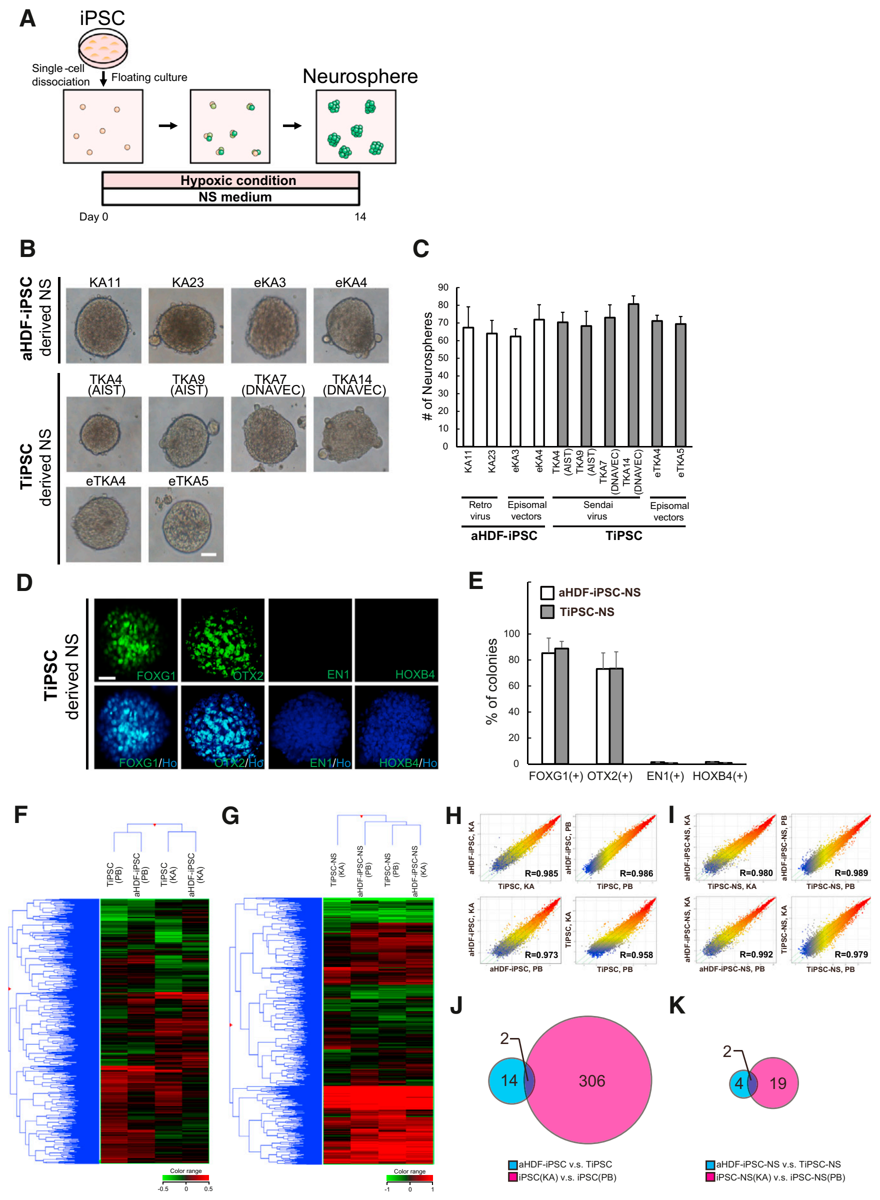

Figure 3. The Expression of Several Neuronal Markers Was Indistinguishable between Neurospheres Generated from TiPSCs and aHDF-iPSCs Using the Direct Neurosphere Converting Method

(A) A schematic representation of the direct neurosphere converting method (dNS method).

(B) Images of floating neurospheres derived from aHDF-iPSCs and TiPSCs obtained using the dNS method. Scale bars, 200 μm.

(C) The number of neurospheres was determined by counting the spheres in which $D_A > 50$ μm (n = 4–6 independent experiments; mean ± SD). D_A denotes the diameter of the neurospheres.

(D and E) The results of an immunocytochemical analysis of neurospheres for anteroposterior markers. (D) Representative images of anteroposterior marker-positive neurospheres. Scale bars, 200 μm. (E) The percentage of anteroposterior marker-positive neurosphere colonies. aHDF-iPSCs, KA11, KA23, and eKA3; TiPSCs, TKA4 (AIST), TKA7 (DNAVEC), and eTKA4 (n = 3 independent experiments; mean ± SD).

(F–I) Comparison of the global gene expression profiles of TiPSCs (TKA4 [AIST], TKA9 [AIST], TPB4 [DNAVEC], and TPB8 [DNAVEC]) and aHDF-iPSCs (KA23, eKA3, PB2, and PB20) and tertiary neurospheres derived from them. (F and G) Hierarchical clustering analysis of global gene expression. (H and I) Comparison of the global gene expression patterns.

(J and K) Venn diagram of human genes whose expression increased or decreased in the aHDF-iPSC/TiPSC, iPSC (KA)/iPSC (PB), and their NSs groups (moderated *t* test, $p < 0.05$, fold change >2.0).

See also Figures S4 and S5.

TiPSCs Can Be Differentiated into the Neural Lineage as Effectively as aHDF-iPSCs Using a Method with Direct Neurosphere Formation

TiPSCs preferentially differentiated into mesodermal and endodermal cells, not ectodermal cells, during EB formation (Figure S2). The absence of ectodermal cells in EBs reduced the formation of neurospheres from dissociated EBs. To overcome this differentiation preference of TiPSCs, we developed a method to induce neurons from iPSCs that does not involve EB formation (Figure 3A). It has been reported that low-density dissociation of mouse ES cells, in the absence of serum, resulted in most of the cells expressing neural markers (Tropepe et al., 2001). Therefore, we simply dissociated hiPSCs into single cells and cultured these cells in serum-free medium to induce ectodermal cells.

Dissociated hiPSCs are vulnerable to apoptosis. To avoid this, we added Y27632, which promotes the survival of single hiPSCs (Watanabe et al., 2007), and cultured cells in hypoxic conditions, which promotes the survival of NS/PCs by inhibiting apoptosis (Clarke and van der Kooy, 2009). As expected, these conditions enhanced the number of neurospheres formed (Figure S4). Neurospheres formed after 14 days of culture in medium containing FGF-2, Y27632, and human leukemia inhibitory factor (hLIF) under low-oxygen conditions (Figure 3A). We compared the

efficiency of neural induction between TiPSCs and aHDF-iPSCs using this direct neurosphere (dNS) method. The numbers of neurospheres induced from TiPSCs (TKA4 [AIST], TKA9 [AIST], TKA7 [DNAVEC], TKA14 [DNAVEC], eTKA4, and eTKA5) and aHDF-iPSCs (KA11, KA23, eKA3, and eKA4) did not significantly differ (Figures 3B and 3C).

To clarify the regional identity of the neurospheres formed by this dNS method, we next examined the expression of anteroposterior markers in hiPSC-derived neurospheres by an immunocytochemical analysis. Both the TiPSC- and aHDF-iPSC-derived neurospheres mostly expressed the forebrain marker, FOXG1 (Oliver et al., 1995), and the forebrain/midbrain marker, OTX2 (Simeone et al., 1992) (Figures 3D and 3E). However, only a few of these neurospheres expressed EN1, which is expressed in the mesencephalon and the metencephalon (Hanks et al., 1995), or HOXB4, a marker of the myelencephalon and the spinal cord (Hunt et al., 1991) (Figures 3D and 3E). These results indicated that both the TiPSC- and aHDF-iPSC-derived neurospheres were similarly differentiated into the anterior region around the forebrain/midbrain by this dNS method.

To confirm the robustness of the dNS method in the neural differentiation of TiPSCs, we generated TiPSCs from two additional donors (FK and PB); FK cells derived from another healthy donor, and PB cells derived from a patient with a PARK2 mutation, which is known to cause juvenile Parkinson's disease (PARK2). We had previously established fibroblast-derived hiPSCs from this patient (Imaizumi et al., 2012). Using the dNS method, the numbers of neurospheres were not significantly decreased and differentiated similarly even if they were generated from the various TiPSC clones (TFK7 [DNAVEC], TFK12 [DNAVEC], TPB4 [DNAVEC], TPB8 [DNAVEC], TPB11 [DNAVEC], and TPB27 [DNAVEC]) (Figure S5).

iPSCs favor differentiation along the lineage of the cell type from which they are derived (Kim et al., 2010, 2011; Polo et al., 2010). However, Yamanaka and colleagues reported that hepatic differentiation of hiPSCs is largely influenced by differences in the donor, rather than by the cell type from which they are derived (Kajiwara et al., 2012). We sought to determine how donor differences and the original cell type influence the neural differentiation of hiPSCs. The global transcriptional profiles of iPSCs and iPSC-derived neurospheres generated from T cells or fibroblasts of the healthy donor and PARK2 patient (KA23, eKA3, TKA4 [AIST], TKA9 [AIST], PB2, PB20, TPB4 [DNAVEC], and TPB8 [DNAVEC]) were examined. The gene expression analysis of iPSCs showed that there were differences in their properties associated with the original cell types and donors, while the analysis of NSs obtained using the dNS method demonstrated that these differences among iPSCs, especially those caused by different genetic backgrounds, were smaller (Figures 3F–3I). In addition, as shown in the Venn diagrams in Figures 3J and 3K, which classified the cells based on the up- or downregulated genes in comparison with aHDF-iPSC/TiPSC, iPSC (KA)/iPSC (PB), and their NSs, the number of altered genes was also much lower in the NSs than that in the iPSCs (aHDF-iPSC versus TiPSC, 16 genes; iPSC(KA) versus iPSC(PB), 308 genes; aHDF-iPSC-NS versus TiPSC-NS, six genes; iPSC-NS(KA) versus iPSC-NS(PB), 21 genes; moderated t test, $p < 0.05$, fold change >2.0). These analyses indicate that NS induction by the dNS method decreases the variations in the iPSC properties caused by the differences in the original cell types and donors. Furthermore, we included six additional clones and quantified the expression of pluripotency (*OCT4* and *NANOG*) and NS/PC (*PAX6* and *SOX1*) markers by qPCR (Figures 4A–4D). The expression of *OCT4* was lower in neurospheres derived from PB clones than in neurospheres derived from the other donors' clones (Figures 4A and 4I). However, there was no difference in the expression of *OCT4* between the cells based on the cell origins or the methods used for iPSC derivation (Figures 4E and 4M). Furthermore, the expression of *NANOG* and NS/PC markers did not significantly differ among the various neurospheres, regardless of the original cell types, donors, or methods used to generate iPSCs (Figures 4B–4D, 4F–4H, 4J–4L, and 4N–4P). These results indicate that the dNS method could lead to the formation of neurospheres from hiPSCs, independent of the cell types of origin, donors, and iPSC generation methods, and could minimize the differences in the differentiation propensities of each hiPSC line.

TiPSCs Can Be Differentiated into Functional Neurons and Cells of Various Neuronal Subtypes to a Similar Extent as aHDF-iPSCs Using the dNS Method

We next examined whether NS/PCs derived from TiPSCs could differentiate into functional neuronal subtypes as efficiently as NS/PCs derived from aHDF-iPSCs. Neurospheres were plated on fibronectin- and poly-L-ornithine (PO)-coated plates and differentiated in media hormone mix (MHM) containing B27, 10 ng/ml brain-derived neurotrophic factor (BDNF), 10 ng/ml glial cell-derived neurotrophic factor (GDNF), 200 μM ascorbic acid, and 1 mM dibutyryl-cAMP for 30–70 days. After 60 days, the differentiated cells expressed the neural marker, microtubule-associated protein 2 (MAP2), another neural marker, βIII-tubulin (Tuj1), and an astrocyte marker, glial fibrillary acidic protein (GFAP), with similar differentiation ratios observed between the TiPSCs and aHDF-iPSCs, indicating that they had equally differentiated into neurons and astrocytes (Figures 5A–5G). An immunocytochemical analysis revealed that these MAP2-positive neurons included tyrosine hydroxylase (TH)-positive dopaminergic neurons

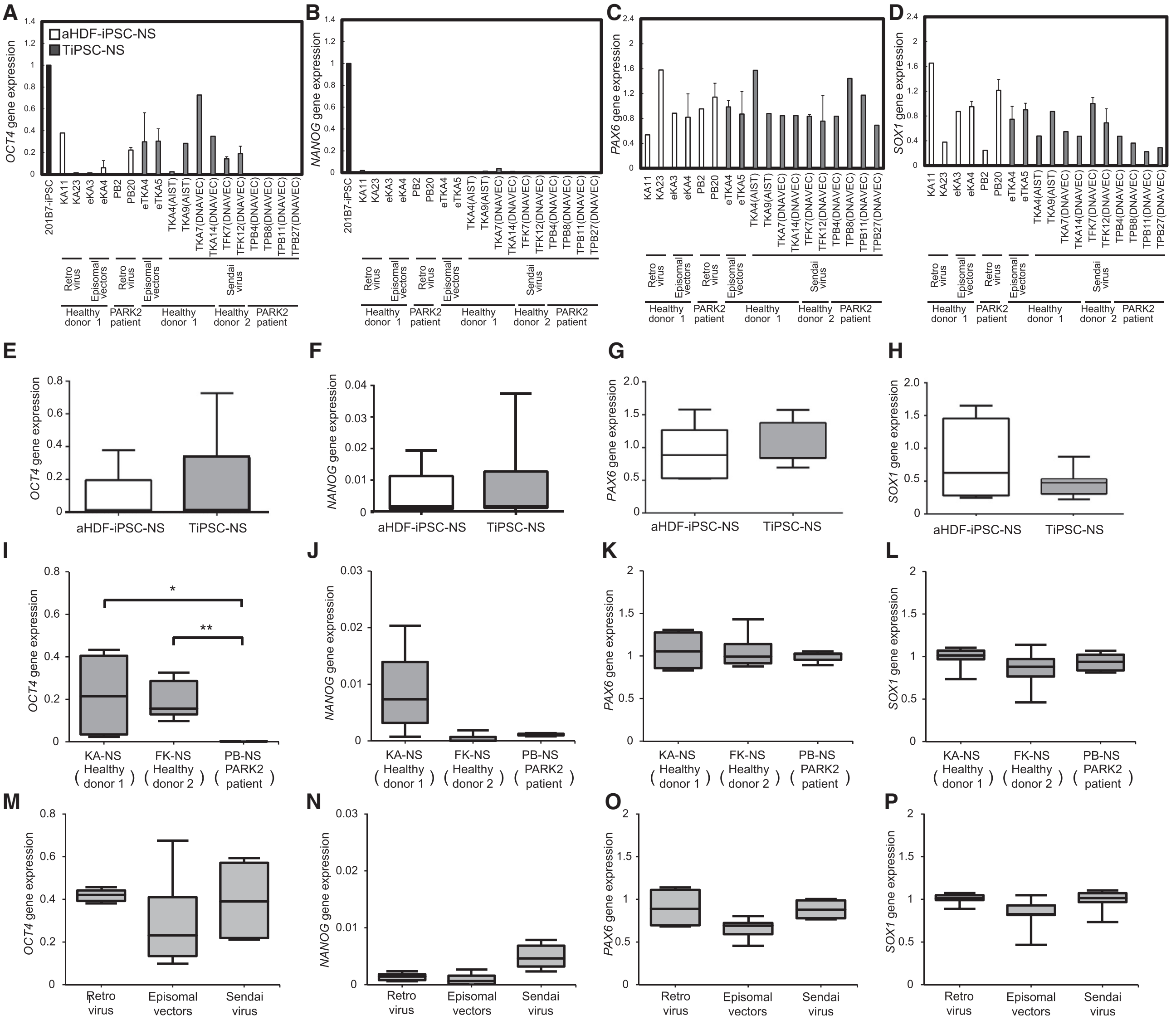

Figure 4. Comparison of mRNA Transcript Levels among Neurospheres Generated from the Various Types of Induced Pluripotent Stem Cells by Quantitative Reverse-Transcription PCR

(A–D) Levels of pluripotent markers (*OCT4* and *NANOG*) and neural stem markers (*PAX6* and *SOX1*) in tertiary neurospheres generated from each iPSC clone. Expression levels were normalized to the mean level of each gene in 201B7-iPSCs, which was a previously established human iPSC clone (Takahashi et al., 2007) (*OCT4* and *NANOG*), or neural stem/progenitor cells derived from aHDF-iPSCs (*PAX6* and *SOX1*) (set at 1.0) (n = 3 independent experiments; mean ± SD).

(E–H) Statistical analysis of mRNA transcript levels in tertiary neurospheres derived from aHDF-iPSCs and TiPSCs. Expression of pluripotency and neural stem markers was similar in these two types of cells.

(I–L) The results of a statistical analysis of the mRNA transcript levels in tertiary neurospheres derived from KA, FK, and PB clones. The *OCT4* expression was lower in neurospheres derived from the PB clones than in neurospheres derived from the other donors' clones. $^{**}p < 0.01$ and $^{*}p < 0.05$ compared with the control. The significance of differences was assessed using Tukey's test.

(M–P) The results of a statistical analysis of the mRNA transcript levels in tertiary neurospheres derived from hiPSCs generated using different methods to transduce the Yamanaka factors: Retroviruses, Episomal vectors, and Sendai virus. The expression levels of markers of pluripotency and neural stems were similar among the three types of cells tested.

(Figures 5H–5J), γ-aminobutyric acid (GABA)-positive GABAergic neurons (Figures 5K–5M), and vesicular glutamate transporter 1-positive glutamatergic neurons that expressed synaptophysin (Figures 5N–5P). Synaptophysin had a punctate distribution, indicative of synaptic formation in vitro.

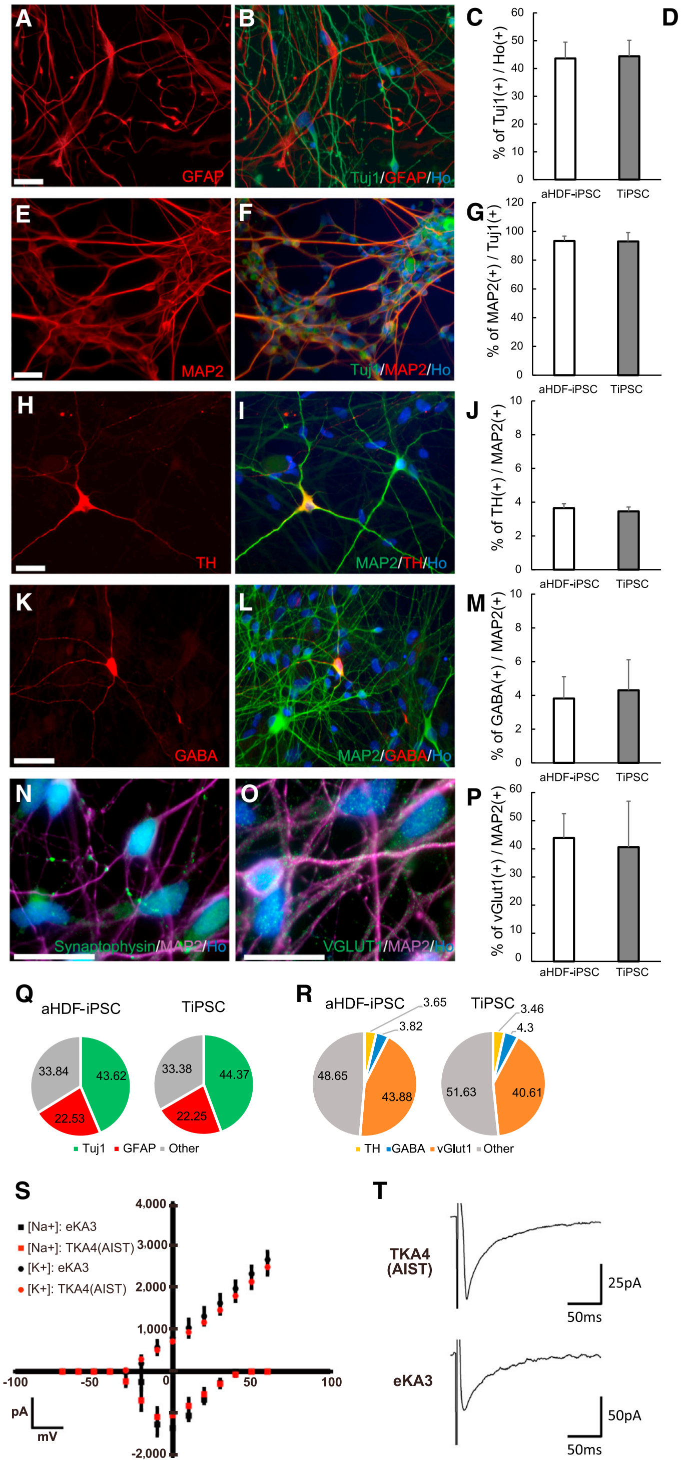

Figure 5. Neurospheres Generated from TiPSCs Have the Potential to Differentiate into Specific Neuronal Subtypes and Could Be Differentiated into Electrophysiologically Functional Neurons Using the dNS Method

(A–P) Differentiation potential was assessed in media hormone mix containing B27 for 30–60 days. Neural cells derived from TiPSCs (TKA4 [AIST] and TKA7 [DNAVEC]) were immunoreactive for MAP2, βIII-tubulin (Tuj1), GFAP, TH, GABA, VGLUT1, and synaptophysin. Scale bars, 50 μm (n = 5 independent experiments; mean ± SD).

(Q and R) A summary of the ratio of cell types and neuronal subtypes differentiated from aHDF-iPSCs and TiPSCs.

(S) Current-voltage plot of sodium and potassium currents of neurons derived from TKA4 [AIST] and eKA3 clones.

(T) Voltage-clamp recordings of neurons derived from TKA4 [AIST] and eKA3 clones showing evoked synaptic currents.

See also Figure S6.

Figures 5Q and 5R show summaries of the data for these differentiated cell types and neuronal subtypes, which suggested that the TiPSCs could differentiate into various kinds of cells as efficiently as aHDF-iPSCs. The electrophysiological properties of the TiPSC-derived neurons were examined to confirm that they were functional. We recorded voltage-sensitive currents in 30- to 60-day-old TiPSC- and aHDF-iPSC-derived neurons. iPSC-derived neurospheres were infected with a lentivirus expressing human synapsin promoter-driven GFP (CSIV–hSynI-GFP-IRES2-NeoR) (Zhou

et al., 2014) and differentiated into neurons. TTX-sensitive voltage-gated membrane currents were indistinguishable between neurons derived from TiPSCs and those derived from aHDF-iPSCs (Figure S6A). Na^+ and K^+ currents also had similar current-voltage relationships in TiPSC-derived neurons and aHDF-iPSC-derived neurons (Figure 5S). Current-clamp measurements showed that these two types of neurons both generated multiple action potentials (Figure S6B). We next investigated whether TiPSC-derived neurons could form synapses. Evoked postsynaptic currents were observed in whole-cell voltage-clamp recordings of TiPSC-derived neurons (Figure 5T). These results show that the dNS method can differentiate TiPSCs into functional neurons, and that the electrophysiological function of these neurons is indistinguishable from that of aHDF-iPSC-derived neurons.

Neurons Derived from TiPSCs Established from a PARK2 Patient Exhibited Several Different Parkinson's Disease Phenotypes

To determine whether hiPSCs established from the peripheral blood of patients can be used as a model of neurological diseases, we differentiated TiPSCs derived from a patient with PARK2, a familial form of Parkinson's disease, into neurons. A consistent neurochemical abnormality found in Parkinson's disease is the degeneration of dopaminergic neurons in the substantia nigra. Therefore, we first modified the dNS method to generate midbrain dopaminergic neuron (mDAN)-enriched culture by treating the cells with several small molecules, FGF-8, sonic hedgehog (Shh), purmorphamine (PMA), and CHIR99021 (CHIR) during the NS formation, for a total of 42 days (Figure 6A). qPCR showed that there was upregulation of markers of mDAN differentiation, including *EN1*, *LMX1A*, and *FOXA2* (Figure 6B). After they were allowed to mature for 13 days, immunostaining of the neural epithelial cells demonstrated that nearly 20% of the MAP2-positive cells expressed the dopaminergic neuron marker, TH (Figures 6C and 6D). These data related to the expression pattern marked the acquired TH-positive neurons as mDANs (Figures 6B–6D).

The PARK2 patient-derived TiPSCs (TPB4 [DNAVEC], TPB8 [DNAVEC], TPB11 [DNAVEC], and TPB27 [DNAVEC]) had a homozygous deletion in exons 6 and 7 of the *PARK2* gene (Figure 6E), expressed the human pluripotent markers TRA-1-60 and SSEA4 (Figure S5), and could differentiate into dopaminergic neurons (Figure S5). Parkin is a component of an E3 ubiquitin ligase involved in mitochondrial homeostasis (Shimura et al., 2000). We previously reported that the hiPSCs established from fibroblasts of a PARK2 patient (the same patient used as the source of TiPSCs in the current study) exhibited an abnormal turnover of damaged mitochondria (Imaizumi et al., 2012) and increased reactive oxygen species (ROS) production in the neurons. Therefore, we first treated TiPSC-derived neurons with carbonyl cyanide *m*-chlorophenyl hydrazone (CCCP), which triggers the loss of mitochondrial membrane potential and results in the removal of damaged mitochondria. To determine the extent to which damaged mitochondria were eliminated after CCCP treatment, we visualized the area of the inner mitochondrial membrane (IMM) using an antibody against the IMM marker Complex-III core I. Compared with untreated samples, CCCP treatment elicited a dramatic decrease in the IMM area in control neurons (TKA clones) but not in PARK2 neurons (TPB clones) (Figures 6F and 6G).

Using the same neuronal samples treated with CCCP, we next stained the cells for TH and quantified the ratio of TH-positive neurons to evaluate the vulnerability of dopaminergic neurons to mitochondrial stress. We observed that there was a decrease in the number of TH-positive neurons in PARK2 neuronal cultures (TPB clones) due to the treatment with CCCP (Figure 6H), indicating that the dopaminergic neurons derived from PARK2-iPSCs were more vulnerable to mitochondrial stress compared with control-iPSCs.

Finally, we evaluated the ROS production in the neurons derived from control- and PARK2-TiPSCs using the CellROX Green Reagent, which is weakly fluorescent while in a reduced state and exhibits bright green photostable fluorescence upon oxidation by ROS, with absorption/emission maxima of ~485/520 nm. The reactive CellROX fluorescence was significantly increased in the PARK2 neurons, indicating that there was increased ROS production (Figures 6I and 6J). These phenotypes were also observed in the neurons derived from aHDF-iPSCs (Figures 6F–6J). These results strongly suggest that the hiPSCs derived from patient T cells can be used as a model of neurological disease.

DISCUSSION

hiPSCs can be used to generate neuronal cells in vitro, which has widened the application of these cells. However, the requirement for skin biopsies to be performed must be overcome before iPSC technologies are widely adopted. Seki et al. (2010) developed TiPSCs, which enabled iPSCs to be obtained more easily and with less invasive methods. The current study shows that TiPSCs can differentiate into functional neurons, including dopaminergic, GABAergic, and glutamatergic subtypes, using an optimized differentiation protocol, dNS method. Moreover, these cells could be used to study the mechanisms underlying neuronal diseases, although there are some differences between TiPSCs and aHDF-iPSCs. Furthermore, the

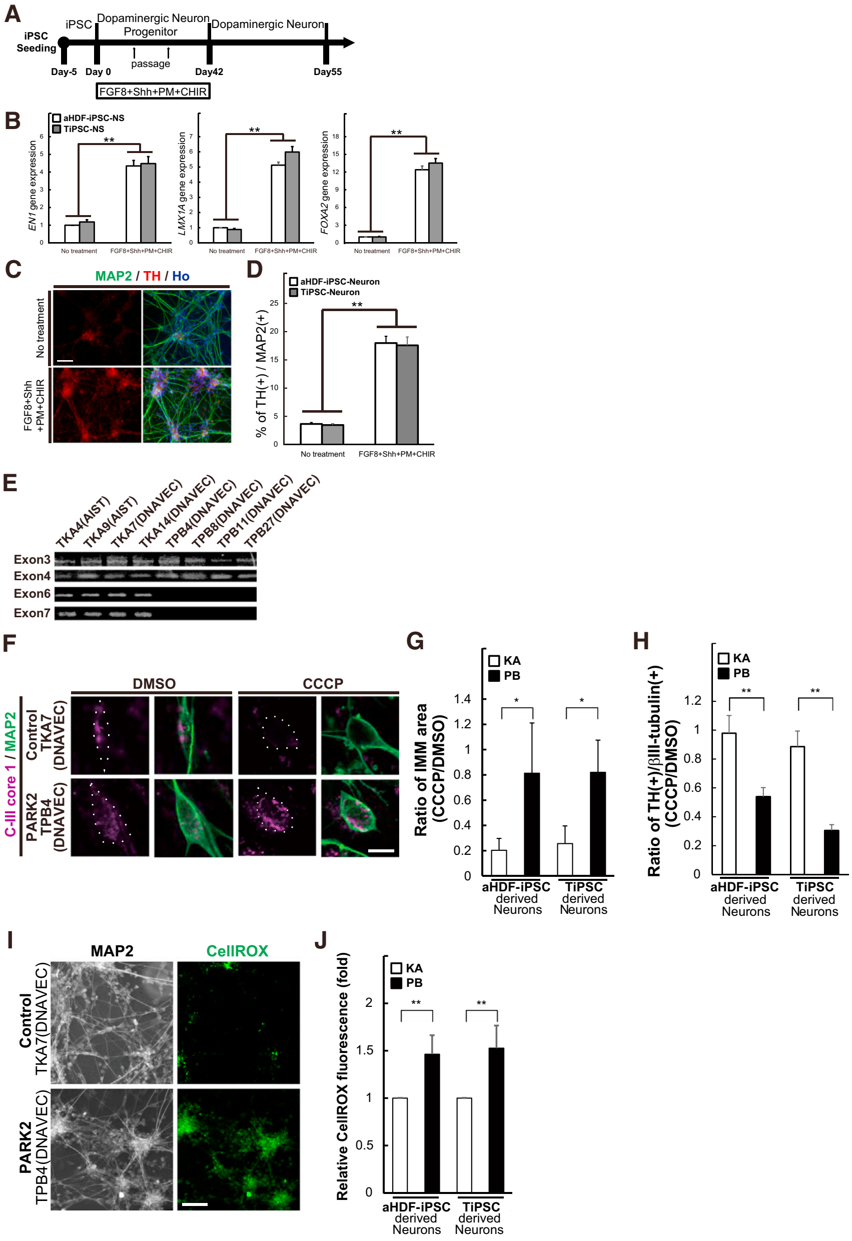

Figure 6. Neurons Derived from TiPSCs Established from a PARK2 Patient Exhibited Several Parkinson's Disease Phenotypes: Impairment of Mitochondrial Functions, Dopaminergic Neuron-Specific Cell Death, and Increased ROS Production

(A) An overview of the culture protocol used for the induction of midbrain dopaminergic neurons (mDANs) from hiPSCs.

(B) The results of a qPCR analysis of tertiary neurospheres treated with or without the four small molecules for markers of mDAN, including *EN1*, *LMX1A*, and *FOXA2*.

(C and D) The results of an immunocytochemical analysis of the tertiary neurospheres treated with or without the four small molecules for a dopaminergic neuronal marker, TH. Three experiments were performed for each group. Scale bars, 50 μm.

(E) Deletion of exons 6 and 7 was confirmed in clones TPB4 (DNAVEC), TPB8 (DNAVEC), TPB11 (DNAVEC), and TPB27 (DNAVEC).

(F and G) Carbonyl cyanide *m*-chlorophenyl hydrazone (CCCP) treatment reduced the inner mitochondrial membrane area (IMM, magenta) in control neurons, but not in PARK2 neurons. The area of the IMM in KA clone-derived MAP2-positive neurons (eKA3, TKA4 [AIST], TKA7 [DNAVEC]) was reduced following CCCP treatment. White dotted lines indicate cell bodies of neural cells. Scale bars, 25 μm. This reduction was not observed in PARK2 patient-derived MAP2-positive neurons (PB2, TPB4 [DNAVEC], and TPB8 [DNAVEC]). *p < 0.05 compared with the control. Significant differences were assessed using Student's t test. At least three experiments were performed per group, with 10–12 cells quantified per experiment.

(H) CCCP treatment significantly decreased the ratio of TH-positive neurons in PARK2 neurons compared with control neurons. At least three experiments were performed for each group, with 200–300 cells quantified per experiment.

(I and J) The results of an oxidative stress analysis using CellROX Green Reagent. PARK2 neurons showed increased fluorescence of CellROX compared with control neurons. At least three experiments were performed for each group, with 200–300 cells quantified per experiment. Scale bars, 50 μm. (B), (D), (H), (J) **p < 0.01 compared with the control. The significance of differences was assessed using Student's *t* test. Three experiments were performed for each group. aHDF-iPSCs, KA11, KA23, PB2, and PB20; TiPSCs, TKA7 [DNAVEC], TKA14 [DNAVEC], TPB4 [DNAVEC], and TPB8 (DNAVEC).

neurons differentiated from TiPSCs derived from a PARK2 patient exhibited several Parkinson's disease phenotypes, including an impairment of mitochondrial functions, dopaminergic neuron-specific cell death, and increased ROS production.

Most patients with neurological disorders, such as Parkinson's disease, do not have a genetic background associated with the onset of the disease. Recent whole-exome sequencing studies revealed that such sporadic diseases are related to many rare variants. To elucidate the etiologies

of these diseases, it is important to establish hiPSCs from a sufficient number of patients and to characterize multiple clones such that statistical analyses can be performed. T cells are a suitable source of hiPSCs to model sporadic neurological diseases owing to the ease with which they can be obtained. In this article, we have shown that neurons generated from TiPSCs exhibit phenotypes similar to those of aHDF-iPSCs modeling Parkinson's disease. We will therefore address in future work the question of whether TiPSCs can be used in the study of other neurological diseases.

Some studies indicate that iPSCs retain a transient transcriptional and epigenetic memory of their cell types of origin, which can substantially affect their potential to differentiate into various cell types, even among iPSCs that are genetically identical (Kim et al., 2010, 2011; Polo et al., 2010). Two independent studies of genetically matched hiPSC clones established from skin or blood cells examined how their differentiation potential is influenced by their cell types of origin. Daley and colleagues reported that blood cell-derived iPSCs differentiate into blood cells more efficiently than keratinocyte-derived iPSCs, and the latter iPSCs efficiently differentiate into keratinocytes (Kim et al., 2011). They concluded that the differentiation potential of hiPSCs is influenced by a residual epigenetic memory of the tissue from which they are derived. They also showed that extended passage of most iPSC lines reduced the effect of cell origin by erasing the cells' epigenetic memory, although some clones failed to erase the epigenetic memory, even after recurrent passaging. It is possible that our protocol could minimize the differences stemming from differences in epigenetic status after any passage of iPSCs, which we will address in a future study. However, a study by Yamanaka and colleagues reported that blood cell-derived iPSCs and skin cell-derived iPSCs can both differentiate into the hepatic lineage if they are established from the same donor (Kajiwara et al., 2012). They concluded that variations in hepatic differentiation are largely owing to differences in the donors, rather than to the cell types from which iPSCs are derived. Although these two conclusions seem contradictory, it is possible that the influences of epigenetic memory and donor differences vary according to the lineage along which cells differentiate. Here, we showed that TiPSCs failed to differentiate along the neural lineage using the EB formation protocol. The increased expression of mesendodermal and endodermal markers in TiPSC-derived EBs suggests that TiPSCs preferentially differentiated into the mesendodermal lineage, rather than the ectodermal lineage. Our single-cell dissociation protocol enabled TiPSCs to differentiate into functional neuronal cells with a similar efficiency as aHDF-iPSCs. Furthermore, TiPSC-derived neuronal cells generated from a Parkinson's disease patient exhibited abnormal mitochondrial degradation similar to aHDF-iPSC-derived neuronal cells. Our results suggest that this robust directed differentiation protocol can overcome the biased differentiation of iPSCs owing to epigenetic memory of the original cells. Our results also suggest that TCR rearrangement does not affect the differentiation efficiency when a directed neural differentiation method is used. EBs contain cells that have differentiated from PSCs into the three germ cell lineages in a spontaneous and unbiased manner (Itskovitz-Eldor et al., 2000). However, TiPSC-derived EBs contained few neural cells due to their reduced potential to differentiate into the neural lineage. By contrast, our differentiation protocol facilitated directed neural differentiation by removing extracellular signals. In relation to genetic background, Yamanaka and colleagues suggested that the propensity for hepatic differentiation of iPSCs is affected by differences in the donor, rather than in the original cell types (Kajiwara et al., 2012). Our results suggest that differences in the donors, original cell types, and iPSC generation methods can be minimalized by using this differentiation protocol.

Recently, several studies have suggested that peripheral blood mononuclear cells (PBMCs) are a promising resource for generating iPSCs because most of these cells do not undergo TCR rearrangement (Loh et al., 2010; Mack et al., 2011; Seki et al., 2010). However, PBMCs are composed of several cell types (e.g., T cells, B cells, and CD34 + progenitor cells), and each PBMC has an epigenetic memory of the cell from which it is derived. It is possible that PBMC-derived iPSCs restrict neural differentiation in a manner similar to TiPSCs. Since our present neural differentiation protocol was able to minimize the effect of cell origin, our protocol could be presumably applicable to iPSCs derived from other blood cell types.

Here, we suggest that the method of neural induction is the most important factor in the study of neural cells derived from human TiPSCs. The optimized induction protocol allows various iPSC clones to differentiate into functional neuronal cells, regardless of the cell types from which they are derived. Therefore, TiPSCs can be used to study neurological diseases and to recapitulate disease-specific phenotypes, comparable with iPSCs derived from fibroblasts or other cells, when generated using the optimized differentiation protocol. We propose that T cells are an ideal source of patient-specific iPSCs and will be widely used for neurological disease modeling. We believe that our present neural differentiation protocol will minimize the effect of cell origin and is applicable to iPSCs derived from other tissues. For example, urine epithelial cells could be an excellent alternate source for the generation of iPSCs because monocytes without TCR rearrangement can also be obtained in a less invasive fashion (Song et al., 2011; Zhang et al., 2015; Zhou et al., 2011).

These possibilities for modeling neurological diseases should be investigated in the future.

EXPERIMENTAL PROCEDURES

Neural Differentiation In Vitro

A previously reported protocol was used for neural differentiation of hiPSCs (Nori et al., 2011). Briefly, hiPSCs were dissociated and cultured in suspension as EBs, which were subsequently dissociated and cultured in MHM supplemented with B27 (Life Technologies), 20 ng/ml fibroblast growth factor 2 (FGF-2, Peprotech), and 10 ng/ml hLIF (Millipore).

Neural rosette differentiation was performed as previously described (Koch et al., 2009). Four-day-old EBs were transferred to poly-L-ornithine (PO)-coated tissue culture dishes and propagated in ITSfn medium (DMEM/F-12 [Wako] containing 25 μg/ml insulin [Wako], 100 μg/ml transferrin [Nakalai-tesque], 5 ng/ml selenite [Sigma-Aldrich], and 2.5 μg/ml fibronectin [Sigma-Aldrich]). Within 10 days, neural tube-like structures developed in the EB outgrowth.

For neural induction from single hiPSCs, hiPSCs were incubated with TrypLE Select (Life Technologies) for 5 min and dissociated into single cells by pipetting. Cells were plated into a T75 flask (Nunclon), then 10^3 cells were plated for the neurosphere formation assay, and cultured in MHM supplemented with B27, 20 ng/ml FGF-2, 10 μM Y-27632 (Wako), and 10 ng/ml hLIF in 4% oxygen for 14 days. Neurospheres were repeatedly passaged by dissociation into single cells, and then cultured in the same manner. Neurospheres at passages 3–7 were typically used for analysis. For terminal differentiation, dissociated neurospheres were allowed to adhere to PO- (Sigma-Aldrich) and fibronectin-coated coverslips and cultured in MHM containing B27, 10 ng/ml brain-derived neurotrophic factor (BDNF; R&D systems), 10 ng/ml glial cell-derived neurotrophic factor (GDNF; R&D systems), 200 μM ascorbic acid (Sigma-Aldrich), and 1 mM dibutyryl-cAMP (Sigma-Aldrich) for 10–70 days.

Ethics

For the use of human samples, human ethics approval was obtained by the Ethics Committee of Keio University School of Medicine.

ACCESSION NUMBERS

The accession number for both the microarray data and genomic methylation data reported in this paper is GEO: GSE76832.

SUPPLEMENTAL INFORMATION

Supplemental Information includes Supplemental Experimental Procedures, six figures, and three tables and can be found with this article online at http://dx.doi.org/10.1016/j.stemcr.2016.01.010.

AUTHOR CONTRIBUTIONS

T.M., K.F., W.A., and H.O. conceived and designed the experiments. T.M., K.F., T.A.-N., T.A, N.K. R.Y., M.T., H.T., K.I., M. Ishi, and M. Iso. performed the experiments and analyzed data. T.M., K.F., W.A., and H.O. wrote and edited the manuscript. T.K., M. Ohtaka, M. Ohyama, K.N., M.N., S.S., N.H., T.Y., Z.Z., H.K., and T.T. contributed reagents, materials, and analysis tools. All authors read and approved the final manuscript.

ACKNOWLEDGMENTS

The authors thank Dr. Hajime Komano and all the members of H.O.'s laboratory for their encouragement and support. This work was supported by funding from the Project for the Realization of Regenerative Medicine and Support for Core Institutes for iPS Cell Research from the Ministry of Education, Culture, Sports, Science and Technology of Japan (MEXT) to H.O., Research Center Network for Realization Research Centers/Projects of Regenerative Medicine (the Program for Intractable Disease Research utilizing disease-specific iPS Cells) from the Japan Science and Technology Agency (JST) and Japan Agency for Medical Research and Development (AMED) to H.O., the New Energy and Industrial Technology Development Organization (NEDO) to H.O. and W.A., the Japan Society for the Promotion of Science (JSPS) to W.A., and a Grant-in-Aid for the Global COE Program from MEXT to Keio University. H.O. is a scientific consultant for SanBio, Co. Ltd., Eisai, Co., Ltd.

Received: December 19, 2014
Revised: January 14, 2016
Accepted: January 15, 2016
Published: February 18, 2016

REFERENCES

Aoi, T., Yae, K., Nakagawa, M., Ichisaka, T., Okita, K., Takahashi, K., Chiba, T., and Yamanaka, S. (2008). Generation of pluripotent stem cells from adult mouse liver and stomach cells. Science *321*, 699–702.

Brennand, K.J., Simone, A., Jou, J., Gelboin-Burkhart, C., Tran, N., Sangar, S., Li, Y., Mu, Y., Chen, G., Yu, D., et al. (2011). Modelling schizophrenia using human induced pluripotent stem cells. Nature *473*, 221–225.

Chambers, S.M., Fasano, C.A., Papapetrou, E.P., Tomishima, M., Sadelain, M., and Studer, L. (2009). Highly efficient neural conversion of human ES and iPS cells by dual inhibition of SMAD signaling. Nat. Biotechnol. *27*, 275–280.

Clarke, L., and van der Kooy, D. (2009). Low oxygen enhances primitive and definitive neural stem cell colony formation by inhibiting distinct cell death pathways. Stem Cells *27*, 1879–1886.

Fusaki, N., Ban, H., Nishiyama, A., Saeki, K., and Hasegawa, M. (2009). Efficient induction of transgene-free human pluripotent stem cells using a vector based on Sendai virus, an RNA virus that does not integrate into the host genome. Proc. Jpn. Acad. Ser. B Phys. Biol. Sci. *85*, 348–362.

Hanks, M., Wurst, W., Anson-Cartwright, L., Auerbach, A.B., and Joyner, A.L. (1995). Rescue of the En-1 mutant phenotype by replacement of En-1 with En-2. Science *269*, 679–682.

Hunt, P., Gulisano, M., Cook, M., Sham, M.H., Faiella, A., Wilkinson, D., Boncinelli, E., and Krumlauf, R. (1991). A distinct Hox code for the branchial region of the vertebrate head. Nature *353*, 861–864.

Imaizumi, Y., and Okano, H. (2014). Modeling human neurological disorders with induced pluripotent stem cells. J. Neurochem. *129*, 388–399.

Imaizumi, Y., Okada, Y., Akamatsu, W., Koike, M., Kuzumaki, N., Hayakawa, H., Nihira, T., Kobayashi, T., Ohyama, M., Sato, S., et al. (2012). Mitochondrial dysfunction associated with increased oxidative stress and alpha-synuclein accumulation in PARK2 iPSC-derived neurons and postmortem brain tissue. Mol. Brain *5*, 35.

Itskovitz-Eldor, J., Schuldiner, M., Karsenti, D., Eden, A., Yanuka, O., Amit, M., Soreq, H., and Benvenisty, N. (2000). Differentiation of human embryonic stem cells into embryoid bodies compromising the three embryonic germ layers. Mol. Med. *6*, 88–95.

Kajiwara, M., Aoi, T., Okita, K., Takahashi, R., Inoue, H., Takayama, N., Endo, H., Eto, K., Toguchida, J., Uemoto, S., et al. (2012). Donor-dependent variations in hepatic differentiation from human-induced pluripotent stem cells. Proc. Natl. Acad. Sci. USA *109*, 12538–12543.

Kim, K., Doi, A., Wen, B., Ng, K., Zhao, R., Cahan, P., Kim, J., Aryee, M.J., Ji, H., Ehrlich, L.I., et al. (2010). Epigenetic memory in induced pluripotent stem cells. Nature *467*, 285–290.

Kim, K., Zhao, R., Doi, A., Ng, K., Unternaehrer, J., Cahan, P., Huo, H., Loh, Y.H., Aryee, M.J., Lensch, M.W., et al. (2011). Donor cell type can influence the epigenome and differentiation potential of human induced pluripotent stem cells. Nat. Biotechnol. *29*, 1117–1119.

Koch, P., Opitz, T., Steinbeck, J.A., Ladewig, J., and Brustle, O. (2009). A rosette-type, self-renewing human ES cell-derived neural stem cell with potential for in vitro instruction and synaptic integration. Proc. Natl. Acad. Sci. USA *106*, 3225–3230.

Loh, Y.H., Hartung, O., Li, H., Guo, C., Sahalie, J.M., Manos, P.D., Urbach, A., Heffner, G.C., Grskovic, M., Vigneault, F., et al. (2010). Reprogramming of T cells from human peripheral blood. Cell Stem Cell *7*, 15–19.

Mack, A.A., Kroboth, S., Rajesh, D., and Wang, W.B. (2011). Generation of induced pluripotent stem cells from CD34+ cells across blood drawn from multiple donors with non-integrating episomal vectors. PLoS One *6*, e27956.

Marchetto, M.C., and Gage, F.H. (2012). Modeling brain disease in a dish: really? Cell Stem Cell *10*, 642–645.

Nishimura, K., Sano, M., Ohtaka, M., Furuta, B., Umemura, Y., Nakajima, Y., Ikehara, Y., Kobayashi, T., Segawa, H., Takayasu, S., et al. (2011). Development of defective and persistent Sendai virus vector: a unique gene delivery/expression system ideal for cell reprogramming. J. Biol. Chem. *286*, 4760–4771.

Nori, S., Okada, Y., Yasuda, A., Tsuji, O., Takahashi, Y., Kobayashi, Y., Fujiyoshi, K., Koike, M., Uchiyama, Y., Ikeda, E., et al. (2011). Grafted human-induced pluripotent stem-cell-derived neurospheres promote motor functional recovery after spinal cord injury in mice. Proc. Natl. Acad. Sci. USA *108*, 16825–16830.

Okita, K., Yamakawa, T., Matsumura, Y., Sato, Y., Amano, N., Watanabe, A., Goshima, N., and Yamanaka, S. (2013). An efficient nonviral method to generate integration-free human-induced pluripotent stem cells from cord blood and peripheral blood cells. Stem Cells *31*, 458–466.

Oliver, G., Mailhos, A., Wehr, R., Copeland, N.G., Jenkins, N.A., and Gruss, P. (1995). Six3, a murine homologue of the sine oculis gene, demarcates the most anterior border of the developing neural plate and is expressed during eye development. Development *121*, 4045–4055.

Panopoulos, A.D., Yanes, O., Ruiz, S., Kida, Y.S., Diep, D., Tautenhahn, R., Herrerias, A., Batchelder, E.M., Plongthongkum, N., Lutz, M., et al. (2012). The metabolome of induced pluripotent stem cells reveals metabolic changes occurring in somatic cell reprogramming. Cell Res. *22*, 168–177.

Polo, J.M., Liu, S., Figueroa, M.E., Kulalert, W., Eminli, S., Tan, K.Y., Apostolou, E., Stadtfeld, M., Li, Y., Shioda, T., et al. (2010). Cell type of origin influences the molecular and functional properties of mouse induced pluripotent stem cells. Nat. Biotechnol. *28*, 848–855.

Seki, T., Yuasa, S., Oda, M., Egashira, T., Yae, K., Kusumoto, D., Nakata, H., Tohyama, S., Hashimoto, H., Kodaira, M., et al. (2010). Generation of induced pluripotent stem cells from human terminally differentiated circulating T cells. Cell Stem Cell *7*, 11–14.

Shimura, H., Hattori, N., Kubo, S., Mizuno, Y., Asakawa, S., Minoshima, S., Shimizu, N., Iwai, K., Chiba, T., Tanaka, K., et al. (2000). Familial Parkinson disease gene product, parkin, is a ubiquitin-protein ligase. Nat. Genet. *25*, 302–305.

Simeone, A., Acampora, D., Gulisano, M., Stornaiuolo, A., and Boncinelli, E. (1992). Nested expression domains of four homeobox genes in developing rostral brain. Nature *358*, 687–690.

Song, B., Niclis, J.C., Alikhan, M.A., Sakkal, S., Sylvain, A., Kerr, P.G., Laslett, A.L., Bernard, C.A., and Ricardo, S.D. (2011). Generation of induced pluripotent stem cells from human kidney mesangial cells. J. Am. Soc. Nephrol. *22*, 1213–1220.

Takahashi, K., Tanabe, K., Ohnuki, M., Narita, M., Ichisaka, T., Tomoda, K., and Yamanaka, S. (2007). Induction of pluripotent stem cells from adult human fibroblasts by defined factors. Cell *131*, 861–872.

Tropepe, V., Hitoshi, S., Sirard, C., Mak, T.W., Rossant, J., and van der Kooy, D. (2001). Direct neural fate specification from embryonic stem cells: a primitive mammalian neural stem cell stage acquired through a default mechanism. Neuron *30*, 65–78.

Watanabe, K., Ueno, M., Kamiya, D., Nishiyama, A., Matsumura, M., Wataya, T., Takahashi, J.B., Nishikawa, S., Nishikawa, S., Muguruma, K., et al. (2007). A ROCK inhibitor permits survival of dissociated human embryonic stem cells. Nat. Biotechnol. *25*, 681–686.

Zhang, S.Z., Li, H.F., Ma, L.X., Qian, W.J., Wang, Z.F., and Wu, Z.Y. (2015). Urine-derived induced pluripotent stem cells as a modeling tool for paroxysmal kinesigenic dyskinesia. Biol. Open *4*, 1744–1752.

Zhou, T., Benda, C., Duzinger, S., Huang, Y., Li, X., Li, Y., Guo, X., Cao, G., Chen, S., Hao, L., et al. (2011). Generation of induced pluripotent stem cells from urine. J. Am. Soc. Nephrol. *22*, 1221–1228.

Zhou, Z., Kohda, K., Ibata, K., Kohyama, J., Akamatsu, W., Yuzaki, M., Okano, H.J., Sasaki, E., and Okano, H. (2014). Reprogramming non-human primate somatic cells into functional neuronal cells by defined factors. Mol. Brain *7*, 24.

Stem Cell Reports, Volume *6*

Supplemental Information

Functional Neurons Generated from T Cell-Derived Induced Pluripotent Stem Cells for Neurological Disease Modeling

Takuya Matsumoto, Koki Fujimori, Tomoko Andoh-Noda, Takayuki Ando, Naoko Kuzumaki, Manabu Toyoshima, Hirobumi Tada, Kent Imaizumi, Mitsuru Ishikawa, Ryo Yamaguchi, Miho Isoda, Zhi Zhou, Shigeto Sato, Tetsuro Kobayashi, Manami Ohtaka, Ken Nishimura, Hiroshi Kurosawa, Takeo Yoshikawa, Takuya Takahashi, Mahito Nakanishi, Manabu Ohyama, Nobutaka Hattori, Wado Akamatsu, and Hideyuki Okano

Supplemental Figure Legends

Fig. S1 Characterization of the genomic profile of iPSCs derived from T-cells and adult human dermal fibroblasts

(A) A reverse-transcription PCR analysis showed the loss of the Sendai-virus (SeV) sequence in TiPSCs.

(B)The copy number profiles of whole chromosomes in TiPSCs and aHDF-iPSCs were assessed by a comparative genomic hybridization microarray analysis. There was no evidence that genomic aberrations were introduced during the process of establishing these iPSCs.

(C) Characterization of the T-cell receptor-β rearrangement by capillary electrophoresis. The green line was derived from the band of the Jβ1 gene, and the blue line was derived from the band of the Jβ2 gene. TKA4(AIST) showed rearrangements in Vβ/Jβ2 and Dβ/Jβ. TKA9(AIST) showed rearrangements in Vβ/Jβ1, 2. TPB4(DNAVEC) showed rearrangements in Vβ/Jβ2 and Dβ/Jβ. TPB8(DNAVEC) showed rearrangements in Vβ/Jβ1, 2 and Dβ/Jβ.

(D) Comparison of the global genomic methylation profiles of aHDF-iPSCs (KA11 and KA23) and TiPSCs (TKA4(AIST) and TKA9(AIST)). Hierarchical clustering analysis demonstrated the clear separation of aHDF-iPSCs and TiPSCs into two clusters.

Fig. S2. Comparison of the mRNA transcript levels in the EBs or DSi-EBs derived from TiPSCs and those derived from aHDF-iPSCs determined by qPCR.

(A) Overview of the culture protocols used for this experiment. (B) The expression of neural stem markers (*TUBB3*, *NESTIN*, *SOX1,* and *PAX6*) was higher, and (C) the expression of the mesendodermal marker *BRACHYURY* and the endodermal marker *SOX17* was lower in EBs derived from aHDF-iPSCs than in those derived from TiPSCs, even in the presence of dual SMAD inhibition (DSi) (n=3 independent experiments; mean ± SD; *$P < 0.05$, **$P < 0.01$; Student's *t*-test).

Fig. S3. Comparison of mRNA transcript levels in rosettes generated from TiPSCs and aHDF-iPSCs by quantitative reverse-transcription PCR. The expression of neural stem markers (*PAX6*, *NESTIN*, *SOX1*, *DACH1*, and *ZNF312*) was higher in aHDF-iPSCs than in TiPSCs, (n=5 independent experiments; mean ± SD.).

Fig. S4. The neurosphere formation using the dNS method was dependent on Y27632, and was enhanced by culturing the cells under reduced oxygen conditions (n=5 independent experiments; mean ± SD.).

Fig. S5. Characterization of TiPSCs derived from a PARK2 patient (PB) and a healthy donor (FK). All of the TPB clones and TFK clones were immunopositive for the pluripotent markers, SSEA4 (Green) and TRA-1-60 (Red). They also were differentiated into βIII-tubulin-positive neurons, including TH-positive dopaminergic neurons, via neurosphere formation using a slightly modified dNS method. Scale bars: 200 μm

(SSEA4 immunostaining, TRA-1-60 immunostaining, and neurospheres) and 50 µm (neurons).

Fig. S6. Electrophysiological analysis of TiPSC-derived neurons in comparison to aHDF-iPSC-derived them. (A) Voltage-dependent sodium and potassium currents in neurons derived from TKA4(AIST) and eKA3 clones. (B) Representative traces of TiPSC- and aHDF-iPSC-derived neurons membrane potential responding to step depolarization (-10 pA to +70 pA) by current injection (TiPSC: TKA4(AIST), aHDF-iPSC: eKA3)

Table S1. The gene ontology analysis of transcripts upregulated in TiPSC (eTKA4) vs aHDF-iPSC (eKA3). Only significant and non-redundant categories are shown (*p* value < 0.01) , related to Figure 1.

Table S2. Antibodies used for immunocytochemistry, related to Figures 1, 2, 3, 5, 6, S5

Table S3. Sequences of primers used for genomic PCR, quantitative PCR, and detection of the Sendai virus vector, related to Figures 6 and S1

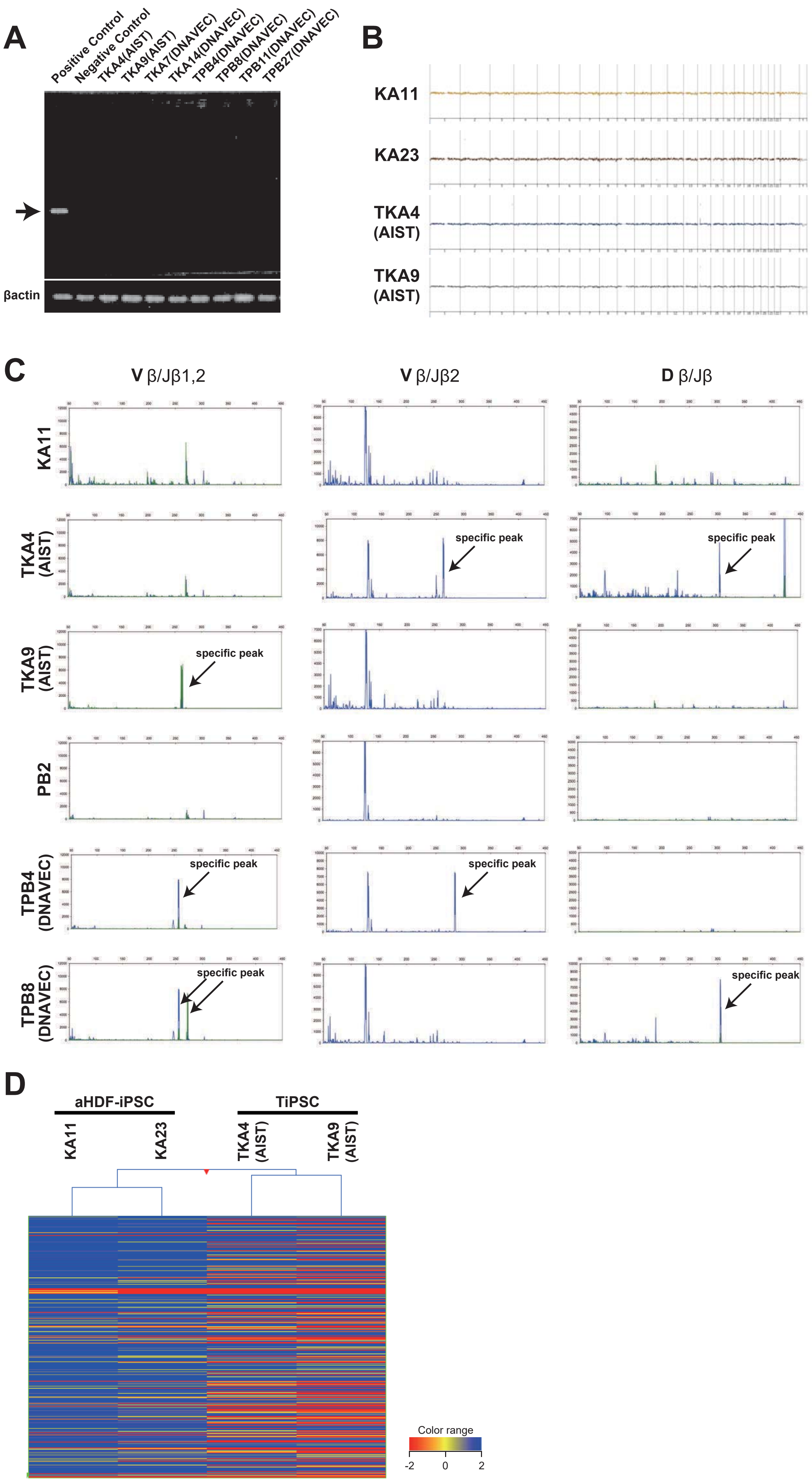
A
Positive Control
Negative Control
TKA4(AIST)
TKA9(AIST)
TKA7(DNAVEC)
TKA14(DNAVEC)
TPB4(DNAVEC)
TPB8(DNAVEC)
TPB11(DNAVEC)
TPB27(DNAVEC)
βactin
B
KA11
KA23
TKA4 (AIST)
TKA9 (AIST)
C
V β/Jβ1,2
V β/Jβ2
D β/Jβ
KA11
TKA4 (AIST)
TKA9 (AIST)
PB2
TPB4 (DNAVEC)
TPB8 (DNAVEC)
specific peak
D
aHDF-iPSC
TiPSC
KA11
KA23
TKA4 (AIST)
TKA9 (AIST)
Color range
-2
0
2

Fig.S1

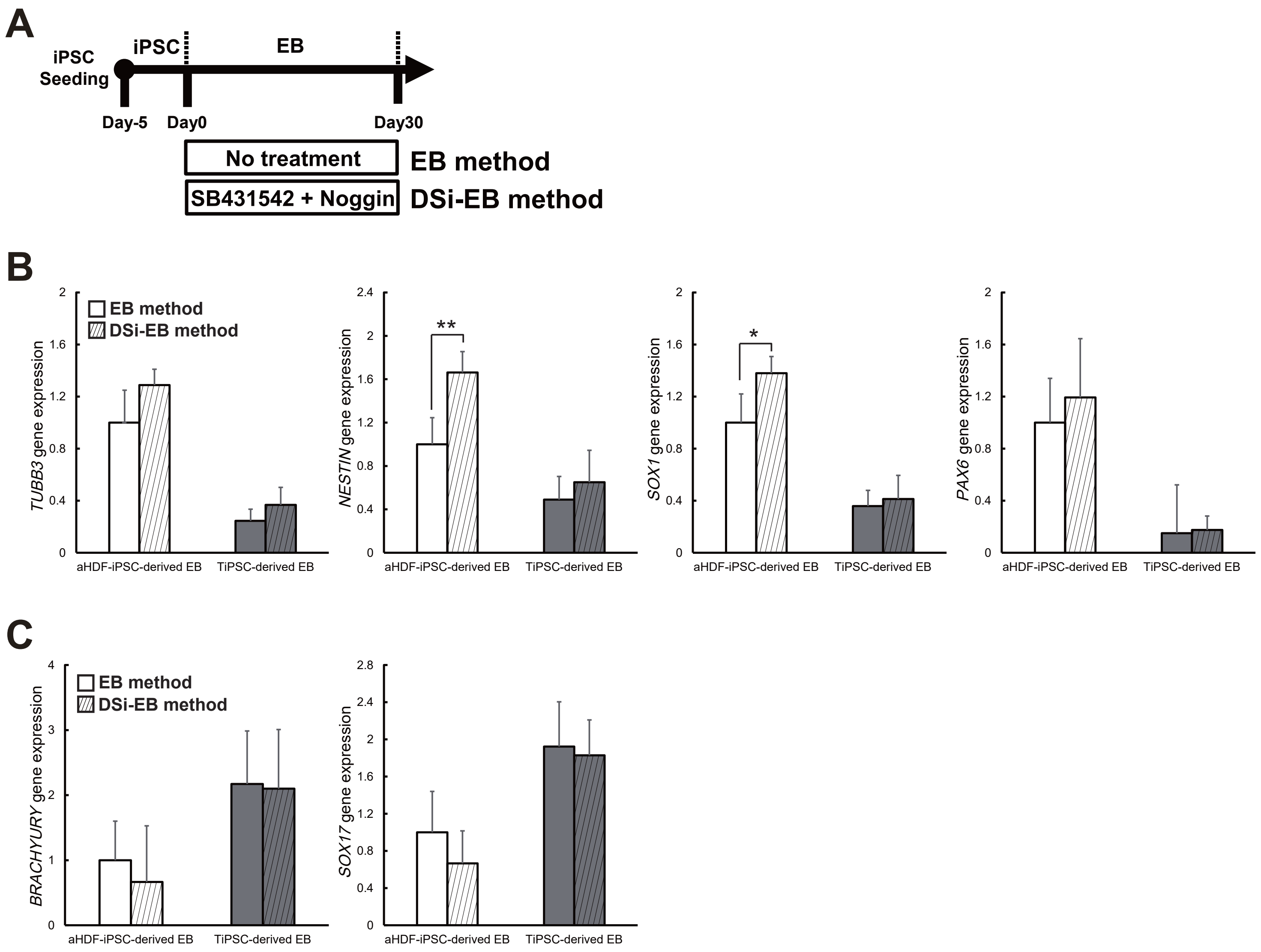
A
iPSC Seeding
iPSC
EB
Day-5
Day0
Day30
No treatment
EB method
SB431542 + Noggin
DSi-EB method
B
EB method
DSi-EB method
TUBB3 gene expression
NESTIN gene expression
SOX1 gene expression
PAX6 gene expression
aHDF-iPSC-derived EB
TiPSC-derived EB
C
BRACHYURY gene expression
SOX17 gene expression

Fig.S2

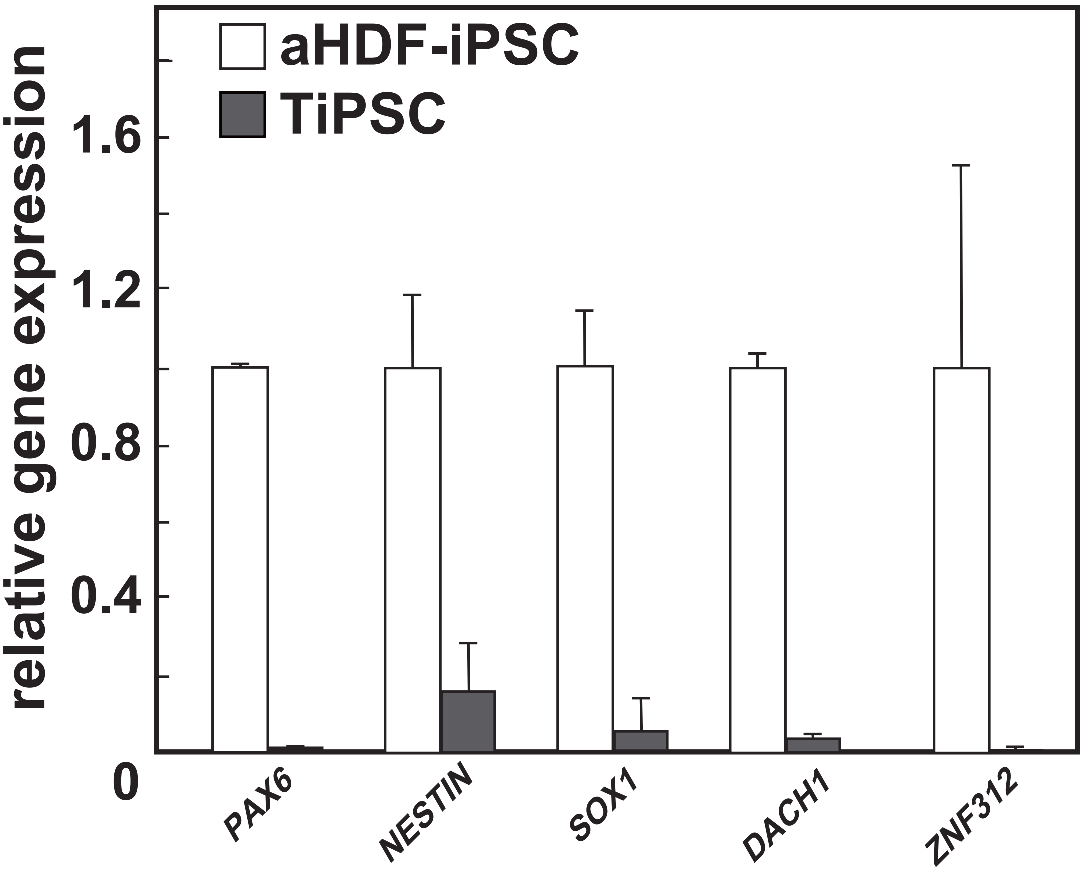
aHDF-iPSC
TiPSC
relative gene expression
1.6
1.2
0.8
0.4
0
PAX6
NESTIN
SOX1
DACH1
ZNF312

Fig.S3

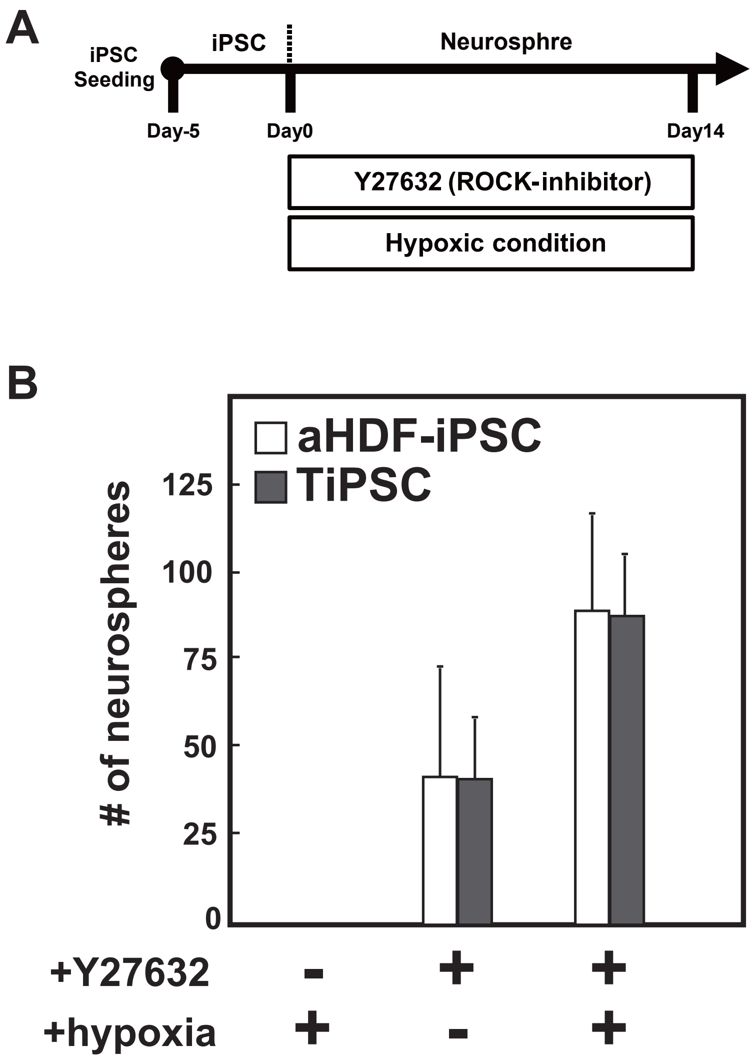
A
iPSC
Seeding
iPSC
Neurosphre
Day-5
Day0
Day14
Y27632 (ROCK-inhibitor)
Hypoxic condition
B
aHDF-iPSC
TiPSC
# of neurospheres
125
100
75
50
25
0
+Y27632
+hypoxia
-
+
+
+
-
+

Fig.S4

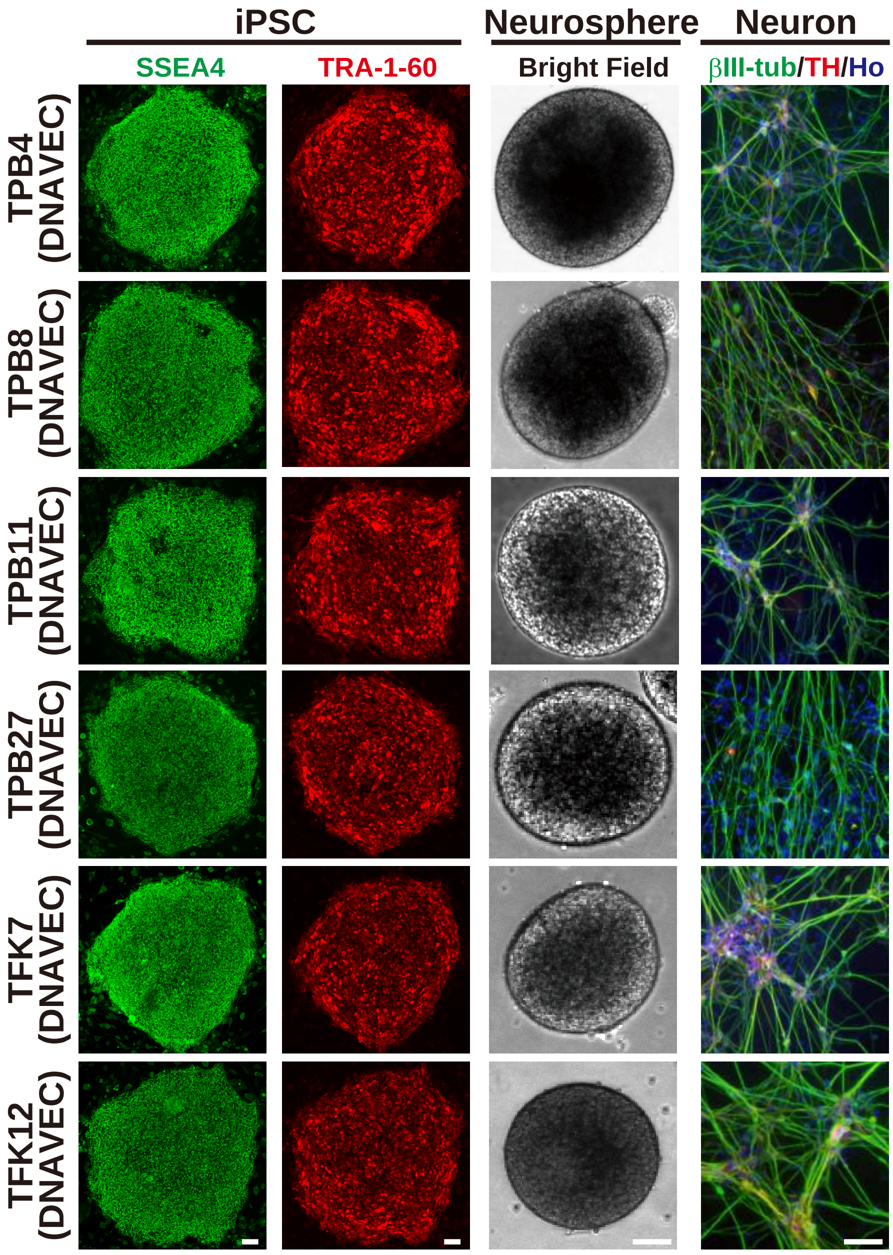

iPSC
Neurosphere
Neuron
SSEA4
TRA-1-60
Bright Field
βIII-tub/TH/Ho
TPB4 (DNAVEC)
TPB8 (DNAVEC)
TPB11 (DNAVEC)
TPB27 (DNAVEC)
TFK7 (DNAVEC)
TFK12 (DNAVEC)

Fig.S5

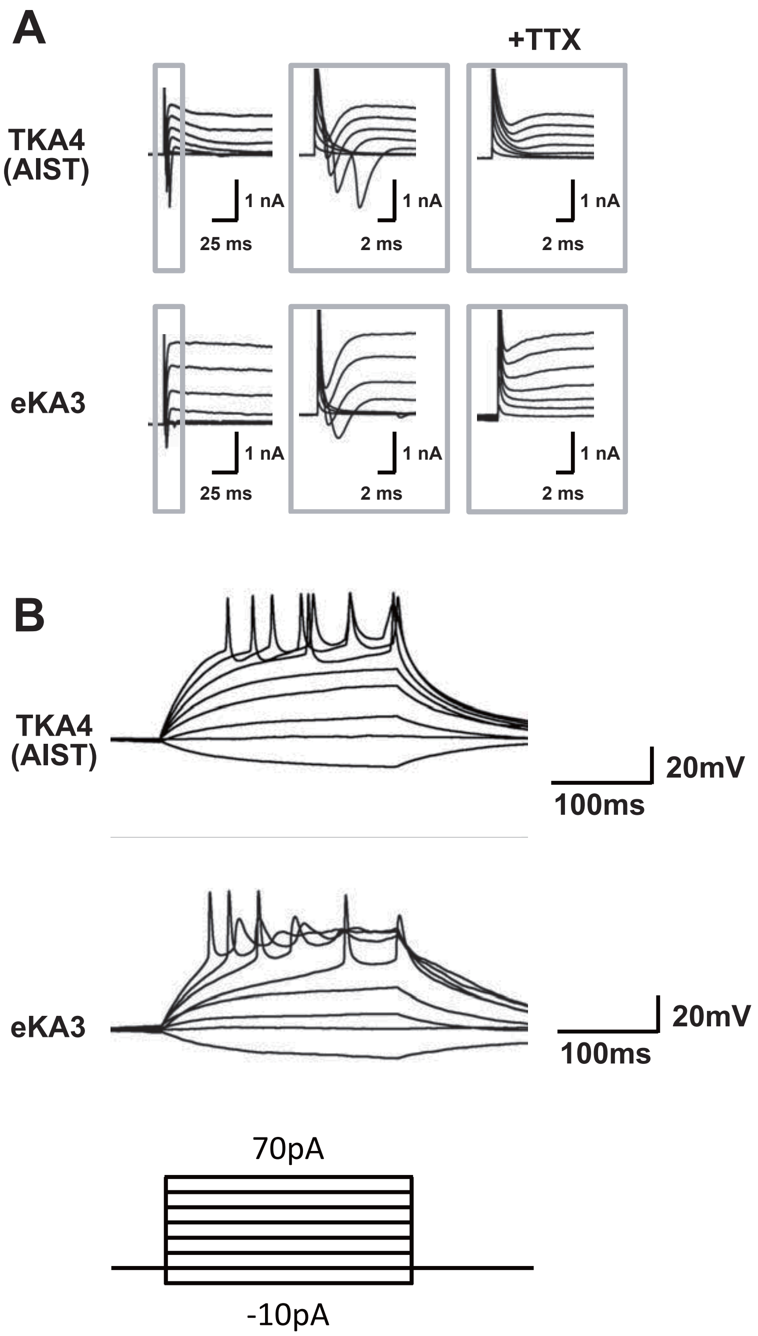
A
+TTX
TKA4
(AIST)
1 nA
25 ms
1 nA
2 ms
1 nA
2 ms
eKA3
1 nA
25 ms
1 nA
2 ms
1 nA
2 ms
B
TKA4
(AIST)
20mV
100ms
eKA3
20mV
100ms
70pA
-10pA

Fig.S6

GO ACCESSION	GO Term	corrected p-value
GO:0050896|GO:0051869	response to stimulus	2.35E-09
GO:0051716	cellular response to stimulus	2.70E-05
GO:0048583	regulation of response to stimulus	2.90E-05
GO:0010033	response to organic substance	4.18E-05
GO:0007165|GO:0023033	signal transduction	4.35E-05
GO:0042605|GO:0042606|GO:0042607	peptide antigen binding	7.22E-05
GO:0044700	single organism signaling	9.22E-05
GO:0023052|GO:0023046	signaling	9.22E-05
GO:0005685	U1 snRNP	9.22E-05
GO:0048518|GO:0043119	positive regulation of biological process	1.13E-04
GO:0006952|GO:0002217|GO:0042829	defense response	1.56E-04
GO:0006955	immune response	1.77E-04
GO:0007154	cell communication	2.04E-04
GO:0005515|GO:0045308	protein binding	2.56E-04
GO:0005686	U2 snRNP	2.56E-04
GO:0006950	response to stress	3.02E-04
GO:0044763	single-organism cellular process	3.21E-04
GO:0060968	regulation of gene silencing	3.28E-04
GO:0050778	positive regulation of immune response	3.53E-04
GO:0002429	immune response-activating cell surface receptor signaling pathway	3.71E-04
GO:0050776	regulation of immune response	4.67E-04
GO:0009719	response to endogenous stimulus	5.88E-04
GO:0003823	antigen binding	7.78E-04
GO:0007166	cell surface receptor signaling pathway	0.001009211
GO:0071310	cellular response to organic substance	0.001226054
GO:0055037	recycling endosome	0.001245703
GO:0034314	Arp2/3 complex-mediated actin nucleation	0.00135526
GO:0050789|GO:0050791	regulation of biological process	0.00135526
GO:0070887	cellular response to chemical stimulus	0.001424034
GO:0044699	single-organism process	0.001428615
GO:0065007	biological regulation	0.001428615
GO:0002768	immune response-regulating cell surface receptor signaling pathway	0.00162319
GO:0055038	recycling endosome membrane	0.00162319
GO:0002376	immune system process	0.001824635
GO:0002682	regulation of immune system process	0.0020203
GO:0004872|GO:0019041	receptor activity	0.0020203
GO:0042221	response to chemical	0.002038446
GO:0043014	alpha-tubulin binding	0.002862832
GO:0031295	T cell costimulation	0.002919185
GO:0009725	response to hormone	0.002942639
GO:0071944	cell periphery	0.003043802
GO:0048584	positive regulation of response to stimulus	0.003043802
GO:0031294	lymphocyte costimulation	0.00306639
GO:0045010	actin nucleation	0.003145426
GO:0032879	regulation of localization	0.003830532
GO:0045834	positive regulation of lipid metabolic process	0.004213654
GO:0005886|GO:0005904	plasma membrane	0.004333442
GO:0042060	wound healing	0.005252767
GO:0044459	plasma membrane part	0.005423789
GO:0002757	immune response-activating signal transduction	0.005748065
GO:0046983	protein dimerization activity	0.005748065
GO:0002684	positive regulation of immune system process	0.006020954
GO:0050794|GO:0051244	regulation of cellular process	0.006485721
GO:0050851	antigen receptor-mediated signaling pathway	0.006765673
GO:0051239	regulation of multicellular organismal process	0.006765784
GO:0042611	MHC protein complex	0.006801865
GO:0071407	cellular response to organic cyclic compound	0.006972198
GO:0051249	regulation of lymphocyte activation	0.007141397
GO:0050852	T cell receptor signaling pathway	0.007170936
GO:0005887	integral component of plasma membrane	0.008697007
GO:0048522|GO:0051242	positive regulation of cellular process	0.008868844
GO:0002253	activation of immune response	0.009484018

Table S1

Antibody	Dilution	Source	Catalogue number	Location
TRA-1-60	1:1000	Millipore	MAB4360	Billerica, MA
SSEA4	1:1000	Abcam	ab16287	Cambridge, MA
PAX6	1:500	BioLegend	901301	San Diego, CA
βIII-tubulin	1:2000	Sigma-Aldrich	T8660	St Louis, MI
MAP2	1:1000	Sigma-Aldrich	M4403	St Louis, MI
VGLUT1	1:4000	Synaptic Systems	135 303	Germany
GFAP	1:4000	DAKO	Z033401	Glostrup, DK
Synaptophysin	1:1000	Sigma-Aldrich	S5768	St Louis, MI
GABA	1:2000	Sigma-Aldrich	A2052	St Louis, MI
TH	1:500	Millipore	AB152	Billerica, MA
FOXG1	1:250	Abcam	ab18259	Cambridge, MA
OTX2	1:100	R&D Systems	AF1979	Minneapolis, MN
EN1	1:100	Abcam	ab117549	Cambridge, MA
HOXB4	1:100	DSHB	I12	Iowa City, IA
Complex III-core I	1:200	Thermo Fisher Scientific	459140	Waltham, MA

SSEA4, stage-specific embryonic antigen 4; MAP2, microtubule-associated protein 2; VGLUT1, vesicular glutamate transporter 1; GFAP, glial fibrillary acidic protein; GABA, gamma-aminobutyric acid; TH, tyrosine hydroxylase

Table S2

Name	Sequence
	**For genomic PCR**
Ex3 forward	ACATGTCACTTTTGCTTCCCT
Ex3 reverse	AGGCCATGCTCCATGCAGACTGC
Ex4 forward	AGGTAGATCAATCTACAACAGCT
Ex4 reverse	CTGGGTCAAGGTGAGCGTTGCCTGC
Ex6 forward	AGAGATTGTTTACTGTGGAAACA
Ex6 reverse	GAGTGATGCTATTTTTAGATCCT
Ex7 forward	GAGCCCCGTCCTGGTTTTCC
Ex7 reverse	CCACACAAGGCAGGGAGTAGCCAA
	**For qPCR**
OCT4 forward	TTGGGCTCGAGAAGGATGTGGT
OCT4 reverse	TGCATAGTCGCTGCTTGATCGC
NANOG forward	TGAACCTCAGCTACAAACAG
NANOG reverse	TGGTGGTAGGAAGAGTAAAG
PAX6 forward	ACCACACCGGTTTCCTCCTTCACA
PAX6 reverse	TTGCCATGGTGAAGCTGGGCAT
NESTIN forward	TTCCCTCAGCTTTCAGGACCCCAA
NESTIN reverse	AAGGCTGGCACAGGTGTCTCAA
SOX1 forward	GATCAGCAAGCGCCTGGGGG
SOX1 reverse	AGCAGCGTCTTGGTCTTGCGG
BRACHYURY forward	TGCTTCCCTGAGACCCAGTT
BRACHYURY reverse	GATCACTTCTTTCCTTTGCATCAAG
SOX17 forward	TGTCCCAAAACAGCTTCCTC
SOX17 reverse	TCACCCTTTTCGAGGATGAG
ACTB forward	TGAAGTGTGACGTGGACATC
ACTB reverse	GGAGGAGCAATGATCTTGAT
TUBB3 forward	ATTTCATCTTTGGTCAGAGTGGGGC
TUBB3 reverse	TGCAGGCAGTCGCAGTTTTCAC
DACH1 forward	CCGGTGGTGTGCAATGTGGAACAA
DACH1 reverse	GGTCTAGAACTTGCGTTGGTGCAGT
ZNF312 forward	GTGCGGCAAGGTGTTTAACGCT
ZNF312 reverse	ATTTTGTGCCTGCAGAGCGTGC
EN1 forward	AACCGCTACATCACGGAGCA
EN1 reverse	GATCTTGGCGCGCTTGTTCT
LMX1A forward	CAACTCAACAGAGGCGAGCATT
LMX1A reverse	GTTTTGGAACCACACCTGGAC
FOXA2 forward	CCATGCACTCGGCTTCCAG
FOXA2 reverse	TGTTGCTCACGGAGGAGTAG
	**For Sendai virus detection**
Sendai virus forward	AGACCCTAAGAGGACGAAGA
Sendai virus reverse	ACTCCCATGGCGTAACTCCATAGTG

Table S3

Supplemental Experimental Procedures

Isolation of human dermal fibroblasts (HDFs) and generation of aHDF-iPSCs

A skin-punch biopsy from a healthy 40-year-old Japanese male was used to generate aHDF-iPSCs (KA11, KA23, eKA3, and eKA4; passages 14-25). KA11 and KA23 were generated using retroviral vectors, and the other cell lines (eKA3 and eKA4) were generated using episomal plasmid vectors. aHDF-iPSCs from a PARK2 patient, ”PB” (PB2 and PB20; passages 8–17) were generated by retrovirus vectors, previously (Imaizumi et al., 2012). Maintenance of HDFs, lentiviral production, retroviral production, infection, episomal vector preparation, electroporation, stem cell culturing, and characterization were performed as described previously (Okita et al., 2011; Takahashi et al., 2007). All human primary cells were generated *in vitro* from tissue samples of human donors, with appropriate written informed consent given to the commercial providers. Skin biopsy and iPSC production procedures were approved by the Ethics Committee of Keio University School of Medicine (No. 20080016).

Isolation of human T-cells and generation of TiPSCs

Peripheral blood mononuclear cells (PBMCs) were obtained from two healthy donors, “KA” and “FK,” and one PARK2 patient, “PB,” by centrifuging heparinized blood over a Ficoll-Paque PREMIUM (GE Healthcare) gradient, according to the manufacturer’s instructions. CD3-positive cells were selected using a fluorescently conjugated anti-CD3 mAb (BD Pharmingen). PBMCs and cells subjected to fluorescence-activated cell sorting were cultured at 37°C in 5% CO_2 on a plate coated

with an anti-CD3 mAb in GT-T502 medium (KOHJIN BIO) containing 175 JRU/mL rIL-2. After 5 days of culture, activated PBMCs and activated T-cells were transferred to a 6-well plate coated with an anti-CD3 mAb at a density of 1.5 x 10^6 cells/well and incubated for an additional 24 hours. Thereafter, a solution containing SeV vectors was added to the wells. SeV vectors were obtained from DNAVEC Corp. or the National Institute of Advanced Industrial Science and Technology (AIST). The former vectors carried *OCT4*, *SOX2*, *KLF4*, or *c-MYC* (CytoTune^{TM}-iPS Reprogramming Kit) (Fusaki et al., 2009), whereas the latter vector carried all four reprogramming factor (Nishimura et al., 2011). At 24 hours post-infection, the medium was replaced with fresh GT-T502 medium. At 48 hours post-infection, cells were collected and transferred to a 10-cm dish containing mitomycin C-inactivated SNL feeder cells at a density of 5 x 10^4–5 x 10^5 cells/dish. After an additional 24 hours, the medium was replaced with hiPSC medium, which was changed every other day until colonies were picked. The generated hiPSCs were maintained on mitomycin C-inactivate SNL feeder cells in hiPSC medium. The healthy donor TiPSC lines, eTKA4 and eTKA5 (passages 12-19), were generated from KA-T-cells by episomal plasmid vectors. TKA4(AIST), TKA9(AIST), TKA7(DNAVEC), and TKA14(DNAVEC) (passages 14-20) were generated from KA-T-cells using SeV vectors made at each institution. TFK7(DNAVEC) and TFK12(DNAVEC) (passages 10-16) were generated from KF-T-cells using DNAVEC-SeV vectors. The PARK2 patient TiPSC lines, TPB4(DNAVEC), TPB8(DNAVEC), TPB11(DNAVEC), and TPB27(DNAVEC) (passages 14-20) were generated using DNAVEC-SeV vectors.

Immunocytochemical analysis of hiPSCs and hiPSC-derived neurons

Cells were fixed in phosphate-buffered saline (PBS) containing 4% paraformaldehyde for 30 minutes at room temperature. Thereafter, cells were incubated with the primary antibodies described in Table S2, washed with PBS, and incubated with an Alexa Fluor 488-, Alexa Fluor 555-, or Alexa Fluor 647-conjugated secondary antibody (1:500, Invitrogen). Images were obtained using an a universal fluorescence microscope (Axioplan2; Carl Zeiss) or confocal laser scanning microscope (LSM700; Carl Zeiss).

TCR clonotype mapping

Capillary electrophoresis was performed of genomic PCR products spanning TCRβ regions (Sandberg et al., 2005; van Dongen et al., 2003). TCRβ rearrangements were analyzed using BIOMED-2 protocols and a commercial kit (In VivoScribe Technologies) at Mitsubishi Chemical Medicine Corporation, Japan. The test comprises three multiplex master mixes that target conserved regions within the variable (V), diversity (D), and joining (J) regions.

Microarray analysis

RNA quality was assessed using a 2100 Bioanalyzer (Agilent Technologies). Total RNA (100 ng) was reverse-transcribed, labeled with biotin, and hybridized to a GeneChip® Human Genome U133 plus 2.0 Array (Affymetrix), which was subsequently washed and stained in a Fluidics Station 450 according to the manufacturer's instructions.

The microarrays were scanned using a GeneChip Scanner 3000 7G, and the raw image files were converted into normalized signal intensity values using the MAS 5.0 algorithm.

Targets were selected that (a) were called "present" in at least one of the eight arrays analyzed, and (b) had a probe intensity of ≥50. In total, 19,383 targets were identified from this initial screen. The normalized logs were hierarchically clustered based on uncentered correlation with complete linkage using Cluster 3.0 (Eisen et al., 1998) and visualized using Java TreeView (Saldanha, 2004). Principal component analysis (PCA) was performed by Spotfire DecisionSite 9.1.2 using normalized data. Genespring GX software (Agilent Technologies) was used for pairwise scatter plot analysis. A Venn diagram was constructed to visualize the 322 genes that were up- or downregulated in the aHDF-iPSC/TiPSC and iPSC (KA)/iPSC (PB) groups, and the 25 genes that were up- or downregulated in each of the NS groups (moderated t-test $P < 0.05$, fold change > 2.0). For the GO analysis, p values were calculated using Fisher's exact test.

Methylated DNA sequencing

Genomic DNA (1–2 μg) was fragmented by sonication using a Shearing System M220 (Covaris) and enriched using a CpG MethylQuest DNA Isolation Kit (Millipore) following the manufacturer's protocol. The chromatin immunoprecipitation sequencing (ChIP-seq) sample obtained after DNA enrichment was subjected to library preparation using the Illumina TruSeq™ ChIP Sample Prep Kit according to the manufacturer's protocol and quantified using a Hi-seq system. Obtained images were

analyzed and base-called using the BWA and SAM tools, and software was applied to align reads to the human genomic reference (hg19) at DNA CHIP RESERCH INC., Japan. Data were analyzed by Avadis NGS (Strand Scientific Intelligence).

Reverse-transcription-PCR

RNA isolation and reverse-transcription (RT)-PCR were performed as previously described (Okada et al., 2008). The amount of cDNA was normalized to the amount of β-actin mRNA. Quantitative RT-PCR (qPCR) was performed on an ABI PRISM Sequence detection System 7900HT (Applied BioSystems) using SYBR premix ExTaq Tli RNaseH Plus (Takara). The primers are described in Table S3.

PCR amplification of genomic DNA

Genomic DNA was purified from HDFs, T-cells, and hiPSCs using a DNeasy Kit (Qiagen). The PCR conditions were previously described (Table S3) (Kitada et al., 1998).

Lentiviral vector production

Lentiviral vector was produced as described previously (Zhou et al., 2014). 293T cells were transfected with the lentivirus plasmid, pCAG-HIVgp, and pCMV-VSV-G-RSV-Rev (Miyoshi et al., 1998) (kindly provided by Dr. Hiroyuki Miyoshi, RIKEN BRC, Japan). After 16–20 hours, the media was replaced and cells were incubated for a further 48–72 hours. The virus-containing media were collected and 0.45 μm filtered

followed by ultracentrifugation. The concentrated virus was suspended in PBS and stored at −80°C until use.

Electrophysiological analysis

For electrophysiological experiments, the culture medium was replaced with a physiological solution (118 mM NaCl, 2.5 mM KCl, 26 mM $NaHCO_3$, 1 mM NaH_2PO_4, 10 mM glucose, 4 mM $MgCl_2$, and 4 mM $CaCl_2$). Tetrodotoxin (TTX, 1 μM) was bath-applied. The electrodes (5-8 MΩ) were filled with whole-cell pipette solution (120 mM potassium acetate, 20 mM KCl, 0.1 mM $CaCl_2$, 5 mM $MgCl_2$, 0.2 mM EGTA, 5 mM ATP, and 10 mM HEPES, pH 7.3). The whole-cell recording of GFP-expressing neurons was configured using an EPC-7 amplifier (HEKA) and a Digidata 1200 acquisition board (Axon Instruments). The membrane potential was clamped at -60 mV. Membrane resistance (Rm), series resistance (Rs), and membrane capacitance (Cm) were monitored. Only recordings with Rm > 100 MΩ and Rs < 20 MΩ were included in the analysis.

To obtain evoked synaptic currents from hiPSC-derived neurons, concentric tungsten stimulating electrodes were placed ~100 μm from the neuron being recorded. Synaptic AMPA receptor-mediated responses at -60 mV were averaged over 30 trials(Tada et al., 2013; Tada et al., 2010).

Carbonyl cyanide m-chlorophenyl hydrazone treatment

Neurons were cultured with 30 μM Carbonyl cyanide m-chlorophenyl hydrazone (CCCP) (Sigma-Aldrich) or DMSO for 48 hours. Thereafter, cells were fixed,

stained for βIII-tubulin and Complex-III Core I, and counterstained with Hoechst. To quantify the inner mitochondrial membrane (IMM) area of neurons, the cytoplasmic area was extracted as shown in Figure 6F. Complex-III Core I-positive signals within the extracted area were converted to grayscale and digitized. The IMM area was quantified from the digitized values using ImageJ software.

Oxidative stress analysis

The ROS levels were determined by measuring the CellROX fluorescence using the CellROX® Green Reagent for oxidative stress detection (Life Technologies). Briefly, neurons were incubated with the CellROX® Reagent for 30 min at 37°C, after which they were washed with PBS and then fixed by 4% PFA for 30 minutes at room temperature. Thereafter, the cells were incubated with the primary antibody, MAP2 (1:1000, Sigma), overnight at 4°C, washed with PBS, and incubated with an Alexa Fluor 555-conjugated secondary antibody (1:500, Invitrogen) for 1 hr at room temperature. The fluorescence in the MAP2-positive neurons was measured by an IN Cell Analyzer 6000 (GE Healthcare Biosciences).

Supplimental Reference

Eisen, M.B., Spellman, P.T., Brown, P.O., and Botstein, D. (1998). Cluster analysis and display of genome-wide expression patterns. Proc Natl Acad Sci U S A *95*, 14863-14868.

Kitada, T., Asakawa, S., Hattori, N., Matsumine, H., Yamamura, Y., Minoshima, S., Yokochi, M., Mizuno, Y., and Shimizu, N. (1998). Mutations in the parkin gene cause autosomal recessive juvenile parkinsonism. Nature *392*, 605-608.

Miyoshi, H., Blomer, U., Takahashi, M., Gage, F.H., and Verma, I.M. (1998). Development of a self-inactivating lentivirus vector. J Virol *72*, 8150-8157.

Development of defective and persistent Sendai virus vector: a unique gene delivery/expression system ideal for cell reprogramming. J Biol Chem *286*, 4760-4771.

Okita, K., Matsumura, Y., Sato, Y., Okada, A., Morizane, A., Okamoto, S., Hong, H., Nakagawa, M., Tanabe, K., Tezuka, K., et al. (2011). A more efficient method to generate integration-free human iPS cells. Nat Methods *8*, 409-412.

Saldanha, A.J. (2004). Java Treeview--extensible visualization of microarray data. Bioinformatics *20*, 3246-3248.

Sandberg, Y., van Gastel-Mol, E.J., Verhaaf, B., Lam, K.H., van Dongen, J.J., and Langerak, A.W. (2005). BIOMED-2 multiplex immunoglobulin/T-cell receptor polymerase chain reaction protocols can reliably replace Southern blot analysis in routine clonality diagnostics. J Mol Diagn *7*, 495-503.

Tada, H., Kuroki, Y., Funabashi, T., Kamiya, Y., Goto, T., Suyama, K., Sano, A., Mitsushima, D., Etgen, A.M., and Takahashi, T. (2013). Phasic synaptic incorporation of GluR2-lacking AMPA receptors at gonadotropin-releasing hormone neurons is involved in the generation of the luteinizing hormone surge in female rats. Neuroscience *248*, 664-669.

Tada, H., Okano, H.J., Takagi, H., Shibata, S., Yao, I., Matsumoto, M., Saiga, T., Nakayama, K.I., Kashima, H., Takahashi, T., et al. (2010). Fbxo45, a novel ubiquitin ligase, regulates synaptic activity. The Journal of biological chemistry *285*, 3840-3849.

van Dongen, J.J., Langerak, A.W., Bruggemann, M., Evans, P.A., Hummel, M., Lavender, F.L., Delabesse, E., Davi, F., Schuuring, E., Garcia-Sanz, R., et al. (2003). Design and standardization of PCR primers and protocols for detection of clonal immunoglobulin and T-cell receptor gene recombinations in suspect lymphoproliferations: report of the BIOMED-2 Concerted Action BMH4-CT98-3936. Leukemia *17*, 2257-2317.
